# Microbiome dysbiosis and endometriosis: a systematic scoping review of current literature and knowledge gaps

**DOI:** 10.1093/hropen/hoaf061

**Published:** 2025-10-01

**Authors:** Federica Facciotti, Giorgia Di Stefano, Paola Maragno, Claudia Ferraro, Dhouha Dridi, Edgardo Somigliana, Paola Viganò, Paolo Vercellini, Maíra Casalechi

**Affiliations:** Department of Biotechnology and Bioscience, Università degli studi Milano-Bicocca, Milan, Italy; Infertility Unit, Fondazione IRCCS Ca’Granda Ospedale Maggiore Policlinico di Milano, Milan, Italy; Gastroenterology and Endoscopy Unit, Fondazione IRCCS Ca’ Granda Ospedale Maggiore Policlinico, Milan, Italy; Infertility Unit, Fondazione IRCCS Ca’Granda Ospedale Maggiore Policlinico di Milano, Milan, Italy; Gynecology Unit, Fondazione IRCCS Ca’ Granda Ospedale Maggiore Policlinico, Milan, Italy; Infertility Unit, Fondazione IRCCS Ca’Granda Ospedale Maggiore Policlinico di Milano, Milan, Italy; Department of Clinical Sciences and Community Health, Università degli Studi di Milano, Milan, Italy; Infertility Unit, Fondazione IRCCS Ca’Granda Ospedale Maggiore Policlinico di Milano, Milan, Italy; Gynecology Unit, Fondazione IRCCS Ca’ Granda Ospedale Maggiore Policlinico, Milan, Italy; Department of Clinical Sciences and Community Health, Università degli Studi di Milano, Milan, Italy; Infertility Unit, Fondazione IRCCS Ca’Granda Ospedale Maggiore Policlinico di Milano, Milan, Italy

**Keywords:** endometriosis, bacterial infection, microbiome, microbiota, dysbiosis, scoping review

## Abstract

**STUDY QUESTION:**

What is the evidence available concerning gut and reproductive tract microbiomes in patients with endometriosis and what are the methodological approaches employed in microbiome studies on endometriosis?

**SUMMARY ANSWER:**

The taxonomic profiles exhibited pronounced heterogeneity within women with and also within women without endometriosis across reviewed studies for all the anatomical districts evaluated.

**WHAT IS KNOWN ALREADY:**

Both human and animal studies support differences in the microbiome composition of individuals with and without endometriosis. Endometriosis onset occurs with variable symptoms and manifestations. The microbiome composition at different sites may contribute to this variability.

**STUDY DESIGN, SIZE, DURATION:**

We used the scoping review methodology. Systematic searches of studies from the PubMed, EMBASE, and Web of Science databases published between 1 January 2016 and 1 November 2024 addressing endometriosis microbiome characterization in: (i) gut, (ii) vaginal fluid, (iii) cervical fluid, (iv) peritoneal fluid, (v) uterine fluid, (vi) ovarian cyst fluid, (vii) oropharyngeal fluid, and (viii) eutopic and (ix) ectopic tissues were performed using a combination of MeSH terms. References from relevant publications were systematically screened.

**PARTICIPANTS/MATERIALS, SETTING, METHODS:**

Results were reported in accordance with the PRISMA-ScR guidelines. Studies that did not report original data, not written in English or providing a review of the field were excluded. From the 2182 publications retrieved, 36 papers were selected and analyzed, focusing on sample characterization (patients, controls, tissues, and fluids) and methodologies used.

**MAIN RESULTS AND THE ROLE OF CHANCE:**

Sound evidence is lacking to support a specific gut dysbiosis profile in women with endometriosis. The largest metagenome study performed using shotgun sequencing and controlling for multiple hypotheses testing did not detect significant differences between women with and without the disease. For eutopic and ectopic tissue microbiomes, the literature is too scant to draw any conclusion. Some data suggest a possible enrichment of *Streptococcus* sp. in cervical fluid and of *Pseudomonas* sp. in peritoneal fluid and a depletion of *Lachnospira* sp. in stool/anal fluid of endometriosis patients. However, these findings may be explained by confounders or by intrinsic patient or population characteristics. We appraised the limitations of the studies and proposed suggestions for optimizing sequencing techniques and experimental designs.

**LIMITATIONS, REASONS FOR CAUTION:**

The number of participants per study greatly varied and, with few exceptions, was typically low. Incomplete information on methodological approaches was broadly observed. The impact of participants’ menstrual cycle phase, diet, and drug assumption was frequently not considered.

**WIDER IMPLICATIONS OF THE FINDINGS:**

Standardization of research protocols to allow reproducibility is required, as well as collaborations to harmonize data analysis, interpretation, and, more importantly, health outcome prediction or improvement.

**STUDY FUNDING/COMPETING INTEREST(S):**

The review was funded by the Italian Ministry of Health: RF-2019-12369460, and Current Research IRCCS. P.Vi. serves as co-editor in Chief of Journal of Endometriosis and Uterine Disorders. E.S. serves as Editor in Chief of Human Reproduction Open and discloses research grants from Ferring, Ibsa, Gedeon Richter, and Theramex, and honoraria from Ibsa and Gedeon Richter. P.Ve. serves as Associate Editor for Human Reproduction Open; is a member of the Editorial Board of the Journal of Obstetrics and Gynaecology Canada, of the Italian Journal of Obstetrics and Gynaecology, and of the International Editorial Board of Acta Obstetricia et Gynecologica Scandinavica; has received royalties from Wolters Kluwer for chapters on endometriosis management in the clinical decision support resource UpToDate; and maintains both a public and private gynecological practice. All other authors declare they have no conflict of interest.

**REGISTRATION NUMBER:**

10.17605/OSF.IO/X6HBT at https://osf.io/registries.

WHAT DOES THIS MEAN FOR PATIENTS? Endometriosis is when tissue similar to the lining of the uterus grows in other parts of the body, often on the ovaries, fallopian tubes, and other pelvic organs. This can cause pelvic pain, fatigue, and fertility problems. Despite many years of research, it is still not clear exactly how and why endometriosis develops. Recently, scientists have started to explore whether the gut and reproductive system’s microbiome, their community of bacteria and other microorganisms, may play a role.

Our review looked at studies comparing the microbiome in women with and without endometriosis across different body sites. Most of these studies were small, used different methods, and did not always account for important factors like diet, stage of the menstrual cycle, or antibiotic and hormonal use. While a few studies found differences in the types of bacteria present, the results across studies were inconsistent and sometimes even contradictory. This means there is currently no clear evidence of a specific ‘endometriosis microbiome’.

For patients, this means that microbiome testing or treatments are not part of standard care for endometriosis. Importantly, antibiotics should not be used, as there is no evidence they improve endometriosis and their unnecessary use can disrupt healthy bacteria, cause side effects, and contribute to antibiotic resistance, a serious public health problem. More and better research is needed before we know if the microbiome plays a role in endometriosis.

## Introduction

Nearly a century after Sampson proposed the retrograde menstruation theory, accumulating evidence suggests that no single mechanism can fully explain the diverse clinical manifestations associated with endometrial tissue at ectopic sites. Many alternative hypotheses have been proposed to explain the development of endometriosis, yet its precise etiology remains unknown (Abbott *et al.*, 2024). Interestingly, recent findings have implicated microbiota dysfunctions, particularly bacterial infections, as potential causal factors. Thus, this research area has become particularly active. The link between endometriosis and microbiome alterations is supported by a few studies conducted on murine models of the disease (Yuan *et al.*, 2018; Chadchan *et al.*, 2023; Hu *et al.*, 2023; Muraoka *et al.*, 2023; Wei *et al.*, 2023). Among these, the most interesting breakthrough was reported by Muraoka *et al.* (2023), who showed that uterine tissue fragments infected with *Fusobacterium nucleatum*, when injected into recipient mice, led to larger endometriotic lesions characterized by increased M2 macrophage infiltration, elevated expression of transforming growth factor (TGF)-β1, and a greater number of transgelin-positive myofibroblasts. Importantly, antibiotic treatment reduced lesion weight, underscoring a potential therapeutic avenue.

Earlier murine studies linked endometriosis to alterations in the gut microbiome rather than the uterine microbiome (Yuan *et al.*, 2018; Chadchan *et al.*, 2023). Similarly, treatment with broad-spectrum antibiotics reduced the risk of endometriosis also in these models (Chadchan *et al.*, 2019). In human studies, four systematic reviews have synthesized evidence regarding gut, endometrial, and vaginal microbiome composition in patients with endometriosis (Leonardi *et al.*, 2020; D’Alterio *et al.*, 2021; Colonetti *et al.*, 2023; Weber *et al.*, 2024). These reviews, however, highlighted inconsistencies across studies, with no definitive trends indicating whether bacterial changes in patients with endometriosis were predominantly beneficial or harmful.

Significant knowledge gaps persist in this field. Understanding whether and how microbiome dysbiosis may influence the development and/or progression of endometriosis could guide or limit future interventions targeting bacterial colonization. To address these gaps, we chose to conduct a scoping review to systematically evaluate the existing literature, assess research methodologies with a particular emphasis on sampling and analytic techniques, and explore potential microbiome-related exposures in affected patients. This way, we have systematically and critically mapped the existing literature addressing the following primary questions:

1. Microbiome characterization: What data are available concerning gut and reproductive tract microbiomes in patients with endometriosis?
2. Methodological assessment: What are the methodological design methods, sequencing platform, analytical pipelines employed in microbiome studies on endometriosis?

## Methods

### Protocol and registration

This scoping review was conducted in accordance with the PRISMA-ScR guidelines (Preferred Reporting Items for Systematic Reviews and Meta-Analyses extension for Scoping Reviews) (Tricco *et al*., 2018), which provides a widely accepted framework to ensure transparency and methodological rigor in exploratory reviews. The methodological approach was based on the foundational framework originally proposed by Arksey and O’Malley (2005) and later refined by Levac *et al*. (2010) and the Joanna Briggs Institute Reviewers’ Manual (Aromataris *et al*. 2024). Specifically, the review process adhered to five key stages: identifying the research question, searching the literature, selecting relevant studies, charting the data, and synthesizing and reporting the results. These established protocols helped maintain consistency and reproducibility throughout the review process. The scoping review protocol was recorded *a priori* and is available at OSF registries (Registration doi: 10.17605/OSF.IO/X6HBT; https://osf.io/registries).

### Information sources and search

The PubMed, EMBASE, and Web of Science databases were searched to identify studies on endometriosis microbiome characterization, using a combination of MeSH terms (search string available in Supplementary File S1). The scoping review included all studies published from January 2016 to November 2024. Papers published before 2016 were excluded because sequencing technologies were in developmental stages and had not yet been widely adopted. Starting in 2016, sequencing methods, particularly next-generation sequencing, became more commonly applied, improving consistency and reproducibility in microbiome analyses due to advancements in depth, accuracy, and standardization (Wen *et al*., 2017).

### Study selection

The initial literature search was conducted on 5 February 2024, with the most recent update performed on 11 November 2024. Studies were included if they met the following criteria:

1. the study population consisted of women diagnosed with endometriosis and an appropriate control group;
2. the microbiome of the reproductive tract or gastrointestinal system was analyzed using molecular techniques; and
3. the study followed an experimental design and was published in English.

The screening process was conducted in two phases. First, titles and abstracts were assessed to exclude clearly irrelevant publications. Subsequently, full-text reviews were carried out to confirm final eligibility. Both stages were independently performed by two reviewers (G.D.S. and D.D.), and any disagreements were resolved by discussion with a third reviewer (M.C.) to ensure objectivity and consensus. The final list of included studies was then reviewed and discussed by the full research team to support the technical and methodological integrity of the selection process.

### Data-charting process

A standardized data-charting form was developed to guide the extraction of key variables. Three co-authors (D.D., G.D.S., and M.C.) independently extracted and charted data from each included study. Discrepancies were resolved through discussion and consensus. The form was iteratively refined as needed to ensure consistency and completeness in data collection.

### Data items

First, the authors collected a combination of qualitative or quantitative data from the final list of publications in a ‘s-charting form’ (Arksey and O’Malley, 2005), reporting: (i) study characteristics, including study design and sample size of study groups; (ii) sample location and (iii) sampling method; (iv) population studies characteristics; (v) inclusion and exclusion criteria; (vi) endometriosis stage/phenotype and diagnostic methods; (vii) technical characteristics of microbiome analysis; (viii) technical characteristics of bioinformatic analysis; and (ix) descriptive results, including alpha and beta diversity (a glossary of microbiome terminology is available in Supplementary Table S1).

### Bias analysis

To ensure a structured evaluation of the quality of the studies included in this review, the widely recognized Newcastle–Ottawa Scale (NOS) was employed. This tool was used exclusively to assess the quality of patient selection and group comparability, and not to evaluate methodological or bioinformatic procedures such as sequencing or data analysis. The NOS consists of an 8-item checklist that focuses on three key domains: the selection of study participants, the comparability of groups, and the ascertainment of exposure or outcomes. In accordance with established guidance (Stang, 2010), two independent reviewers applied the NOS criteria to each included study to ensure consistency and minimize subjective bias in the assessment.

## Results

### Search results

**Figure 1.**
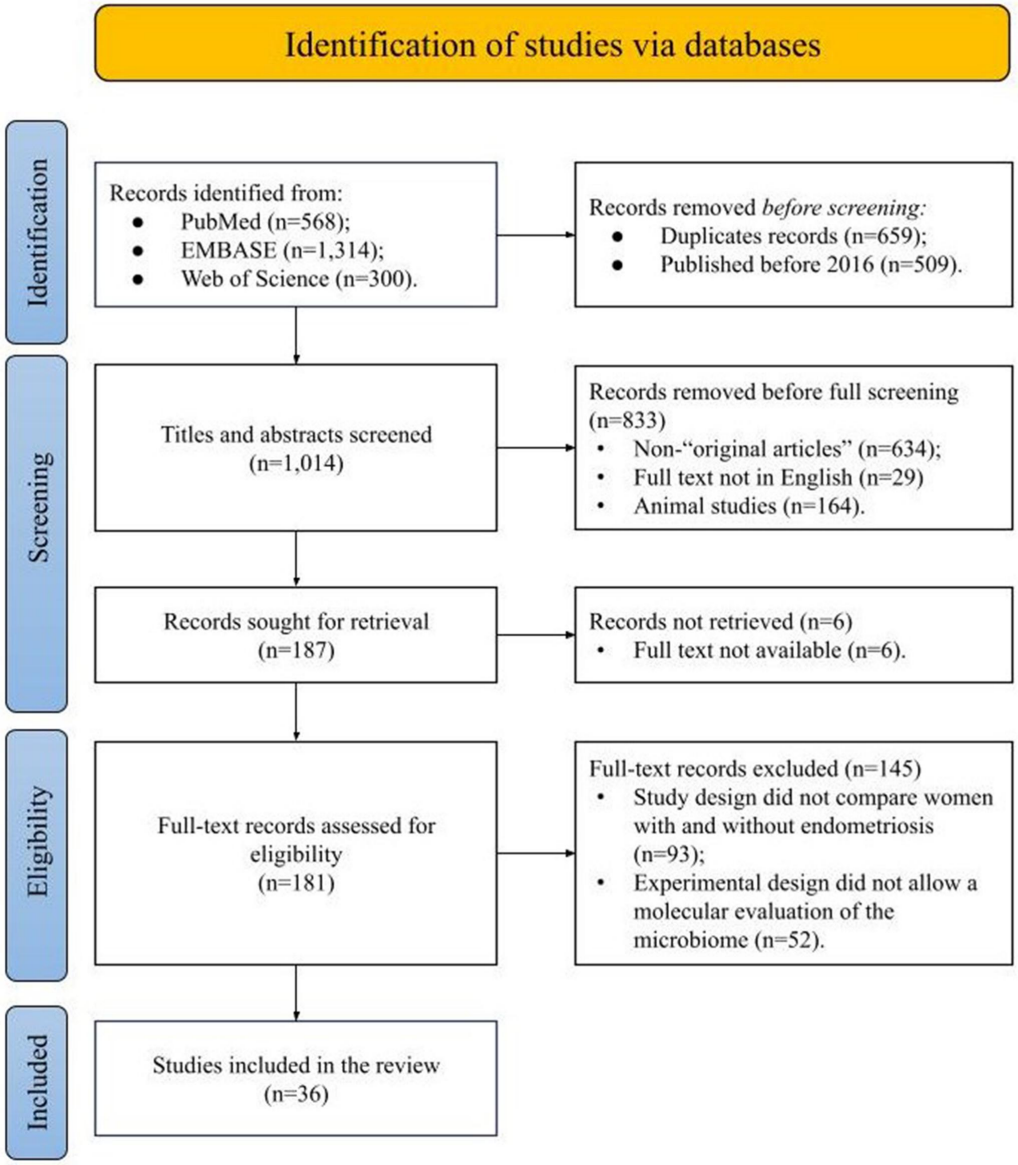
**PRISMA flow diagram illustrating the study selection process**.

The study selection process is summarized in the PRISMA flow diagram (Fig. 1). A total of 2182 studies were initially retrieved from three databases: PubMed, EMBASE, and Web of Science. After removing 659 duplicates and excluding 509 studies published before 2016, 1014 records remained for title and abstract screening. Of these, 181 studies were selected for full-text review. Following the full-text assessment, 145 studies were excluded: 93 did not include a comparison between women with and without endometriosis, and 52 did not enable a molecular-level evaluation of the microbiome. Ultimately, 36 studies met the inclusion criteria and were included in the review. A summary of these studies is provided in Table 1.

**Table 1.** Overview of the articles included in the review.

Authors (year)	Country	Samples analyzed for microbiome	Study design	**Patients included** (total)	Population	**Endometriosis phenotype incldued** (OMA/DIE/SUP)	**Stages of endometriosis included** (rASRM)	Endometriosis diagnosis	Use of hormonal treatments as exclusion criteria	**Phase of the menstrual cycle** (for those not in use of hormonal treatments)	**Use of antibiotics treatment as an exclusion criteria** (at least in the last 3 months)	Special eating habits as an exclusion criteria
Guo *et al.* (2025)	China	Eutopic endometrium.	NR	43	Symptomatic women with and without endometriosis	NR	NR (median rASRM score: 45.48 ± 19.27)	Surgery followed by histological confirmation	Yes	NR	Yes	No
Malvezzi *et al.* (2025)	Brazil	Peritoneal fluid.	Pilot case-control study	50	Women undergoing laparoscopy surgery for endometriotic lesions excision and for other benign gynecological conditions or tubal ligation	SUP, OMA, DIE	NR	Surgery followed by histological confirmation	No, but data NR.	Follicular or luteal. (*P*=NS)	No, but data NR.	No
Guo *et al.* (2024)	China	Stool.	NR	36	Women with endometriosis and healthy women.	NR	NR	Clinical, Doppler ultrasound, or surgery.	No, but data NR.	NR	Yes	No
Do *et al.* (2024)	USA	Stool; Vaginal fluid.	NR	48	Women undergoing laparoscopy surgery for endometriosis and other benign gynecological conditions	NR	I–IV	Surgery	No, but its use was not statistically different between groups.	NR	Yes	No
MacSharry *et al.* (2024)	Ireland	Vaginal fluid.	NR	40	Women undergoing laparoscopy surgery for the investigation of infertility, pelvic pain, or pelvic pathology suspected on ultrasound scan.	OMA, SUP	I–IV	Surgery	Yes	Diverse phases (*P*=NS)	No, and the use was in different rates between groups (*P*=NR)	No
Hicks *et al.* (2025)	Australia	Stool; Vaginal fluid; Oropharyngeal fluid.	Prospective cohort pilot study	64	Women undergoing laparoscopy surgery to exclude or diagnose endometriosis and healthy women with no known gynecological symptoms or infertility concerns.	NR	I–IV	Surgery followed by histological confirmation	No, but data NR.	NR	NR	No
Sessa *et al.* (2024)	Italy	Vaginal fluid.	Cross-sectional study	123	Women undergoing a gynecological examination followed by a transvaginal ultrasound to exclude or diagnose endometriosis.	OMA, DIE	NR	Transvaginal ultrasound.	No, data reported only in the endometriosis group (41.6%).	At the time of ovulation	Yes	No
Pérez-Prieto *et al.* (2024)	Spain	Stool.	Case-control study	1000	Estonian women with and without endometriosis	NR	NR	Surgery	No, but its use was not statistically different between groups.	NR	No, but the frequency of antibiotics consumption in the last year was not statistically different between groups. (*P*=NS)	No
Jimenez *et al.* (2024)	USA	Anal fluid; Vaginal fluid.	Pilot study	73	Women with chronic pelvic pain with or without endometriosis and women with other benign gynecological disorders	NR	I–IV	Surgery followed by histological confirmation	No, but its use was not statistically different between groups.	NR	Yes	No
Marcos *et al.* (2024)	Spain	Stool; Vaginal fluid; Uterine fluid; Eutopic endometrium; Oropharyngeal fluid.	Cohort study	21	Women with fertility problems undergoing IVF with and without endometriosis	NR	NR	Imaging or Surgery	No (*P*=NR)	At the time of ovulation.	Yes	No
Zhu *et al.* (2024)	China	Peritoneal fluid; Endometrial fluid.	NR	57	Infertile women undergoing laparoscopy due to endometriosis or tubal obstruction.	NR	I–IV	Surgery followed by histological confirmation	Yes	NR	Yes	No
Chen *et al.* (2024)	China	Endometriotic tissue.	NR	45	Women undergoing laparoscopy due to suspected endometriosis or due to uterine fibroids.	OMA	III–IV	Surgery followed by histological confirmation	Yes	Mainly follicular (>90%) (*P*=NR)	No, but data NR.	No
Pai *et al.* (2023)	Taiwan	Stool.	Case-control study	51	Women undergoing surgeries for benign gynecological diseases.	NR	I–IV	Surgery followed by histological confirmation	No (*P*=NR)	NR	Yes	Yes
Hu *et al.* (2023)	China	Endometriotic tissue; Stool.	NR	38	Symptomatic women with moderate to severe endometriosis (rASRM stage III/IV) and women undergoing laparoscopic surgery for tubal examination without endometriosis.	OMA	III/IV	Surgery followed by histological confirmation	Yes	Early follicular.	Yes	No
Yang *et al.* (2023)	China	Cervical fluid; Vaginal fluid.	NR	40	Women with ovarian endometriosis undergoing laparoscopy surgery and healthy women undergoing routine physical examination.	OMA	I–IV	Surgery followed by histological confirmation	Yes	Follicular.	Yes *(in the last 30 days)*	No
Muraoka *et al.* (2023)	Japan	Vaginal fluid; Eutopic endometrium; Endometriotic tissue.	NR	155	Women undergoing laparoscopy for endometriosis or for other gynecological disorders.	OMA	NR	Surgery	Yes	Follicular or luteal. (*P*=NR)	Yes *(in the last 30 days)*	No
Wei *et al.* (2023)	China	Stool.	NR	65	Women with endometriosis and infertile or healthy women who had undergone a gynecological or radiological examination.	DIE	I-IV	Surgery followed by histological confirmation	Yes	Early follicular.	Yes	Yes
Lu *et al.* (2022)	China	Vaginal fluid.	NR	34	Women with endometriosis and healthy women.	NR	I-IV	Surgery followed by histological confirmation	Yes	NR	Yes *(in the last 30 days)*	No
Chang *et al.* (2022)	Taiwan	Cervical fluid.	NR	33	Women undergoing laparotomy or laparoscopy due to endometriosis and healthy women receiving regular physiological checks.	NR	I-IV	Surgery followed by histological confirmation	NR	NR	NR	No
Yuan *et al.* (2022)	China	Peritoneal fluid.	NR	61	Women undergoing laparoscopy due to endometriosis or for other benign gynecologic diseases.	OMA	I-IV	Surgery followed by histological confirmation	Yes	Follicular or luteal. (*P*=NS)	Yes	No
Huang *et al.* (2021)	China	Stool; Cervical fluid; Peritoneal fluid.	NR	41	Women undergoing a laparoscopy due to endometriosis or other gynecological diseases.	NR	I-IV	Surgery followed by histological confirmation	Yes	Follicular or luteal. (*P*=NR)	Yes	No
Le *et al.* (2021)	USA	Anal fluid; Vaginal fluid.	NR	29	Women undergoing laparoscopy/laparotomy for the investigation of unexplained pelvic pain, suspicion of or known to have endometriosis or for either benign uterine or ovarian indications.	NR	I-IV	Surgery	No, but its use was not statistically different between groups.	NR	NR	No
Wessels *et al.* (2021)	Canada	Eutopic endometrium.	Prospective cross-sectional study	21	Women undergoing gynecological laparoscopy for pelvic pain thought to be due to endometriosis.	NR	I-IV	Surgery followed by histological confirmation	Yes	Follicular, luteal, or menstrual. (*P*=NS)	NR	No
Khan *et al.* (2021)	Japan	Eutopic endometrium.	Prospective non-randomized observational study	100	Women undergoing laparoscopy for infertility, dysmenorrhea, or fertile women undergoing laparoscopy for uterine fibroids.	OMA	I-IV	Surgery followed by histological confirmation	No, the use of hormonal treatments was part of the study’s methodology.	Diverse phases in similar rates between groups (*P*=NR)	No, the use of antibiotic treatments was part of the study’s methodology.	No
Chao *et al.* (2021)	China	Vaginal fluid.	Cross-sectional study	128	Women with CPPS undergoing surgery with and without endometriosis and healthy women visiting for routine examinations or health counselling.	NR	NR	Surgery alone or surgery followed by histological confirmation	No, but its use was not statistically different between groups.	Diverse phases (*P*=NS)	Yes	No
Lee *et al.* (2021)	South Korea	Peritoneal fluid’s EVs.	NR	90	Women undergoing laparoscopy surgery due to endometriosis, uterine leiomyoma, or benign ovarian cyst.	OMA	III/IV	Surgery followed by histological confirmation	Yes	Follicular.	Yes	No
Shan *et al.* (2021)	China	Stool.	NR	24	Women with endometriosis and healthy women.	NR	III-IV	Surgery followed by histological confirmation	Yes	Early follicular.	Yes	Guided carbohydrate-based diet for 3 days.
Svensson *et al.* (2021)	Sweden	Stool.	NR	264	Healthy women previously recruited for other studies and women with endometriosis.	OMA, SUP, and DIE	NR	Surgery	No, and the use of hormonal treatments was significantly higher in the endometriosis groups. *P* < 0.001	NR	No, but its use was not statistically different between groups. (*P*=NS)	No
Wei *et al.* (2020)	China	Vaginal fluid; Cervical mucus; Peritoneal fluid; Endometrial fluid.	NR	50	Women undergoing laparoscopic surgery due to pelvic endometriosis or benign gynecological diseases.	NR	I-IV	Surgery followed by histological confirmation	Yes	Early follicular.	Yes	No
Hernandes *et al.* (2020)	Brazil	Vaginal fluid; Eutopic endometrium; Endometriotic tissue.	Case-control study	21	Women undergoing laparoscopy due to suspected or confirmed deep endometriosis or for other gynecologic indications.	DIE	NR	Surgery followed by histological confirmation	No, but data NR.	NR	Yes	No
Perrotta *et al.* (2020)	Brazil	Anal fluid; Vaginal fluid.	Observational cross-sectional study	59	Women undergoing laparoscopy due to suspected or confirmed endometriosis or for other gynecologic indications.	OMA, SUP, and DIE	I-IV	Imaging or Surgery	Yes	Menstrual and follicular.	Yes	No
Akiyama *et al.* (2019)	Japan	Cervical mucus.	NR	69	Women undergoing laparoscopy surgery due to endometriosis, fibroids, or benign ovarian tumor.	NR	III-IV	Surgery followed by histological confirmation	Yes	Follicular or luteal, in the same proportion between groups. (*P*=NS)	Yes	No
Ata *et al.* (2019)	Turkey	Stool; Cervical fluid; Vaginal fluid.	Prospective observational cohort study.	28	Caucasian women with a histologic diagnosis of endometriosis and healthy women who presented for a routine visit or preconceptional counselling.	OMA, SUP, and DIE	III-IV	Surgery followed by histological confirmation	Yes	Follicular or luteal. (*P*=NS)	Yes	No
Wang *et al.* (2018)	China	Peritoneal fluid.	NR	85	Infertile women undergoing laparoscopic surgery due to endometriosis and women without endometriosis.	NR	I-IV	Surgery	Yes	Early follicular.	NR	No
Campos *et al.* (2018)	Brazil	Cervical fluid; Peritoneal fluid; Endometriotic tissue; Peritoneal tissue.	NR	104	Symptomatic (CPP, severe or incapacitating dysmenorrhea, deep dyspareunia, cyclic urinary or bowel abnormalities, and infertility) women submitted to video laparoscopy with and without endometriosis.	OMA, SUP, and DIE	I-IV	Surgery followed by histological confirmation	No, the use of hormonal treatments was higher in the endometriosis group (*P*=NR)	Follicular or luteal. (*P*=NS)	Yes	No
Khan *et al.* (2016)	Japan	Endometrial fluid (mixed with cells); Ovarian cyst fluid.	NR	64	Women undergoing laparoscopy for endometriosis, or for dermoid cyst/serous cyst adenoma/mucinous cyst adenoma or uterine myoma.	NR	I-IV	Surgery followed by histological confirmation	The use of hormonal treatments was part of the study methodology.	Diverse phases in different rates between groups (*P*=NR)	NR	No

CCP, chronic pelvic pain; DIE, deep infiltrating endometriosis; Evs, extracellular vesicles; NR, not reported; ns, non-significant; OMA, ovarian endometriosis; rASRM, revised American Society of Reproductive Medicine classification; SUP, superficial endometriosis.

### Characteristics of included studies

Of the 36 studies included in this review (Table 1), the types of biological samples analyzed were distributed as follows:

- stool/anal fluid: 15 studies (41.7%) (Ata *et al.*, 2019; Perrotta *et al.*, 2020; Huang *et al.*, 2021; Le *et al.*, 2021; Shan *et al.*, 2021; Svensson *et al.*, 2021; Hu *et al.*, 2023; Pai *et al.*, 2023; Wei *et al.*, 2023; Do *et al.*, 2024; Guo *et al.*, 2024; Jimenez *et al.*, 2024; Marcos *et al.*, 2024; Pérez-Prieto *et al.*, 2024; Hicks *et al.*, 2025);
- vaginal fluid: 15 studies (41.7%) (Ata *et al.*, 2019; Hernandes *et al.*, 2020; Perrotta *et al.*, 2020; Wei *et al.*, 2020; Chao *et al.*, 2021; Le *et al.*, 2021; Lu *et al.*, 2022; Muraoka *et al.*, 2023; Yang *et al.*, 2023; Do *et al.*, 2024; Jimenez *et al.*, 2024; MacSharry *et al.*, 2024; Marcos *et al.*, 2024; Sessa *et al.*, 2024; Hicks *et al.*, 2025);
- cervical mucus/fluid: seven studies (19.4%) (Campos *et al.*, 2018; Akiyama *et al.*, 2019; Ata *et al.*, 2019; Wei *et al.*, 2020; Huang *et al.*, 2021; Chang *et al.*, 2022; Yang *et al.*, 2023);
- peritoneal fluid: eight studies (22.2%) (Campos *et al.*, 2018; Wang *et al.*, 2018; Wei *et al.*, 2020; Huang *et al.*, 2021; Lee *et al.*, 2021; Yuan *et al.*, 2022; Zhu *et al.*, 2024; Malvezzi *et al.*, 2025);
- uterine/endometrial fluid: four studies (11.1%) (Khan *et al.*, 2016; Wei *et al.*, 2020; Marcos *et al.*, 2024; Zhu *et al.*, 2024);
- ovarian cyst fluid: one study (2.7%) (Khan *et al.*, 2016);
- oropharyngeal fluid: two studies (5.6%) (Marcos *et al.*, 2024; Hicks *et al.*, 2025);
- eutopic endometrium: seven studies (19.4%) (Hernandes *et al.*, 2020; Khan *et al.*, 2021; Wessels *et al.*, 2021; Muraoka *et al.*, 2023; Marcos *et al.*, 2024; Guo *et al.*, 2025);
- endometriotic tissue: five studies (13.9%) (Campos *et al.*, 2018; Hernandes *et al.*, 2020; Hu *et al.*, 2023; Muraoka *et al.*, 2023; Chen *et al.*, 2024);

The majority of studies were conducted in East Asian populations: 14 in China (Wang *et al.*, 2018; Wei *et al.*, 2020, 2023; Chao *et al.*, 2021; Huang *et al.*, 2021; Shan *et al.*, 2021; Lu *et al.*, 2022; Yuan *et al.*, 2022; Hu *et al.*, 2023; Yang *et al.*, 2023; Chen *et al.*, 2024; Guo *et al.*, 2024, 2025; Zhu *et al.*, 2024), four in Japan (Khan *et al.*, 2016, 2021; Akiyama *et al.*, 2019; Muraoka *et al.*, 2023), two in Taiwan (Chang *et al.*, 2022; Pai *et al.*, 2023), and one in South Korea (Lee *et al.*, 2021). European-based studies included one in Ireland (MacSharry *et al.*, 2024), one in Italy (Sessa *et al.*, 2024), one in Sweden (Svensson *et al.*, 2021), one in Turkey (Ata *et al.*, 2019), and two in Spain, one involving a Spanish population (Marcos *et al.*, 2024) and the other an Estonian population (Pérez-Prieto *et al.*, 2024). Eight studies were from the Americas: four from Brazil (Campos *et al.*, 2018; Hernandes *et al.*, 2020; Perrotta *et al.*, 2020; Malvezzi *et al.*, 2025), three from the USA (Le *et al.*, 2021; Do *et al.*, 2024; Jimenez *et al.*, 2024), and one from Canada (Wessels *et al.*, 2021). Only one was conducted in Australia (Hicks *et al.*, 2025; Table 1).

This review included only original studies that evaluated the gut or reproductive tract microbiome in women with endometriosis (cases) compared to women without the condition (controls). However, only a small number of included studies explicitly reported using a case-control design (Hernandes *et al.*, 2020; Pai *et al.*, 2023; Pérez-Prieto *et al.*, 2024; Malvezzi *et al.*, 2025). Four were reported as cross-sectional studies (Perrotta *et al.*, 2020; Chao *et al.*, 2021; Wessels *et al.*, 2021; Sessa *et al.*, 2024), and three as cohort studies (Ata *et al.*, 2019; Marcos *et al.*, 2024; Hicks *et al.*, 2025). Most studies, however, did not clearly report their study design (Khan *et al.*, 2016, 2021; Campos *et al.*, 2018; Wang *et al.*, 2018; Akiyama *et al.*, 2019; Wei *et al.*, 2020, 2023; Huang *et al.*, 2021; Le *et al.*, 2021; Lee *et al.*, 2021; Shan *et al.*, 2021; Svensson *et al.*, 2021; Chang *et al.*, 2022; Lu *et al.*, 2022; Yuan *et al.*, 2022; Hu *et al.*, 2023; Muraoka *et al.*, 2023; Yang *et al.*, 2023; Chen *et al.*, 2024; Do *et al.*, 2024; Guo *et al.*, 2024, 2025; Jimenez *et al.*, 2024; MacSharry *et al.*, 2024; Zhu *et al.*, 2024).

Sample sizes varied significantly across studies, ranging from as few as 21 participants (Hernandes *et al.*, 2020; Wessels *et al.*, 2021; Marcos *et al.*, 2024) to up to 1000 participants (Pérez-Prieto *et al.*, 2024). Notably, 30 out of 36 studies included 100 participants or fewer.

The composition of control groups was also highly heterogeneous. Only 10 studies (Ata *et al.*, 2019; Chao *et al.*, 2021; Shan *et al.*, 2021; Svensson *et al.*, 2021; Chang *et al.*, 2022; Lu *et al.*, 2022; Wei *et al.*, 2023; Yang *et al.*, 2023; Guo *et al.*, 2024; Hicks *et al.*, 2025) included at least a subgroup of healthy women as controls. In contrast, the remaining studies used control groups composed of women with other gynecological conditions unrelated to endometriosis, such as infertility, uterine fibroids, tubal obstruction, leiomyomas, ovarian cysts, and chronic pelvic pain.

Regarding the type of endometriosis evaluated, five studies did not specify either the disease phenotype or the stages (Chao *et al.*, 2021; Guo *et al.*, 2024, 2025; Marcos *et al.*, 2024; Pérez-Prieto *et al.*, 2024). In contrast, only 11 studies provided complete information, reporting both the phenotype and stage of endometriosis (Campos *et al.*, 2018; Ata *et al.*, 2019; Perrotta *et al.*, 2020; Khan *et al.*, 2021; Lee *et al.*, 2021; Yuan *et al.*, 2022; Hu *et al.*, 2023; Wei *et al.*, 2023; Yang *et al.*, 2023; Chen *et al.*, 2024; MacSharry *et al.*, 2024). The methods used to diagnose endometriosis varied across the included studies. Most studies included only participants diagnosed through surgery followed by histological confirmation (Khan *et al.*, 2016, 2021; Campos *et al.*, 2018; Akiyama *et al.*, 2019; Ata *et al.*, 2019; Hernandes *et al.*, 2020; Wei *et al.*, 2020, 2023; Huang *et al.*, 2021; Lee *et al.*, 2021; Shan *et al.*, 2021; Wessels *et al.*, 2021; Chang *et al.*, 2022; Lu *et al.*, 2022; Yuan *et al.*, 2022; Hu *et al.*, 2023; Pai *et al.*, 2023; Yang *et al.*, 2023; Chen *et al.*, 2024; Jimenez *et al.*, 2024; Zhu *et al.*, 2024; Guo *et al.*, 2025; Hicks *et al.*, 2025; Malvezzi *et al.*, 2025).

Several important confounders relevant to microbiome research were considered during this review, including the use of hormonal treatments, antibiotic usage, special dietary habits, and the menstrual phase at the time of sample collection (Table 1). Notably, eight studies (Campos *et al.*, 2018; Hernandes *et al.*, 2020; Chang *et al.*, 2022; Pai *et al.*, 2023; Guo *et al.*, 2024; Marcos *et al.*, 2024; Hicks *et al.*, 2025; Malvezzi *et al.*, 2025) did not report whether hormonal treatment was used as an exclusion criterion or whether its use differed between groups. Similarly, nine studies either failed to report antibiotic use (Khan *et al.*, 2016; Wang *et al.*, 2018; Le *et al.*, 2021; Wessels *et al.*, 2021; Chang *et al.*, 2022) or did not clarify whether usage differed across groups (Chen *et al.*, 2024; MacSharry *et al.*, 2024; Hicks *et al.*, 2025; Malvezzi *et al.*, 2025).

Only three studies addressed dietary factors (Table 1): two excluded participants with specific eating habits (Pai *et al.*, 2023; Wei *et al.*, 2023), while one prescribed a standardized diet prior to sampling (Shan *et al.*, 2021). In addition, several studies (Hernandes *et al.*, 2020; Le *et al.*, 2021; Svensson *et al.*, 2021; Chang *et al.*, 2022; Lu *et al.*, 2022; Pai *et al.*, 2023; Do *et al.*, 2024; Guo *et al.*, 2024, 2025; Jimenez *et al.*, 2024; Pérez-Prieto *et al.*, 2024; Zhu *et al.*, 2024; Hicks *et al.*, 2025) did not report the menstrual cycle phase during sample collection, or failed to clarify whether distribution across phases differed between groups (Khan *et al.*, 2016, 2021; Huang *et al.*, 2021; Muraoka *et al.*, 2023; Chen *et al.*, 2024). Specific differences among study groups will be addressed in the results for each sample type.

### Microbiome detection in stool or anal fluid

Technical characteristics of the 15 studies (Ata *et al.*, 2019; Perrotta *et al.*, 2020; Huang *et al.*, 2021; Le *et al.*, 2021; Shan *et al.*, 2021; Svensson *et al.*, 2021; Hu *et al.*, 2023; Pai *et al.*, 2023; Wei *et al.*, 2023; Do *et al.*, 2024; Guo *et al.*, 2024; Jimenez *et al.*, 2024; Marcos *et al.*, 2024; Pérez-Prieto *et al.*, 2024; Hicks *et al.*, 2025) providing comprehensive taxonomic evaluations of stool microbiome are detailed in Table 2.

**Table 2.** Technical characteristics of microbiome analyses across stool/anal fluid samples in the included studies.

Authors (year)	**Study groups** (cases vs controls)	**Number of samples included in the analysis** (cases vs controls)	**Age (years)** (cases vs controls)	**BMI (kg/m^2^)** (cases vs controls)	Sampling technique	**Extraction kit—brand** *(its indication)*	Method	Sequencing platform	Sequencing instrument	Region amplified	Reads length
Guo *et al.* (2024)	Women with endometriosis vs healthy controls	18 vs 18	32.23 ± 5.25 vs 31.40 ± 3.75 ^§^ (*P*=NR)	22.53 ± 3.15 vs 21.14 ± 1.94 ^§^ (*P*=NR)	‘*Collect the first morning feces*’	TIANamp Stool DNA Kit—TianGen *(For extraction of high-quality genomic DNA from various stool samples)*	16S	Illumina	NovaSeq	V3–V4	250 bp paired-end
Do *et al.* (2024)	Women with endometriosis vs women without endometriosis	33 vs 15	31.6 ± 0.8 vs 33.3 ± 1.8 ^§^ (*P*=NS)	28.3 ± 1.3 vs 29.5 ± 1.9 ^§^ (*P*=NS)	‘*Fecal swabs […] samples were collected using aseptic techniques in the operating room on the day of surgery (DOS) and in clinic 1–3 weeks postsurgical intervention (PSI).*’	DNeasy PowerSoil Pro Kit—Qiagen *(For the isolation of microbial genomic DNA from all soil types)*	16S	Illumina	NR	V4	NR
Hicks *et al.* (2025)	Women with endometriosis vs (women with other gynecological diseases vs healthy controls)	21 vs (24 vs 19)	35.9 ± 8.1 vs (35.8 ± 7.3 vs 31.5 ± 3.5) ^§^ (*P*=NS)	NR	‘*Stool samples were collected using a sterile ColOff stool collection device and Stool Collection Tubes with DNA Stabiliser (Invitek Molecular GmbH, Germany)*’	PSP^®^ Spin Stool DNA Basic Kit—Invitek *(For isolation of bacterial DNA and host DNA from stool samples)*	16S	Illumina	MiSeq	V3–V4	300 bp paired-end
Pérez-Prieto *et al.* (2024)	Women with endometriosis vs women without endometriosis	136 vs 864	50.0 [40.8–57.9] vs 45.0 [36.0–54.0] ^†^ (*P* = 0.005)	25.1 [22.2–29.5] vs 24.2 [21.6; 28.6] ^†^ (*P*=NS)	‘*Fresh stool samples were collected immediately after defecation with a sterile Pasteur pipette*’	QIAamp Fast DNA Stool Mini Kit—Qiagen *(For isolation of gDNA from stool samples)*	WGS	Illumina	NovaSeq6000	Shotgun sequencing	250 bp paired-end
Jimenez *et al.* (2024)	Women with chronic pelvic pain with endometriosis vs women with other gynecological disorders without chronic pelvic pain	35 vs 15	34.7 ± 8.8 vs 40.6 ± 8.2 ^§^ (*P*=NS)	Reported by three ranges of age (*P*=NS).	‘*Enrolled patients underwent sample collection in the operating room after administration of anesthesia but before the pelvic exam, lubricant use, or vaginal prep administration. […], and sterile swabs were used to collect samples from […] the rectum.*’	DNaeasy PowerSoil Pro Kit—Qiagen *(For the isolation of microbial genomic DNA from all soil types)*	16S	Illumina	MiSeq	V4	NR
Marcos *et al.* (2024)	Infertile women with endometriosis vs women with other infertility-related conditions	8 vs 13	42.7 ± 5.5 vs 39.4 ± 3.7 ^§^ (*P*=NS)	NR	‘*[…] intestinal sample was taken for the rectal area with the help of a sterile swab by inserting the swab at 10 cm and rotating for 30 s*’	QIAamp Fast DNA Tissue Kit—Qiagen *(For rapid isolation of genomic DNA from solid tissue samples)*	16S	Ion Torrent	PGM	NR	Single-end
Pai *et al.* (2023)	Women with endometriosis vs women without endometriosis	27 vs 24	38.1 ± 1.0 vs 37.7 ± 1.3 ^§^ (*P*=NS)	24.04 ± 0.87 vs 21.77 ± 0.73 ^§^ (*P* = 0.051)	‘*Fresh fecal samples were collected in 150 ml sterile Falcon tubes.*’	QIAamp PowerFecal Pro DNA Kits—Qiagen *(For the isolation of microbial DNA from stool and gut samples)*	16S	Illumina	MiSeq	V3–V4	300 bp
Hu *et al.* (2023)	Women with endometriotic cysts vs healthy controls	14 vs 24	30.6 [29.3–32.0] vs 29.4 [28.4–30.4] ^†^ (*P*=NS)	20.29 [19.12–21.46] vs 22.75 [21.23–24.27] ^†^ (*P* = 0.01)	‘*Fecal samples, the central part of the stool (3–5 g), was collected in a sterile tube.*’	Cetyltrimethylammonium bromide (CTAB) *(Generic method for isolating genomic DNA from different tissues)*	16S	Illumina	NovaSeq	V3–V4	250 bp paired-end
Wei *et al.* (2023)	Infertile women with endometriosis vs infertile and healthy women without endometriosis	35 vs (8 and 22)	32.6 ± 5.7 vs 30.2 ± 5.6 ^§^ (*P*=NS)	20.52 ± 2.02 vs 19.78 ± 1.55 ^§^ (*P*=NS)	‘*2 ml of fresh stool sample was collected in a sterile Falcon tube.*’	E.Z.N.A.^®^ Soil DNA Kit—Omega Bio-Tek *(For isolation of DNA from soil samples)*	16S	Illumina	MiSeq	V3–V4	300 bp paired-end
Huang *et al.* (2021)	Women with endometriosis vs healthy controls	21 vs 20	38.3 ± 7.88 vs 34.0 ± 10.8 ^§^ (*P*=NS)	21.5 ± 2.79 vs 24.3 ± 8.16 ^§^ (*P*=NS)	‘*Stool were collected using a fecal sample collection kit.*’	Quick-RNA Fecal/Soil Microbe Microprep Kit—Zymo Research *(For extract of RNA from various soil, fecal, and water samples)*	16S	Ion Torrent	S5	V4	NR
Le *et al.* (2021)	Women with endometriosis vs women without endometriosis	20 vs 9	32.5 ± 1.1 vs 32.6 ± 2.0 ^§^ (*P*=NS)	26.5 ± 1.5 vs 28.1 ± 2.4 ^§^ (*P*=NS)	‘*Sample collection methods were performed by trained medical professionals […] per the Human Microbiome Project protocol.*’	PowerMag Soil DNA Isolation Kit—MoBio *(For isolation of microbial DNA from all types of soil)*	16S	Illumina	MiSeq	V4	250 bp paired-end
Shan *et al.* (2021)	Women with endometriosis vs healthy controls	12 vs 12	32 ± 2 vs 32 ± 3 ^§^ (*P*=NS)	NR	‘*Central part of fresh feces […] were collected in sterile disposable plastic cups.*’	E.Z.N.A.^®^ Soil DNA Kit—Omega Bio-Tek *(For isolation of DNA from soil samples)*	16S	Illumina	MiSeq	V3–V4	300 bp
Svensson *et al.* (2021)	Women with endometriosis vs healthy controls	66 vs 198	37.8 [32.8–43.3] vs 37.0 [32.0–44.0] ^†^ (*P*=NS)	37.8 [32.8–43.3] vs 24.7 [22.1–27.5] ^†^ (*P*=NS)	‘*Stool samples were collected […] at home in sterile tubes and put in the freezer until they were brought to the lab.*’	QIAamp Fast DNA Stool Mini Kit—Qiagen *(For isolation of gDNA from stool samples)*	16S	Illumina	HiSeq	V1–V3	300 bp paired-end
Perrotta *et al.* (2020)	Women with endometriosis vs women without endometriosis	35 vs 24	34.9 ± 6.8 vs 35.25 ± 6.9 ^§^ (*P*=NS)	24.8 ± 4.5 vs 24.3 ± 2.7 ^§^ (*P*=NS)	‘*Rectal samples were collected by swabbing the rectal tissue at a depth of 3 cm.*’	PowerMag Soil DNA Isolation Kit—MoBio *(For isolation of microbial DNA from all types of soil)*	16S	Illumina	MiSeq	V4	250 bp paired-end
Ata *et al.* (2019)	Women with endometriosis vs healthy controls	14 vs 14	28.5 [26.0–31.3] vs 27.5 [25.8–30.0] ^†^ (*P*=NS)	23.0 [21.0–24.3] vs 21.0 [20.1–24.2] ^†^ (*P*=NS)	‘*A minimum of 5 ml fresh stool sample was collected in a 15 ml Falcon tube.*’	QIAamp Fast DNA Stool Mini Kit—Qiagen *(For isolation of gDNA from stool samples)*	16S	Illumina	MiSeq	V3–V4	300 bp paired-end

Data reported as reported by the original papers, unless otherwise stated.

Bp, base pairs; NR, not reported; NS, non-significant; WGS, whole-genome sequencing.

§ Data are expressed as mean±SD.

† Data are expressed as median [25th–75th percentile].

Sample sizes ranged from n = 8 to n = 136 for cases and n = 9 to n = 864 for controls. Only six studies (Ata *et al.*, 2019; Shan *et al.*, 2021; Svensson *et al.*, 2021; Hu *et al.*, 2023; Wei *et al.*, 2023; Hicks *et al.*, 2025) recruited healthy women as controls. Most studies reported comparable age between groups; however, the largest one (Pérez-Prieto *et al.*, 2024) showed a significant age difference (*P* = 0.005), and one study (Guo *et al.*, 2025) did not report this information. BMI was significantly different in Hu *et al.* (2023; *P* = 0.01) and was unreported in four studies (Shan *et al.*, 2021; Guo *et al.*, 2024; Marcos *et al.*, 2024; Hicks *et al.*, 2025). All others reported BMI comparability between groups.

Endometriosis diagnosis was surgical in nearly all studies, except for three that also included diagnoses based on imaging (Perrotta *et al.*, 2020; Guo *et al.*, 2024; Marcos *et al.*, 2024), generally indicating a focus on moderate to severe forms. Only six studies excluded participants on hormonal therapy (Ata *et al.*, 2019; Perrotta *et al.*, 2020; Huang *et al.*, 2021; Shan *et al.*, 2021; Hu *et al.*, 2023; Wei *et al.*, 2023) or on antibiotics therapy (Ata *et al.*, 2019; Perrotta *et al.*, 2020; Huang *et al.*, 2021; Shan *et al.*, 2021; Hu *et al.*, 2023; Wei *et al.*, 2023). Menstrual cycle phase was unreported in eight studies (Le *et al.*, 2021; Svensson *et al.*, 2021; Pai *et al.*, 2023; Do *et al.*, 2024; Guo *et al.*, 2024; Jimenez *et al.*, 2024; Pérez-Prieto *et al.*, 2024; Hicks *et al.*, 2025) and standardized to early follicular only in four (Perrotta *et al.*, 2020; Shan *et al.*, 2021; Hu *et al.*, 2023; Wei *et al.*, 2023). One study (Huang *et al.*, 2021) reported that the collection phase varied but whether there was a statistical difference between groups was not reported. Despite the known influence of diet on gut microbiota (Flint *et al.*, 2015), only two studies (Pai *et al.*, 2023; Wei *et al.*, 2023) excluded participants with specific dietary habits, and only one (Shan *et al.*, 2021) requested participants to follow a specific diet for 3 days before sample collection (Table 1).

Microbiome analysis methods are summarized in Table 2. Notably, several studies used DNA extraction kits designed for soil (Perrotta *et al.*, 2020; Le *et al.*, 2021; Shan *et al.*, 2021; Wei *et al.*, 2023; Do *et al.*, 2024; Jimenez *et al.*, 2024) or for tissue samples (Hu *et al.*, 2023; Marcos *et al.*, 2024) rather than stool samples, potentially impacting microbial yield and composition.

Nearly all studies employed 16S rRNA sequencing on Illumina or Ion Torrent platforms, though the hypervariable regions targeted varied across studies. In contrast, Pérez-Prieto *et al.* (2024) employed shotgun metagenomic paired-end sequencing, allowing for broader and deeper taxonomic resolution through whole-genome profiling. Bioinformatics pipelines, including sequence filtering, chimera removal, operational taxonomic unit (OTU) clustering, and taxonomic assignment, differed widely across studies. A complete overview of the specific pipelines used is provided in Supplementary Table S2.

**Figure 2.**
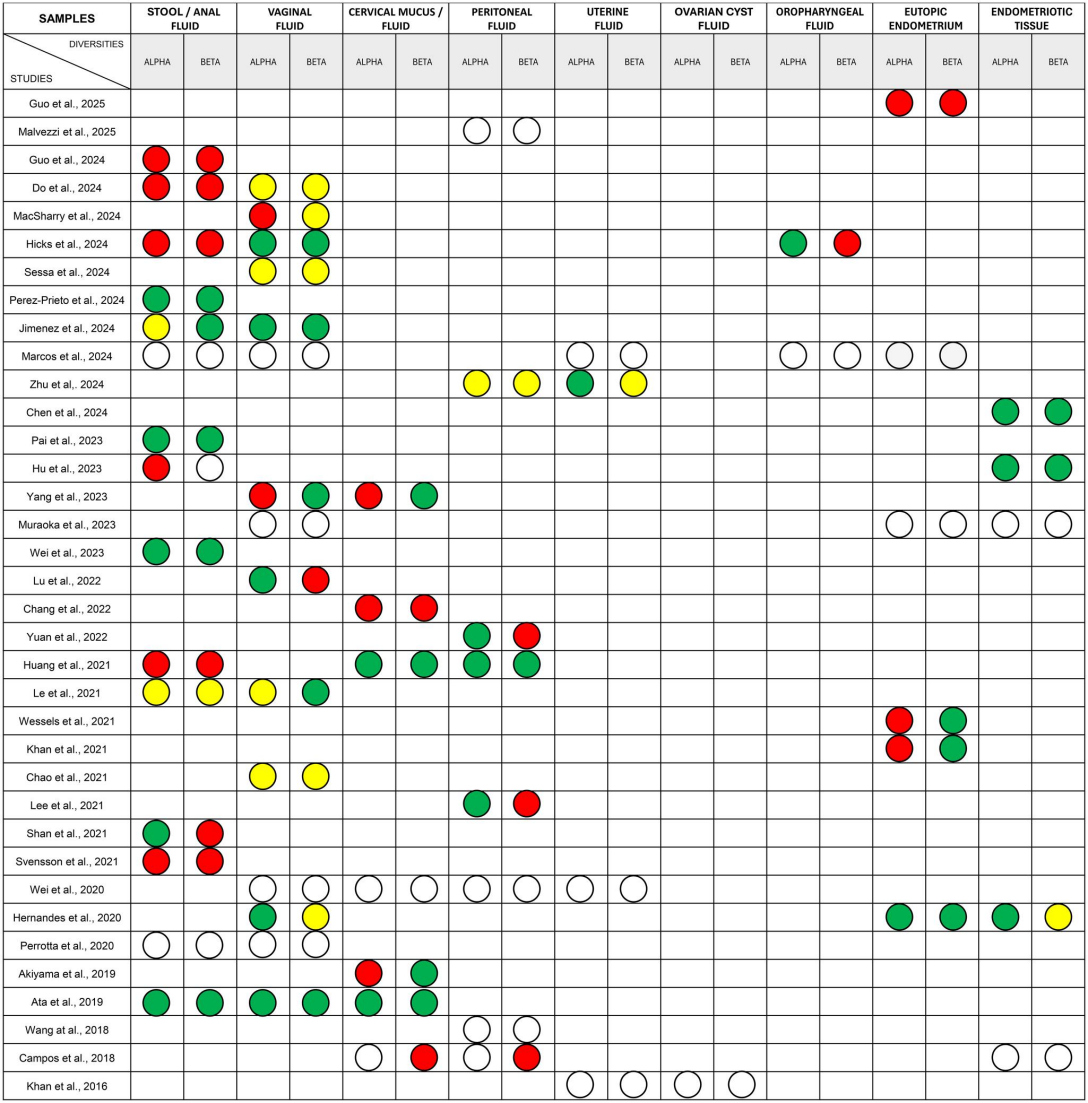
**Alpha diversity (within-sample bacterial diversity) and beta diversity (between-sample bacterial composition differences) comparisons between women with and without endometriosis across studies.** Red, diversity reported as statistically significant; green, diversity reported as not statistically significant; yellow, mixed findings, with some analyses showing statistical significance and others not; white, diversity not reported in the study.

All studies, except one (Perrotta *et al.*, 2020), assessed alpha diversity, within-sample bacterial diversity, and beta diversity, between-sample bacterial composition differences (Fig. 2). Significant differences in alpha diversity were observed in six studies (Huang *et al.*, 2021; Svensson *et al.*, 2021; Hu *et al.*, 2023; Do *et al.* 2024; Hicks *et al.* 2025; Guo *et al.*, 2024), and beta diversity differences were reported in another six (Huang *et al.*, 2021; Shan *et al.*, 2021; Svensson *et al.*, 2021; Do *et al.* 2024; Hicks *et al.* 2025; Guo *et al.*, 2024). However, each of these studies had at least one major methodologic or demographic variable unreported or significantly different between groups, limiting interpretation.

Among the genera reported as significantly differing between women with and without endometriosis, only six genera were identified in more than one study, which were *Eubacterium dolichum* (Huang *et al.*, 2021; Shan *et al.*, 2021), *Haemophilus* sp. (Marcos *et al.*, 2024; Hicks *et al.*, 2025), *Phascolarctobacterium* sp. (Jimenez *et al.*, 2024; Hicks *et al.*, 2025), *Prevotella* sp. (Shan *et al.*, 2021; Hu *et al.*, 2023) with increased abundance in the endometriosis groups while *Fusicatenibacter* sp. (Wei *et al.*, 2023; Guo *et al.*, 2024), and *Lachnospira* sp. (Shan *et al.*, 2021; Svensson *et al.*, 2021; Guo *et al.*, 2024; Hicks *et al.*, 2025) were identified as being more abundant in the control groups.

**Figure 3.**
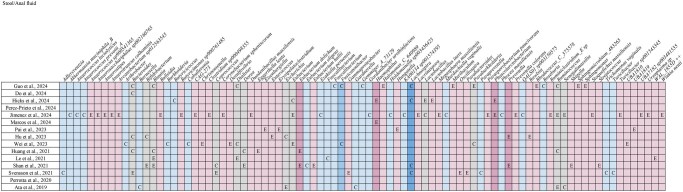
**Bacterial genera identified across stool/anal fluid samples in the included studies.** E, genus’s abundance increased in endometriosis; C, genus’s abundance increased in controls; mid-pink, increased in endometriosis in two studies; light pink, increased in endometriosis in one study; grey, inconsistent findings across studies; light blue, decreased in endometriosis in one study; mid-blue, decreased in endometriosis in two studies; dark blue, decreased in endometriosis in ≥3 studies.

Interestingly, several other genera were reported as differentially abundant between groups but inconsistently since they were found to be increased in the endometriosis group in some studies and in the control group in others, indicating lack of consensus and highlighting variability in study design or populations (*Bacteroides* sp. (Huang *et al.*, 2021; Svensson *et al.*, 2021; Hu *et al.*, 2023; Do *et al.*, 2024; Guo *et al.*, 2024), *Bifidobacterium* sp. (Shan *et al.*, 2021; Hu *et al.*, 2023), *Blautia* sp. (Huang *et al.*, 2021; Le *et al.*, 2021; Shan *et al.*, 2021; Guo *et al.*, 2024), *Coprococccus* sp. (Shan *et al.*, 2021; Svensson *et al.*, 2021), *Dialister* sp. (Le *et al.*, 2021; Wei *et al.*, 2023; Guo *et al.*, 2024), *Dorea* sp. (Huang *et al.*, 2021; Shan *et al.*, 2021), *Escherichia* sp. (Ata *et al.*, 2019; Hu *et al.*, 2023), *Eubacterium* sp. (Wei *et al.*, 2023; Jimenez *et al.*, 2024; Hicks *et al.*, 2025), *Lactobacillus* sp. (Jimenez *et al.*, 2024; Hicks *et al.*, 2025), *Paraburkholderia* sp. (Wei *et al.*, 2023; Guo *et al.*, 2024), *Ruminococcus* sp. (Ata *et al.*, 2019; Huang *et al.*, 2021; Guo *et al.*, 2024; Jimenez *et al.*, 2024), and *Senegalimassilia* sp. (Ata *et al.*, 2019; Jimenez *et al.*, 2024)) (Fig. 3).

At higher taxonomic levels (phylum, class, order, family), 11 studies reported differential abundance (Huang *et al.*, 2021; Le *et al.*, 2021; Shan *et al.*, 2021; Svensson *et al.*, 2021; Hu *et al.*, 2023; Pai *et al.*, 2023; Wei *et al.*, 2023; Guo *et al.*, 2024; Jimenez *et al.*, 2024; Marcos *et al.*, 2024; Hicks *et al.*, 2025), when genus-level resolution was not achieved (Supplementary Table S3).

Overall, no consistent dysbiotic signature was identified across studies. Importantly, only Pérez-Prieto *et al.* (2024) applied Benjamini–Hochberg correction to account for multiple comparisons and reduce the likelihood of type I errors, underscoring a critical gap in statistical rigor in the current literature.

Furthermore, among the studies that reported significant differences at the genus level between women with and without endometriosis, only four (Huang *et al.*, 2021; Shan *et al.*, 2021; Hu *et al.*, 2023; Wei *et al.*, 2023) were considered to be of moderate quality according to the NOS. The remaining studies (Svensson *et al.*, 2021; Guo *et al.*, 2024; Jimenez *et al.*, 2024; Marcos *et al.*, 2024; Hicks *et al.*, 2025) were assessed as low quality, indicating a higher risk of bias (Supplementary Table S4).

### Microbiome analyses in fluids

A total of 25 studies (Khan *et al.*, 2016; Campos *et al.*, 2018; Wang *et al.*, 2018; Akiyama *et al.*, 2019; Ata *et al.*, 2019; Hernandes *et al.*, 2020; Perrotta *et al.*, 2020; Wei *et al.*, 2020; Chao *et al.*, 2021; Huang *et al.*, 2021; Le *et al.*, 2021; Lee *et al.*, 2021; Chang *et al.*, 2022; Lu *et al.*, 2022; Yuan *et al.*, 2022; Muraoka *et al.*, 2023; Yang *et al.*, 2023; Do *et al.*, 2024; Jimenez *et al.*, 2024; MacSharry *et al.*, 2024; Marcos *et al.*, 2024; Sessa *et al.*, 2024; Zhu *et al.*, 2024; Hicks *et al.*, 2025; Malvezzi *et al.*, 2025) investigated the microbiome composition in various biological fluids to explore associations with endometriosis. Fluids analyzed included vaginal, cervical, peritoneal, uterine, ovarian cyst, and oropharyngeal samples (see Tables 3–8 for study-specific details). Unlike fecal samples, biological fluids are complex matrices with inherently lower microbial biomass and, typically, their collection is more complex. Protocols for DNA extraction and microbiome analysis from these fluids are so far, less standardized, contributing to methodological heterogeneity across studies.

**Table 3.** Technical characteristics of microbiome analyses across vaginal fluid samples in the included studies.

Authors (year)	**Study groups** (cases vs controls)	**Number of samples included in the analysis** (cases vs controls)	**Age (years)** (cases vs controls)	**BMI (kg/m^2^)** (cases vs controls)	Sampling technique	**Extraction kit—brand** *(its indication)*	Method	Sequencing platform	Sequencing instrument	Region amplified	Reads length
Do *et al.* (2024)	Women with endometriosis vs women without endometriosis	33 vs 15	31.6 ± 0.8 vs 33.3 ± 1.8 ^§^ (*P*=NS)	28.3 ± 1.3 vs 29.5 ± 1.9 ^§^ (*P*=NS)	‘*[…] vaginal swabs […] samples were collected using aseptic techniques in the operating room on the day of surgery (DOS) and in clinic 1–3 weeks postsurgical intervention (PSI).*’	DNeasy PowerSoil Pro Kit—Qiagen *(For the isolation of microbial genomic DNA from all soil types)*	16S	Illumina	NR	V4	NR
MacSharry *et al.* (2024)	(Women with mild/minimal endometriosis vs women with moderate/severe endometriosis) vs women without endometriosis	(11 vs 10) vs 19	(35 [33–37] vs 33 [30–35]) vs 38 [35–40] ^†^ (*P*=NR)	(23.3 [22.6–24.1] vs 25.6 [22.2–26.8]) vs 23.0 [21.1–28.0] ^†^ (*P*=NR)	‘*High vaginal swabs were collected at laparoscopy.*’	QIAamp UCP Pathogen Mini Kit—Qiagen *(For microbial DNA purification from whole blood, swabs, cultures, and body fluids)*	WGS	Illumina	NovaSeq	Shotgun sequencing	150 bp paired-end
Hicks *et al.* (2025)	Women with endometriosis vs (women with other gynecological diseases vs healthy controls)	21 vs (24 vs 19)	35.9 ± 8.1 vs (35.8 ± 7.3 vs 31.5 ± 3.5) ^§^ (*P*=NS)	NR	‘*[…] vaginal samples were collected using FLOQSwab and eNAT tube collection sets (Copan, Italy)*’	QIAamp DNA Kit—Qiagen *(For isolation of genomic, mitochondrial, bacterial, parasite or viral DNA from tissues, swabs, CSF, blood, body fluids or washed cells from urine)*	16S	Illumina	Miseq	V3–V4	300 bp paired-end
Sessa *et al.* (2024)	Fertile women with endometriosis vs fertile women without endometriosis	24 vs 99	27.4 ± 3.2 vs 25 ± 5.7 ^§^ (*P*=NS)	22.5 ± 3.3 vs 22.7 ± 4.5 ^§^ (*P*=NS)	‘*A vaginal swab for metagenomic analysis was collected*’	DNeasy Blood and Tissue Kit—Qiagen *(For extraction of total DNA from animal blood and tissues and from cells, yeast, bacteria, or viruses)*	16S	Illumina	Miseq	V3–V4	Paired-end
Jimenez *et al.* (2024)	Women with chronic pelvic pain with endometriosis vs women with other gynecological disorders without chronic pelvic pain	35 vs 15	34.7 ± 8.8 vs 40.6 ± 8.2 ^§^ (*P*=NS)	Reported by three ranges of age (*P*=NS)	‘*Enrolled patients underwent sample collection in the operating room after administration of anesthesia but before the pelvic exam, lubricant use, or vaginal prep administration. […], and sterile swabs were used to collect samples from the vagina […] utilizing an established protocol*’	DNeasy PowerSoil Pro Kit—Qiagen *(For the isolation of microbial genomic DNA from all soil types)*	16S	Illumina	MiSeq	V4	NR
Marcos *et al.* (2024)	Infertile women with endometriosis vs women with other infertility-related conditions	8 vs 13	42.7 ± 5.5 vs 39.4 ± 3.7 ^§^ (*P*=NS)	NR	‘*[…] External genitalia* were* cleansed with a saline solution (NaCl 0.9%), and a sterile viscose swab (Deltalab, Barcelona, Spain) was introduced until the posterior fornix was reached and rotated to soak the swab for ∼1 min. After cleaning the external genital area, a speculum was Introduced into the vagina, and a sterile swab was taken from the posterior area rotating 360 degrees for 1 min;*	QIAamp Fast DNA Tissue Kit—Qiagen *(For rapid isolation of genomic DNA from solid tissue samples)*	16S	Ion Torrent	PGM	NR	Single-end
Yang *et al.* (2023)	Women with endometrioma vs healthy controls	19 vs 21	29 [28–37] vs 37 [34–40] ^†^ (*P*=NR)	NR	‘*Vaginal swabs were collected before gynecological examination […] from the posterior vaginal fornix.*’	DNeasy PowerLyzer PowerSoil Kit—Qiagen *(For isolation of DNA from tough soil microbes)*	16S	Illumina	NovaSeq	V4–V5	250 bp
Muraoka *et al.* (2023)	Women with endometriosis vs women without endometriosis	10 vs 10	34.5 [31.0–39.0] vs 34.5 [32.0–37.0] ^†^ (*P*=NR)	NR	‘*The vaginal samples were collected using cotton swab (DNA/RNA Shield Collection Tube With Swab, ZYMO RESEARCH) to absorb vaginal secretions.*’	QIAamp DNA Microbiome Kit—Qiagen *(For isolation of bacterial microbiome DNA from swab and body fluids)*	qRT-PCR	NR	*Primers for F. nucleatum.*		
Lu *et al.* (2022)	Women with endometriosis vs healthy controls	16 vs 18	36.75 ± 7.11 vs 35 ± 6.61 ^§^ (*P*=NS)	20.64 ± 3.04 vs 19.75 ± 1.47 ^§^ (*P*=NS)	NR	TIANamp Bacteria DNA Kit—TianGen *(For genomic DNA extraction from Gram-negative, Gram-positive bacteria, and pathogenic bacteria of food)*	16S	Illumina	HiSeq 2000	V4	NR
Le *et al.* (2021)	Women with endometriosis vs women with other benign gynecological indications	20 vs 9	32.5 ± 1.1 vs 32.6 ± 2.0 ^§^ (*P*=NS)	26.5 ± 1.5 vs 28.1 ± 2.4 ^§^ (*P*=NS)	‘*Sample collection methods were performed by trained medical professionals […] per the Human Microbiome Project protocol.*’	PowerMag Soil DNA Isolation Kit—MoBio *(For isolation of microbial DNA from all types of soil)*	16S	Illumina	Miseq	V4	250 bp paired-end
Chao *et al.* (2021)	Women with CPP with endometriosis vs (women with CPP without endometriosis vs healthy control)	37 vs (25 vs 66)	39.89 ± 6.24 vs *(*37.56 ± 5.480 vs 38.23 ± 7.80) ^§^ (*P*=NR)	NR	‘*The vagina and cervix were fully exposed by a sterile, disposable speculum without lubricant, and samples were obtained from the posterior vaginal fornix.*’	Cetyltrimethylammonium bromide (CTAB) *(Generic method for isolating genomic DNA from different tissues)*	16S	Illumina	HiSeq 2500	V4	NR
Wei *et al.* (2020)	Women with endometriosis vs women without endometriosis with other benign gynecological conditions	36 vs 14	NR	NR	‘*A sterile swab rotated gently for three to five circles to obtain the secretion.*’	QIAamp DNA Kit—Qiagen *(For isolation of genomic, mitochondrial, bacterial, parasite or viral DNA from tissues, swabs, CSF, blood, body fluids or washed cells from urine)*	16S	Ion Torrent	PGM	V4–V5	NR
Hernandes *et al.* (2020)	Women with endometriosis vs women without endometriosis with other benign gynecological conditions	10 vs 11	NR	NR	‘*Vaginal fluid was samples with sterile nylon flock swabs. […] every care was taken to prevent the swab from touching other tissues adjacent to the target site, not having contact with blood, or any other instrument.*’	QIAamp DNA Blood Kit—Qiagen *(For purification of genomic, mitochondrial or viral DNA from blood and other body fluids)*	16S	Illumina	MiSeq	V3–V4	300 nt single-end
Perrotta *et al.* (2020)	Women with endometriosis vs women without endometriosis	35 vs 24	34.9 ± 6.8 vs 35.25 ± 6.9 ^§^ (*P*=NS)	24.8 ± 4.5 vs 24.3 ± 2.7 ^§^ (*P*=NS)	‘*Samples were obtained using Sterile Catch-All sample collection swabs (Fisher Scientific, Pittsburgh, PA, USA). Vaginal samples were collected in the mid-vagina at the site of the vaginal introitus*’	PowerMag Soil DNA Isolation Kit—MoBio *(For isolation of microbial DNA from all types of soil)*	16S	Illumina	Miseq	V4	2× 250 bp paired-end
Ata *et al.* (2019)	Women with endometriosis vs healthy controls	14 vs 14	28.5 [26–31.3] vs 27.5 [25.8–30] ^†^ (*P*=NS)	23 (21–24.3) vs 21 (20.1–24.2) ^†^ (*P*=NS)	‘*Vaginal swabs were collected following the insertion of a sterile vaginal speculum.*’	QuickGene DNA Extraction Tissue Kit S—Biotec *(For isolation of genomic DNA)*	16S	Illumina	Miseq	V3–V4	300 bp paired-end

Data reported as reported by the original papers, unless otherwise stated.

bp = base pairs; CPP = chronic pelvic pain syndrome; NR = not reported; NR = not reported; NS = non-significant; nt = nucleotides;.

LVFX, levofloxacin; qRT-PCR, quantitative real time-PCR; WGS, whole-genome sequencing.

§ Data are expressed as mean±SD.

† Data are expressed as median [25th–75th percentile].

**Table 4.** Technical characteristics of microbiome analyses across cervical fluid samples in the included studies.

Authors (year)	**Study groups** (cases vs controls)	**Number of samples included in the analysis** (cases vs controls)	**Age (years)** (cases vs controls)	**BMI (kg/m^2^)** (cases vs controls)	Sampling technique	**Extraction kit—brand** *(its indication)*	Method	Sequencing platform	Sequencing instrument	Region amplified	Reads length
Yang *et al.* (2023)	Women with endometrioma vs healthy controls	19 vs 21	29 [28–37] vs 37 [34–40] ^†^ (*P*=NR)	NR	‘*Cervical swabs were collected before gynecological examination […] from the cervical canal.*’	DNeasy PowerLyzer PowerSoil Kit—Qiagen *(For isolation of DNA from tough soil microbes)*	16S	Illumina	NovaSeq	V4–V5	250 bp
Chang *et al.* (2022)	Women with endometriosis vs healthy controls	23 vs 10	35 [30–39] ^†^ vs NR	NR	*Swabs; details NR.*	TC Genomic DNA Isolation Kit—Fair Biotech *(For genomic DNA isolation from tissue samples)*	16S	Illumina	NR	V3, V4, V5, and V9	Paired-end
Huang *et al.* (2021)	Women with endometriosis vs women without endometriosis	21 vs 20	38.3 ± 7.88 vs 34.0 ± 10.8 ^§^ (*P*=NS)	21.5 ± 2.79 vs 24.3 ± 8.16 ^§^ (*P*=NS)	‘*Cervical mucus was drawn from the cervical canal with sterile swabs in the process of a gynecological examination (without any prior disturbance; carefully avoid contamination from vaginal).*’	Quick-RNA Fecal/Soil Microbe Microprep Kit—Zymo Research *(For extract of RNA from various soil, fecal, and water samples)*	16S	Ion Torrent	S5	V4	Paired-end
Wei *et al.* (2020)	Women with endometriosis vs women without endometriosis with other benign gynecological conditions	36 vs 14	NR	NR	‘*A sterile swab rotated gently for three to five circles to obtain the secretion.*’	QIAamp DNA Kit—Qiagen *(For isolation of genomic, mitochondrial, bacterial, parasite or viral DNA from tissues, swabs, CSF, blood, body fluids or washed cells from urine)*	16S	Ion Torrent	PGM	V4–V5	NR
Akiyama *et al.* (2019)	Women with endometriosis vs women without endometriosis	30 vs 39	33.9 ± 5.7 vs 32.5 ± 6.0 ^§^ (*P*=NS)	21.3 ± 3.2 vs 20.5 ± 2.8 ^§^ (*P*=NS)	‘*Cervical mucus was aspirated from the cervical canal using a sterile 1‐ml syringe after prudently wiping with a sterile swab*’	NucleoSpin Microbial DNA Mini kit—Macherey‐Nagel *(For Isolation of total DNA from Gram-positive and -negative bacteria, yeast, and fungi)*	16S	Ion Torrent	PGM	V5–V6	250 bp
Ata *et al.* (2019)	Women with endometriosis vs healthy controls	14 vs 14	28.5 [26–31.3] vs 27.5 [25.8–30] ^†^ (*P*=NS)	23 (21–24.3) vs 21 (20.1–24.2) ^†^ (*P*=NS)	‘*Endocervical swabs were collected following the insertion of a sterile vaginal speculum. […] The swabs used to collect endocervical samples did not touch the vaginal walls during sample collection.*’	QuickGene DNA Extraction Tissue Kit S—Biotec *(For isolation of genomic DNA)*	16S	Illumina	MiSeq	V3–V4	300 bp paired-end
Campos *et al.* (2018)	Women with endometriosis vs women without endometriosis	73 vs 31	36 [15–49] vs 39 [26–51] ^†^ (*P*=NS)	Reported by three ranges of age (*P*=NS)	‘*Endocervical swabs were collected before laparoscopy.*’	PureLink Genomic DNA Mini Kit—Invitrogen *(For genomic DNA purification from blood, tissues, cells, bacteria, swabs, and blood spots)*	qRT-PCR	PCR Array kit (Qiagen-SABioscience)	‘*Nucleic acid extracts were tested against M. hominis, M. genitalium, Ureaplasma urealyticum, and U. parvum.*’		

Data reported as reported by the original papers, unless otherwise stated.

Bp, base pairs; NR, not reported; NS, non-significant; qRT-PCR, quantitative real time-PCR.

§ Data are expressed as mean±SD.

† Data are expressed as median [25th–75th percentile].

**Table 5.** Technical characteristics of microbiome analyses across peritoneal fluid samples in the included studies.

Authors (year)	**Study groups** (cases vs controls)	**Number of samples included in the analysis** (cases vs controls)	**Age (years)** (cases vs controls)	**BMI (kg/m^2^)** (cases vs controls)	Sampling technique	**Extraction kit—brand** *(its indication)*	Method	Sequencing platform	Sequencing instrument	Region amplified	Reads length
Malvezzi *et al.* (2025)	Women with endometriosis vs women without endometriosis	27 vs 23	34[31–41] vs 42[34–46] ^†^ (*P* = 0.029)	23[21–28] vs 26[23–29] ^†^ (*P*=NS)	‘*Peritoneal fluid samples were collected upon visual inspection of the cul de sac at the outset of surgery to prevent blood contamination.*’	QIAamp DNA Kit—Qiagen *(For isolation of genomic, mitochondrial, bacterial, parasite or viral DNA from tissues, swabs, CSF, blood, body fluids or washed cells from urine)*	16S	Illumina	MiSeq	V3–V4	300bp, single end
Zhu *et al.* (2024)	(Infertile women with endometriosis stage I/II vs stage III/IV) vs women with tubal obstruction-related infertility	(8 vs 18) vs 31	(28.8 ± 4.4 vs 31.1 ± 5.6) vs 31.0 ± 5.3 ^§^ (*P*=NS)	(20.88 ± 2.05 vs 20.60 ± 2.83) vs 23.14 ± 2.98 ^§^ (*P* = 0.007)	‘*A syringe was used to connect the aspirator, and ∼5–10 ml of PF were aspirated from the Douglas pouch.*’	MagPure Soil DNA Kit—Magen *(For isolation of high-quality genomic DNA from various soil, stool, and other environmental samples)*	16S	Illumina	NovaSeq 6000	V3–V4	250 bp paired-end
Yuan *et al.* (2022)	Women with endometriosis vs women without endometriosis	36 vs 25	35.28 ± 7.24 vs 33.32 ± 8.04 ^§^ (*P*=NS)	20.9 ± 2.11 vs 21.4 ± 2.03 ^§^ (*P*=NS)	‘*10 ml PF samples from each participant were obtained from the pouch of Douglas immediately after the insertion under aseptic operating room conditions to minimize contamination from other tissues or objects.*’	Chloroform/Isoamyl Alcohol *(Generic method for purifying DNA from cells and soft tissues)*	16S	Illumina	HiSeq 2500	V4	250 bp paired-end
Huang *et al.* (2021)	Women with endometriosis vs women without endometriosis	21 vs 20	38.3 ± 7.88 vs 34.0 ± 10.8 ^§^ (*P*=NS)	21.5 ± 2.79 vs 24.3 ± 8.16 ^§^ (*P*=NS)	‘*Peritoneal fluid was obtained from participants undergoing a diagnostic laparoscopy.*’	Quick-RNA Fecal/Soil Microbe Microprep Kit—Zymo Research *(For extract of RNA from various soil, fecal, and water samples)*	16S	Ion Torrent	S5	V4	NR
Lee *et al.* (2021)	Women with endometriosis vs women without endometriosis	45 vs 45	36.2 ± 1.3 vs 39.4 ± 1.1 ^§^ (*P*=NS)	36.2 ± 1.3 vs 39.4 ± 1.1 ^§^ (*P*=NS)	‘*PF was obtained from the posterior cul-de-sac or utero-vesical pouch through the laparoscopic cannula during laparoscopic surgery.*’	PowerMag Soil DNA Isolation Kit—MoBio *(For isolation of microbial DNA from all types of soil)*	16S	Illumina	MiSeq	V3–V4	NR
Wei *et al.* (2020)	Women with endometriosis vs women without endometriosis with other benign gynecological conditions	36 vs 14	NR	NR	‘*During the surgery, about 10 ml peritoneal fuid from Douglas pouch were extracted […], placed in 15 ml centrifuge tubes. Samples were immediatly pre-frozen in dry-ice*’	QIAamp DNA Kit—Qiagen *(For isolation of genomic, mitochondrial, bacterial, parasite or viral DNA from tissues, swabs, CSF, blood, body fluids or washed cells from urine)*	16S	Ion Torrent	PGM	V4–V5	NR
Wang *et al.* (2018)	Infertile women with endometriosis vs infertile women without endometriosis	55 vs 30	37.2 ± 8.2 vs 37.7 ± 7.4 ^§^ (*P*=NS)	22.5 ± 2.3 vs 22.9 ± 2.1 ^§^ (*P*=NS)	*Under the direct vision of laparoscope, the trocar was inserted to quickly extract 10 ml peritoneal fluid from the vesicouterine pouch and rectovaginal pouch;*	MagicPure Soil and Stool Genomic DNA Kit–TransGen Biotech *(For DNA purification from various types of soil and stool samples)*	16S	Ion Torrent	PGM	V5–V4	NR
Campos *et al.* (2018)	Women with endometriosis vs women without endometriosis	54 vs 24	NR	NR	‘*During laparoscopy, following auxiliary puncture, the peritoneal fluid deposited in the anterior and posterior Douglas pouches was collected*’	PureLink Genomic DNA Mini Kit—Invitrogen *(For genomic DNA purification from blood, tissues, cells, bacteria, swabs, and blood spots)*	qRT-PCR	PCR Array kit (Qiagen-SABioscience)	‘*Nucleic acid extracts were tested against M. hominis, M. genitalium, Ureaplasma urealyticum, and U. parvum.*’		

Data reported as reported by the original papers, unless otherwise stated.

Bp, base pairs; NR, not reported; NS, non-significant; qRT-PCR, quantitative real time-PCR.

§ Data are expressed as mean±SD.

† Data are expressed as median [25th–75th percentile].

**Table 6.** Technical characteristics of microbiome analyses across uterine fluid samples in the included studies.

Authors (year)	**Study groups** (cases vs controls)	**Number of samples included in the analysis** (cases vs controls)	**Age (years)** (cases vs controls)	**BMI (kg/m^2^)** (cases vs controls)	Sampling technique	**Extraction kit—brand** *(its indication)*	Method	Sequencing platform	Sequencing instrument	Region amplified	Reads length
Marcos *et al.* (2024)	Infertile women with endometriosis vs women with other infertility-related conditions	8 vs 13	42.7 ± 5.5 vs 39.4 ± 3.7 ^§^ (*P*=NS)	NR	‘*[…] the endometrial fluid sample was obtained after the vaginal fluid sample and before the endometrial biopsy. A flexible catheter was inserted 5–8 cm through the cervix into the ultrasound-guided uterine cavity, and ∼80 μl of endometrial fluid was gradually removed with a syringe. Once the sample was collected, suction was stopped, and the catheter was removed.*’	QIAamp Fast DNA Tissue Kit—Qiagen *(For rapid isolation of genomic DNA from solid tissue samples)*	16S	Ion Torrent	PGM	NR	Single-end
Zhu *et al.* (2024)	(Infertile women with endometriosis stage I/II vs stage III/IV) vs women with tubal obstruction-related infertility	(8 vs 18) vs 31	(28.8 ± 4.4 vs 31.1 ± 5.6) vs 31.0 ± 5.3 ^§^ (*P*=NS)	(20.88 ± 2.05 vs 20.60 ± 2.83) vs 23.14 ± 2.98 ^§^ (*P* = 0.007)	‘*[…]hysteroscope outfitted with a sterile saline infusion system was utilized. Sterile saline was carefully infused into the uterine cavity, and after allowing the solution to interact with the endometrium for a period of 1 min, it was then evacuated through the hysteroscope’s outflow channel.*’	MagPure Soil DNA Kit—Magen *(For isolation of high-quality genomic DNA from various soil, stool, and other environmental samples)*	16S	Illumina	NovaSeq 6000	V3–V4	250 bp, paired-end
Wei *et al.* (2020)	Women with endometriosis vs women without endometriosis with other benign gynecological conditions	36 vs 14	NR	NR	‘*Endometrial fluid samples were obtained by injecting 2 ml of sterile saline to uterine cavity using a sampler (Huales Medical Machinery, Ningbo), then absorbing the uterine lavage fuid after 1 min and placing in the 5 ml tubes.*’	QIAamp DNA Kit—Qiagen *(For isolation of genomic, mitochondrial, bacterial, parasite or viral DNA from tissues, swabs, CSF, blood, body fluids or washed cells from urine)*	16S	Ion Torrent	PGM	V4–V5	NR
Khan *et al.* (2016)	(Women with endometriosis using GnRHa vs not using GnRHa) vs (women without endometriosis using GnRH analogue vs not using GnRH analogue)	(16 vs 16) vs (16 vs 16)	(37.5 ± 5.6 vs 35.7 ± 8.3) vs (42.1 ± 8.6 vs 33.6 ± 8.9; *P* < 0.01) ^§^ (*P*=NR)	NR	‘*The seed swab was inserited under visual control into the uterine….*’	UltraClean^®^ Soil DNA Isolation Kit—MoBio *(For isolate cellular, PCR quality DNA from soil)*	16S	Illumina	MiSeq	NR	NR

Data reported as reported by the original papers, unless otherwise stated.

Bp, base pairs; NR, not reported; NS, non-significant.

§ Data are expressed as mean±SD.

**Table 7.** Technical characteristics of microbiome analyses across ovarian cyst fluid samples in the included studies.

Authors (year)	**Study groups** (cases vs controls)	**Number of samples included in the analysis** (cases vs controls)	**Age (years)** (cases vs controls)	**BMI (kg/m^2^)** (cases vs controls)	Sampling technique	**Extraction kit—brand** *(its indication)*	Method	Sequencing platform	Sequencing instrument	Region amplified	Reads length
Khan *et al.* (2016)	Women with endometrioma not using GnRH analogue vs women with serous/mucinous cyst adenoma not using GnRH analogue	8 vs 8	NR	NR	‘*Cystic fluid was collected during laparoscopy.*’	UltraClean^®^ Soil DNA Isolation Kit—MoBio *(For isolate cellular, PCR quality DNA from soil)*	16S	Illumina	Miseq	NR	NR

Data reported as reported by the original papers, unless otherwise stated.

NR, not reported.

**Table 8.** Technical characteristics of microbiome analyses across oropharyngeal fluid samples in the included studies.

Authors (year)	**Study groups** (cases vs controls)	**Number of samples included in the analysis** (cases vs controls)	**Age (years)** (cases vs controls)	**BMI (kg/m^2^)** (cases vs controls)	Sampling technique	**Extraction kit—brand** *(its indication)*	Method	Sequencing platform	Sequencing instrument	Region amplified	Reads length
Hicks *et al.* (2025)	Women with endometriosis vs (women with other gynecological diseases vs healthy controls)	21 vs (24 vs 19)	35.9 ± 8.1 vs (35.8 ± 7.3 vs 31.5 ± 3.5) ^§^ (*P*=NS)	NR	‘*Oral […] samples were collected using FLOQSwab and eNAT tube collection sets (Copan, Italy)*’	QIAamp DNA Kit—Qiagen *(For isolation of genomic, mitochondrial, bacterial, parasite or viral DNA from tissues, swabs, CSF, blood, body fluids or washed cells from urine)*	16S	Illumina	Miseq	V3–V4	300 bp paired-end
Marcos *et al.* (2024)	Infertile women with endometriosis vs women with other infertility-related conditions	8 vs 13	42.7 ± 5.5 vs 39.4 ± 3.7 ^§^ (*P*=NS)	NR	‘*[…] with a sterile swab rotating for 60 s around the* (oral) *mucosa.*’	QIAamp Fast DNA Tissue Kit—Qiagen *(For rapid isolation of genomic DNA from solid tissue samples)*	16S	Ion Torrent	PGM	NR	Single-end

Data reported as reported by the original papers, unless otherwise stated.

Bp, base pairs; NR, not reported; NS, non-significant.

§ Data are expressed as mean±SD.

Consequently, it is not surprising that DNA extraction methods varied substantially. The majority of studies (Khan *et al.*, 2016; Wang *et al.*, 2018; Hernandes *et al.*, 2020; Perrotta *et al.*, 2020; Chao *et al.*, 2021; Huang *et al.*, 2021; Lee *et al.*, 2021; Chang *et al.*, 2022; Lu *et al.*, 2022; Yuan *et al.*, 2022; Yang *et al.*, 2023; Do *et al.*, 2024; Jimenez *et al.*, 2024; Marcos *et al.*, 2024; Sessa *et al.*, 2024; Zhu *et al.*, 2024) employed DNA extraction kits not specifically designed for fluid samples, using kits optimized for soil, feces, or tissues. For instance, one study (Chao *et al.*, 2021) used cetyltrimethylammonium bromide (CTAB), a general-purpose method traditionally used for plants and tissues, to isolate DNA from vaginal fluid. Similarly, another study (Yuan *et al.*, 2022) extracted DNA from peritoneal fluid using chloroform/isoamyl alcohol, a standard method for DNA purification from soft tissues and cells.

Despite the use of extraction methods not tailored for low-biomass fluid samples or microbiome-specific applications, most studies reported adequate DNA recovery, enabling successful sequencing and subsequent microbiome analysis.

#### Vaginal fluid

Technical characteristics of the 15 studies (Ata *et al.*, 2019; Hernandes *et al.*, 2020; Perrotta *et al.*, 2020; Wei *et al.*, 2020; Chao *et al.*, 2021; Le *et al.*, 2021; Lu *et al.*, 2022; Muraoka *et al.*, 2023; Yang *et al.*, 2023; Jimenez *et al.*, 2024; MacSharry *et al.*, 2024; Marcos *et al.*, 2024; Sessa *et al.*, 2024; Hicks *et al.*, 2025) analyzing the vaginal fluid microbiome in women with endometriosis are detailed in Table 3.

Sample sizes were generally small, ranging from n = 8 to n = 37 for endometriosis cases and n = 9 to n = 99 for controls. Only five studies recruited healthy women as controls (Ata *et al.*, 2019; Lu *et al.*, 2022; Yang *et al.*, 2023) or as a subgroup within the control population (Chao *et al.*, 2021; Hicks *et al.*, 2025). Some studies further limited their scope to specific subtypes of endometriosis. For instance, while Muraoka *et al.* (2023) enrolled 144 participants, only n = 10 cases of ovarian endometriosis were included for vaginal fluid analysis. Similarly, Yang *et al.* (2023) and Hernandes *et al.* (2020) focused solely on deep endometriosis, analyzing n = 19 and n = 10 vaginal fluid samples, respectively.

Age was reported and found to be comparable between groups in most studies, though four (Chao *et al.*, 2021; Muraoka *et al.*, 2023; Yang *et al.*, 2023; MacSharry *et al.*, 2024) did not report whether significant differences existed, and two (Hernandes *et al.*, 2020; Wei *et al.*, 2020) did not report age data at all. BMI was not reported in seven studies (Hernandes *et al.*, 2020; Wei *et al.*, 2020; Chao *et al.*, 2021; Muraoka *et al.*, 2023; Yang *et al.*, 2023; Marcos *et al.*, 2024; Hicks *et al.*, 2025).

Diagnosis of endometriosis was primarily surgical, although three studies (Perrotta *et al.*, 2020; Marcos *et al.*, 2024; Sessa *et al.*, 2024) also accepted imaging-based diagnoses, suggesting a focus on moderate to severe cases. However, eight studies (Wei *et al.*, 2020; Chao *et al.*, 2021; Le *et al.*, 2021; Lu *et al.*, 2022; Do *et al.*, 2024; Jimenez *et al.*, 2024; Marcos *et al.*, 2024; Hicks *et al.*, 2025) did not specify the phenotype of endometriosis and two of them (Chao *et al.*, 2021; Marcos *et al.*, 2024) also omitted information on disease stage.

Hormonal treatment was an exclusion criterion in seven studies (Ata *et al.*, 2019; Perrotta *et al.*, 2020; Wei *et al.*, 2020; Lu *et al.*, 2022; Muraoka *et al.*, 2023; Yang *et al.*, 2023; MacSharry *et al.*, 2024) while two studies did not report hormonal treatment status at all (Hernandes *et al.*, 2020; Hicks *et al.*, 2025). Among the remaining studies, several reported hormone usage, but only four (Chao *et al.*, 2021; Le *et al.*, 2021; Do *et al.*, 2024; Jimenez *et al.*, 2024) confirmed balanced distribution across groups. The other two (Marcos *et al.*, 2024; Sessa *et al.*, 2024) did not clarify this distribution.

Regarding antibiotic use, only three studies (Le *et al.*, 2021; MacSharry *et al.*, 2024; Hicks *et al.*, 2025) did not consider antibiotic treatments as an exclusion criterion; furthermore, none of these clarify whether its use was similar between groups.

There was marked inconsistency in accounting for the menstrual cycle phase at the time of vaginal fluid collection. Six studies (Hernandes *et al.*, 2020; Le *et al.*, 2021; Lu *et al.*, 2022; Do *et al.*, 2024; Jimenez *et al.*, 2024; Hicks *et al.*, 2025) did not report this information. One study (Perrotta *et al.*, 2020) collected samples during both menstrual and follicular phases. Among the others, either the distribution of phases was similar between groups (Ata *et al.*, 2019; Chao *et al.*, 2021; Muraoka *et al.*, 2023; MacSharry *et al.*, 2024), or samples were collected during a uniform phase across participants, through the chosen phase differed across studies: ovulatory phase (Marcos *et al.*, 2024; Sessa *et al.*, 2024); early follicular phase (Wei *et al.*, 2020); or follicular phase (Yang *et al.*, 2023).

As with stool and anal fluid studies, dietary factors were not thoroughly considered. No study excluded participants based on special diets (such as vegetarianism), which could influence microbiome composition (Table 1).

Microbiome analysis details are summarized in Table 3. The majority of studies utilized 16S rRNA gene sequencing on Illumina or Ion Torrent platforms (Ata *et al.*, 2019; Hernandes *et al.*, 2020; Perrotta *et al.*, 2020; Wei *et al.*, 2020; Chao *et al.*, 2021; Le *et al.*, 2021; Lu *et al.*, 2022; Yang *et al.*, 2023; Do *et al.*, 2024; Jimenez *et al.*, 2024; Marcos *et al.*, 2024; Sessa *et al.*, 2024; Hicks *et al.*, 2025). Only one study (MacSharry *et al.*, 2024) conducted shotgun metagenomic paired-end sequencing, a technique based on whole-genome sequencing.

In one case (Muraoka *et al.*, 2023), the analysis involved bioinformatic reanalysis of previously deposited datasets (The European Nucleotide Archive: PRJEB16013 and PRJEB21098), followed by quantitative real-time polymerase chain reaction (qRT-PCR) to detect a specific bacterial species (Supplementary Table S2). Bioinformatics pipelines, including sequence filtering, chimera removal, OTU clustering, and taxonomic assignment, varied widely across studies. A detailed overview is provided in Supplementary Table S2.

Alpha and beta diversity metrics were reported in nearly all studies (Fig. 2), with the exception of four (Perrotta *et al.*, 2020; Wei *et al.*, 2020; Muraoka *et al.*, 2023; Marcos *et al.*, 2024). Significant differences in alpha diversity between endometriosis cases and controls were consistently reported in only two studies (Yang *et al.*, 2023; MacSharry *et al.*, 2024). Four additional studies observed differences only in specific sub-analyses (Chao *et al.*, 2021; Le *et al.*, 2021; Do *et al.*, 2024; Sessa *et al.*, 2024).

Beta diversity findings were similarly variable. Five studies (Ata *et al.*, 2019; Le *et al.*, 2021; Yang *et al.*, 2023; Jimenez *et al.*, 2024; Hicks *et al.*, 2025) reported no significant difference in community composition between groups, while one study (Lu *et al.*, 2022) found a significant overall difference. Five other studies (Hernandes *et al.*, 2020; Chao *et al.*, 2021; Do *et al.*, 2024; MacSharry *et al.*, 2024; Sessa *et al.*, 2024) reported differences only within subgroup analyses.

**Figure 4.**
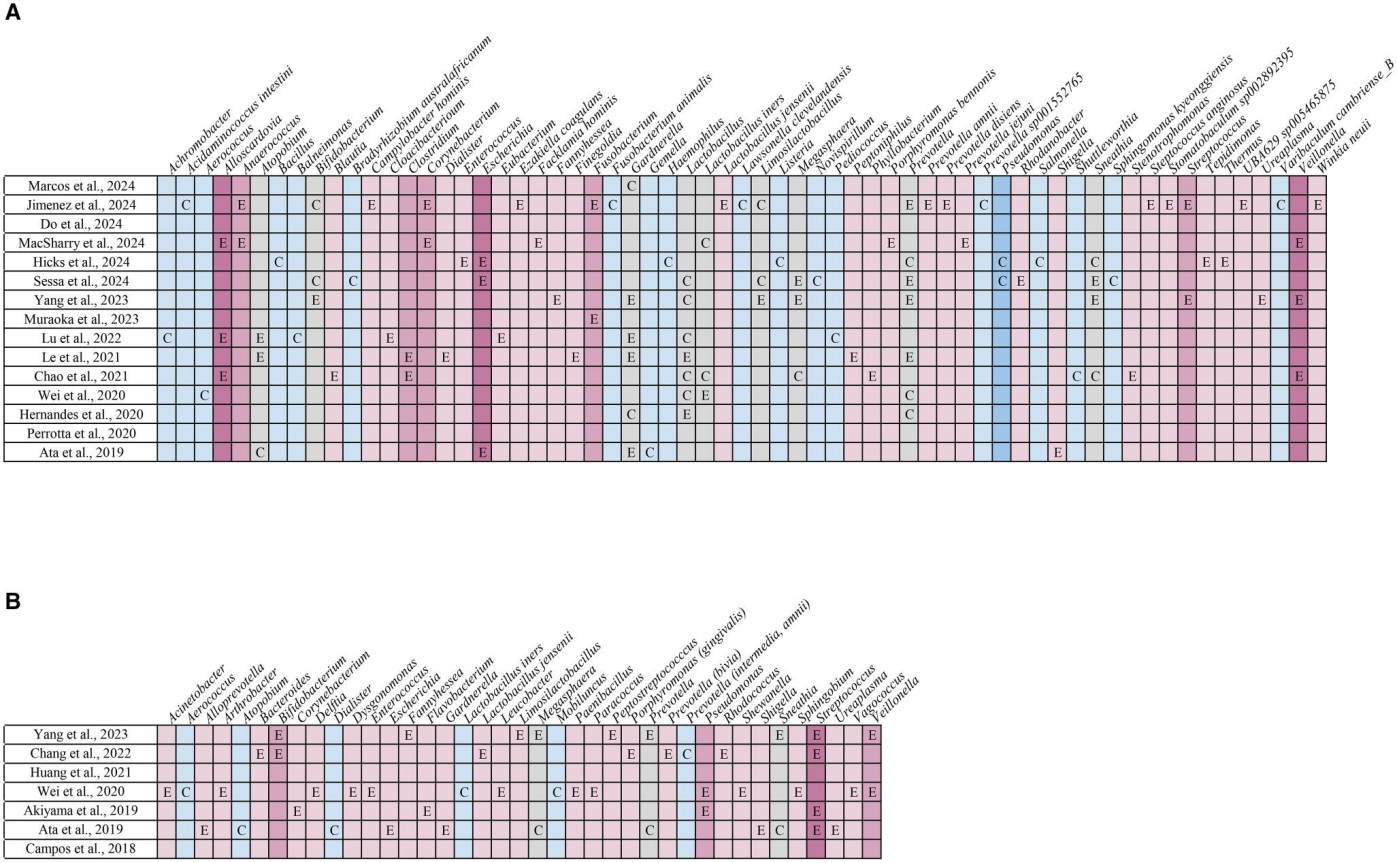
**Bacterial genera identified across vaginal and cervical samples in the included studies.** E, genus’s abundance increased in endometriosis; C, genus’s abundance increased in controls; dark pink, increased in endometriosis in ≥3 studies; mid-pink, increased in endometriosis in two studies; light pink, increased in endometriosis in one study; grey, inconsistent findings across studies; light blue, decreased in endometriosis in one study; mid-blue, decreased in endometriosis in two studies. (**A**) Vaginal fluid. (**B**) Cervical fluid.

Among the genera found to differ significantly between endometriosis and control groups, only nine taxa were reported in two or more studies with consistent findings (Fig. 4A). *Alloscardovia* sp. (Chao *et al.*, 2021; Lu *et al.*, 2022; MacSharry *et al.*, 2024), *Anaerococcus* sp. (Jimenez *et al.*, 2024; MacSharry *et al.*, 2024), *Clostridium* sp. (Chao *et al.*, 2021; Le *et al.*, 2021), *Corynebacterium* sp. (Jimenez *et al.*, 2024; MacSharry *et al.*, 2024), *Escherichia* sp. (Ata *et al.*, 2019; Sessa *et al.*, 2024; Hicks *et al.*, 2025), *Fusobacterium* sp. (Muraoka *et al.*, 2023; Jimenez *et al.*, 2024), *Streptococcus* sp. (Yang *et al.*, 2023; Jimenez *et al.*, 2024), and *Veillonella* sp. (Chao *et al.*, 2021; Yang *et al.*, 2023; MacSharry *et al.*, 2024) had increased abundance in the endometriosis groups while *Pseudomonas* sp. (Sessa *et al.*, 2024; Hicks *et al.*, 2025) was identified as being more abundant in the control groups. Other genera exhibited inconsistent trends, showing increased abundance in endometriosis in some studies and in controls in others, such as *Atopobium* sp. (Ata *et al.*, 2019; Le *et al.*, 2021; Lu *et al.*, 2022), *Bifidobacterium* (Yang *et al.*, 2023; Jimenez *et al.*, 2024; Sessa *et al.*, 2024), *Gardnerella* sp. (Ata *et al.*, 2019; Hernandes *et al.*, 2020; Le *et al.*, 2021; Lu *et al.*, 2022; Yang *et al.*, 2023; Marcos *et al.*, 2024), *Lactobacillus* sp. (Hernandes *et al.*, 2020; Wei *et al.*, 2020; Chao *et al.*, 2021; Le *et al.*, 2021; Lu *et al.*, 2022; Yang *et al.*, 2023; MacSharry *et al.*, 2024; Sessa *et al.*, 2024), *Limosilactobacillus* sp. (Yang *et al.*, 2023; Jimenez *et al.*, 2024; Sessa *et al.*, 2024), *Megasphaera* sp. (Yang *et al.*, 2023; Sessa *et al.*, 2024), *Prevotella* (Hernandes *et al.*, 2020; Wei *et al.*, 2020; Le *et al.*, 2021; Yang *et al.*, 2023; Jimenez *et al.*, 2024; Sessa *et al.*, 2024; Hicks *et al.*, 2025), *Sneathia* sp. (Chao *et al.*, 2021; Yang *et al.*, 2023; Sessa *et al.*, 2024; Hicks *et al.*, 2025). One study (Perrotta *et al.*, 2020) conducted a subgroup analysis by endometriosis stage and found that *Anaerococcus* sp. was significantly increased in stage III-IV disease compared to stage I-II. Furthermore, among studies that reported significant differences at the genus level between women with and without endometriosis, half of them (Le *et al.*, 2021; Lu *et al.*, 2022; Muraoka *et al.*, 2023; Jimenez *et al.*, 2024; Hicks *et al.*, 2025) were considered to be of low or moderate quality according to the NOS. In contrast, the remaining studies (Chao *et al.*, 2021; Yang *et al.*, 2023; MacSharry *et al.*, 2024; Sessa *et al.*, 2024) were assessed as high quality, indicating a low risk of bias (Supplementary Table S4).

Seven studies also reported microbial differences at higher taxonomic ranks (phylum, class, order, or family) (Wei *et al.*, 2020; Chao *et al.*, 2021; Le *et al.*, 2021; Lu *et al.*, 2022; Marcos *et al.*, 2024; Sessa *et al.*, 2024; Hicks *et al.*, 2025) (Supplementary Table S3).

#### Cervical fluid or mucus

Technical characteristics of the five studies investigating the microbiome of cervical fluid (Campos *et al.*, 2018; Ata *et al.*, 2019; Huang *et al.*, 2021; Chang *et al.*, 2022; Yang *et al.*, 2023) and the two in the cervical mucus (Akiyama *et al.*, 2019; Wei *et al.*, 2020) are detailed in Table 4.

The sample sizes varied considerably: the number of endometriosis cases ranged from n = 14 to n = 73, and controls from n = 10 to n = 39. Only three studies (Ata *et al.*, 2019; Chang *et al.*, 2022; Yang *et al.*, 2023) included healthy women as controls, whereas the others used symptomatic women with other gynecological conditions.

Demographic data such as age and BMI were inconsistently reported. Age was found to be comparable between groups in most studies, except for one (Yang *et al.*, 2023) that did not report whether differences were significant, and two studies (Wei *et al.*, 2020; Chang *et al.*, 2022), which did not report age data for at least one group. BMI was not reported in three studies (Wei *et al.*, 2020; Chang *et al.*, 2022; Yang *et al.*, 2023), while others confirmed no significant difference between groups.

All studies confirmed endometriosis diagnosis via surgery and histological analysis, and most included women with moderate to severe disease. However, four studies (Akiyama *et al.*, 2019; Wei *et al.*, 2020; Huang *et al.*, 2021; Chang *et al.*, 2022) did not specify the phenotype of endometriosis. Only Yang *et al.* (2023) limited their analysis to endometrioma cases (Table 1).

Regarding hormonal treatments, five studies (Akiyama *et al.*, 2019; Ata *et al.*, 2019; Wei *et al.*, 2020; Huang *et al.*, 2021; Yang *et al.*, 2023) excluded participants receiving hormonal therapy. One study (Chang *et al.*, 2022) did not report on hormonal treatment, while another (Campos *et al.*, 2018) allowed inclusion of participants undergoing treatment, with reported use in 30.1% of cases and 16.1% of controls, though statistical significance of this difference was not provided.

Only one study (Chang *et al.*, 2022) did not consider antibiotic use as an exclusion criterion and did not report whether usage differed between groups.

Menstrual cycle phase at sample collection was not standardized: two studies (Chang *et al.*, 2022; Yang *et al.*, 2023) did not report the cycle phase; one (Wei *et al.*, 2020) collected samples in the early follicular phase; another (Yang *et al.*, 2023) consistently used the follicular phase.The other studies collected samples during either the follicular or luteal phase. Some reported no significant differences between phases within groups (Campos *et al.*, 2018; Akiyama *et al.*, 2019; Ata *et al.*, 2019) while one study did not specify whether a difference existed (Huang *et al.*, 2021).

No study in this group considered dietary habits as an exclusion criterion.

Microbiome analysis details are summarized in Table 4. All studies used 16S rRNA sequencing on Illumina or Ion Torrent platforms, except for one (Campos *et al.*, 2018), who applied qRT-PCR to detect specific bacterial species (Supplementary Table S2). However, the 16S regions targeted were not consistent across studies. Bioinformatics pipelines, including sequence filtering, chimera removal, OTU clustering, and taxonomic assignment, varied widely across studies. A detailed overview is provided in Supplementary Table S2.

Alpha diversity (Fig. 2) was reported by five studies (Akiyama *et al.*, 2019; Ata *et al.*, 2019; Huang *et al.*, 2021; Chang *et al.*, 2022; Yang *et al.*, 2023), but only three (Akiyama *et al.*, 2019; Chang *et al.*, 2022; Yang *et al.*, 2023) found statistically significant differences in bacterial diversity between cases and controls.

Beta diversity was evaluated in six studies (Campos *et al.*, 2018; Akiyama *et al.*, 2019; Ata *et al.*, 2019; Huang *et al.*, 2021; Chang *et al.*, 2022; Yang *et al.*, 2023), but only two (Campos *et al.*, 2018; Chang *et al.*, 2022) reported a statistically significant difference in microbial composition between women with and without endometriosis.

Microbiome composition at the genus level was reported in all studies (Fig. 4B). Only four genera were consistently identified in two or more studies as being more abundant in endometriosis, which were *Bifidobacterium* sp. (Chang *et al.*, 2022; Yang *et al.*, 2023), *Pseudomonas* sp. (Akiyama *et al.*, 2019; Wei *et al.*, 2020), *Streptococcus* sp. (Akiyama *et al.*, 2019; Ata *et al.*, 2019; Chang *et al.*, 2022; Yang *et al.*, 2023), and *Veillonella* sp. (Wei *et al.*, 2020; Yang *et al.*, 2023). *Megasphaera* sp.*, Prevotella* sp.*, Sneathia* sp. which were found to be more abundant in endometriosis cases in Yang *et al.* (2023), were more abundant in controls in Ata *et al.* (2019). Three studies (Akiyama *et al.*, 2019; Wei *et al.*, 2020; Huang *et al.*, 2021) also reported higher-level taxonomic data at the phylum, class, order, and/or family level (Supplementary Table S3).

Furthermore, among studies that reported significant differences at the genus level between women with and without endometriosis, two (Akiyama *et al.*, 2019; Wei *et al.*, 2020) were considered to be of moderate quality according to the NOS, while the other three (Ata *et al.*, 2019; Chang *et al.*, 2022; Yang *et al.*, 2023) were assessed as high quality, indicating a low risk of bias (Supplementary Table S4).

#### Peritoneal fluid

Technical characteristics of the eight studies (Campos *et al.*, 2018; Wang *et al.*, 2018; Wei *et al.*, 2020; Huang *et al.*, 2021; Lee *et al.*, 2021; Yuan *et al.*, 2022; Zhu *et al.*, 2024; Malvezzi *et al.*, 2025) investigating the microbiome of peritoneal fluid in women with endometriosis are detailed in Table 5.

The number of endometriosis cases ranged from n = 21 to n = 55. Control groups included n = 14 to n = 45 participants, composed exclusively of women undergoing laparoscopy for gynecological conditions unrelated to endometriosis. In two studies (Wang *et al.*, 2018; Zhu *et al.*, 2024), controls also included infertile women. Most studies reported comparable age between groups; however, one study (Malvezzi *et al.*, 2025) observed a statistically significant age difference (*P* = 0.029). Two studies (Campos *et al.*, 2018; Wei *et al.*, 2020) did not report age data. BMI was significantly different in one study (Zhu *et al.*, 2024), unreported in two (Campos *et al.*, 2018; Wei *et al.*, 2020), and comparable across groups in the remaining studies.

The diagnosis of endometriosis was confirmed by surgery and histological examination in all studies, except for one (Wang *et al.*, 2018), which relied on surgical findings alone. An unusual methodological choice was made by Lee *et al.* (2021), who analyzed the microbiome in extracellular vesicles isolated from peritoneal fluid, rather than the fluid itself. The phenotypes of endometriosis investigated varied widely across studies, although all the studies included severe presentations of the disease.

Regarding hormonal therapy, almost all studies considered it an exclusion criterion, except for Malvezzi *et al.* (2025) and Campos *et al.* (2018), who did not even report whether its distribution differed significantly between groups. Similarly, while most studies excluded participants with recent antibiotic use, two (Wang *et al.*, 2018; Malvezzi *et al.*, 2025) did not report data on this aspect.

Significant heterogeneity was observed in the menstrual cycle phase during which peritoneal fluid samples were collected. Some studies collected samples exclusively during the early follicular phase (Wang *et al.*, 2018; Wei *et al.*, 2020); while one study (Lee *et al.*, 2021) collected exclusively during the follicular phase. One study (Zhu *et al.*, 2024) did not report the timing of sample collection. Other studies collected samples at various phases of the menstrual cycle. Among them, three studies (Campos *et al.*, 2018; Yuan *et al.*, 2022; Malvezzi *et al.*, 2025) reported that the distribution of cycle phases was similar between study groups. In contrast, two studies (Huang *et al.*, 2021; Zhu *et al.*, 2024) did not indicate whether the phase distribution was comparable between cases and controls. No study accounted for dietary habits as an exclusion criterion.

Microbiome sequencing methodologies are summarized in Table 5. All studies used 16S rRNA gene sequencing via Illumina or Ion Torrent platforms, except for one (Campos *et al.*, 2018), who used qRT-PCR to target specific bacterial species. However, the 16S regions amplified differed between studies, and there was no standardized approach to bioinformatic processing. Details on pipelines used for filtering, chimeric sequence removal, identification of OTUs, etc, varied significantly. Notably, some studies provided minimal (Wei *et al.*, 2020; Zhu *et al.*, 2024) or no (Wang *et al.*, 2018) bioinformatics details (Supplementary Table S2).

Alpha diversity (Fig. 2) was assessed in four studies (Huang *et al.*, 2021; Lee *et al.*, 2021; Yuan *et al.*, 2022; Zhu *et al.*, 2024). Only one (Zhu *et al.*, 2024) reported a significant difference, observed only between women with stage III-IV endometriosis and controls.

Beta diversity was reported by five studies (Campos *et al.*, 2018; Huang *et al.*, 2021; Lee *et al.*, 2021; Yuan *et al.*, 2022; Zhu *et al.*, 2024). Significant differences in microbial community structure between endometriosis and control groups were consistently found in three studies (Campos *et al.*, 2018; Lee *et al.*, 2021; Yuan *et al.*, 2022), and in one study (Zhu *et al.*, 2024) only when comparing stage III-IV patients with both stage I-II and controls combined.

**Figure 5.**
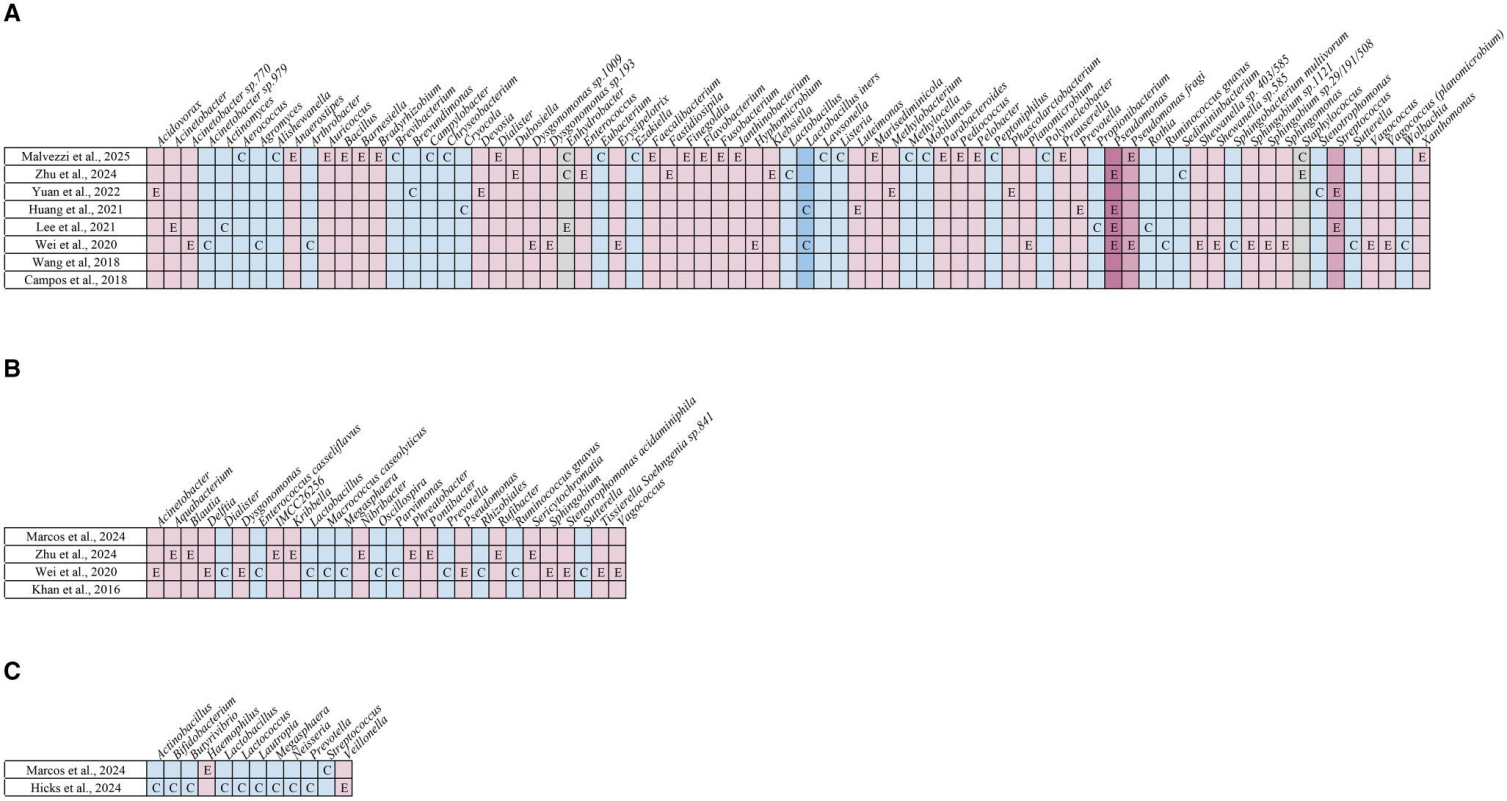
**Bacterial genera identified across peritoneal, uterine, and oropharyngeal fluid samples in the included studies.** E, genus’s abundance increased in endometriosis; C, genus’s abundance increased in controls; dark pink, increased in endometriosis in ≥3 studies; mid-pink, increased in endometriosis in two studies; light pink, increased in endometriosis in one study; grey, inconsistent findings across studies; light blue, decreased in endometriosis in one study; mid-blue, decreased in endometriosis in two studies. (**A**) Peritoneal fluid. (**B**) Uterine fluid. (**C**) Oropharyngeal fluid.

Considering the genera identified in the peritoneal fluid samples, significant differences in microbial composition were observed between women with and without endometriosis. Notably, an increased abundance of *Streptococcus* sp. was found in the endometriosis groups in two studies (Lee *et al.*, 2021; Yuan *et al.*, 2022). *Pseudomonas* was another genus consistently reported with higher abundance in endometriosis cases across multiple studies (Wei *et al.*, 2020; Huang *et al.*, 2021; Lee *et al.*, 2021; Zhu *et al.*, 2024; Malvezzi *et al.*, 2025), emerging as a predominant and recurring finding. This latter association was further supported by unsupervised analyses using random forest classifiers (Huang *et al.*, 2021). In contrast, *Lactobacillus iners* was reported to be more abundant in the control groups in two studies (Wei *et al.*, 2020; Huang *et al.*, 2021; Fig. 5A), suggesting a potential protective or non-pathogenic role.

Despite these consistent findings, notable inconsistencies were also observed. For instance, *Enhydrobacter* species (Lee *et al.*, 2021; Zhu *et al.*, 2024; Malvezzi *et al.*, 2025) and *Staphylococcus* sp. (Zhu *et al.*, 2024; Malvezzi *et al.*, 2025) were found to be more abundant in endometriosis cases in some studies, whereas other investigations reported higher levels in the control group. These divergent results may reflect methodological differences or population-specific factors across studies.

In addition to genus-level analysis, several studies also provided bacterial identification at higher taxonomic levels, including phylum, class, order, and family (Supplementary Table S3).

Furthermore, among studies that reported significant differences at the genus level between women with and without endometriosis, four (Wei *et al.*, 2020; Huang *et al.*, 2021; Yuan *et al.*, 2022; Zhu *et al.*, 2024) were considered to be of moderate quality according to the NOS. The other two (Lee *et al.*, 2021; Malvezzi *et al.*, 2025) were assessed as high quality, indicating a low risk of bias (Supplementary Table S4).

#### Uterine and endometrial fluid

Technical characteristics of the four studies (Khan *et al.*, 2016; Wei *et al.*, 2020; Marcos *et al.*, 2024; Zhu *et al.*, 2024) analyzing the uterine fluid microbiome in women with endometriosis are detailed in Table 6.

The sample sizes across these investigations were generally small, ranging from as few as n = 8 to a maximum of n = 36 cases, and from n = 14 to n = 32 controls (Table 6). In all studies, the control groups comprised women with other gynecological conditions. Age was reported to be comparable between groups in two studies (Marcos *et al.*, 2024; Zhu *et al.*, 2024), whereas one study (Khan *et al.*, 2016) did not clarify whether age distributions were similar, and another (Wei *et al.*, 2020) did not provide age data at all. BMI was reported only by one study (Zhu *et al.*, 2024), which found a statistically significant difference between groups (*P* = 0.007).

Endometriosis diagnosis was confirmed by surgery and histology in most of the studies, all of which included patients across different disease stages. Notably, the most recent study (Marcos *et al.*, 2024) based the diagnosis on imaging or surgical findings but did not report even the disease stage.

Regarding hormonal treatment, there was considerable variation. In two studies (Wei *et al.*, 2020; Zhu *et al.*, 2024), hormonal treatments were an exclusion criterion. In contrast, in one (Khan *et al.*, 2016), half of the participants in each group were receiving GnRH analogues as part of the study design. Marcos *et al.* (2024) did not report whether hormonal therapy was an exclusion criterion or whether its use differed between groups. Most studies excluded participants with recent antibiotic use; however, one study (Khan *et al.*, 2016) did not report any information on this aspect.

There was also substantial heterogeneity regarding the menstrual cycle phase during which uterine fluid samples were collected. Marcos *et al.* (2024) collected samples during ovulation (Days 12–16 of the menstrual cycle), Wei *et al.* (2020) exclusively during the early follicular phase, while Zhu *et al.* (2024) did not report the timing of sample collection. In Khan *et al.* (2016), samples were collected across different phases of the cycle, but it was not specified whether this variability was distributed similarly between groups. Consistent with the majority of studies included in this review, none of the investigations evaluating the uterine fluid microbiome considered special dietary habits as an exclusion criterion.

Microbiome analysis details are presented in Table 6. All studies employed 16S rRNA sequencing using Illumina or Ion Torrent platforms; however, the amplified regions varied (Wei *et al.*, 2020; Zhu *et al.*, 2024), or were not reported at all (Khan *et al.*, 2016; Marcos *et al.*, 2024). Likewise, comprehensive details regarding bioinformatic analyses, such as pipelines used, filtering criteria, chimeric sequence removal, identification of OTUs, etc, were absent in most studies, with the exception of Marcos *et al.* (2024), who reported nearly all steps of the computational workflow (Supplementary Table S2).

Only one study (Zhu *et al.*, 2024) assessed microbial diversity measures. In this study, alpha diversity was found to be similar between groups, while beta diversity differed significantly between women with stages III-IV endometriosis and those with stages I-II combined with controls (Fig. 2). Regarding the taxonomic composition, only two studies (Wei *et al.*, 2020; Zhu *et al.*, 2024) reported the identification of bacterial genera in uterine or endometrial fluid samples. However, no specific taxa were consistently observed between these two studies (Fig. 5B).

#### Ovarian cyst fluid

The microbiome of ovarian cyst fluid in women with endometriosis has been examined in only a single study to date (Khan *et al.*, 2016), as detailed in Table 1. This study included a limited sample size, comprising just n = 8 cases and n = 8 controls (Table 7). Information regarding patient selection, sample collection, as well as bioinformatic methods and pipelines, is summarized in Tables 1 and 7 and Supplementary Table S2.

Notably, the study did not evaluate alpha or beta diversity metrics (Fig. 2). At the family level, ovarian cyst fluid from endometriosis patients showed an enrichment of *Streptococcaceae* and *Moraxellaceae*, alongside a relative decrease of *Lactobacillaceae*, *Enterobacteriaceae*, and *Staphylococcaceae*, when compared to cyst fluid from women with other benign ovarian conditions (Supplementary Table S3). These findings, while suggestive, remain preliminary and underscore the need for further confirmatory studies with larger cohorts and standardized methodologies.

#### Oropharyngeal fluid

The microbiome of oropharyngeal fluid in women with endometriosis has been investigated by only two studies to date (Marcos *et al.*, 2024; Hicks *et al.*, 2025), with technical characteristics summarized in Table 8. Marcos *et al.* (2024) included a small sample of n = 8 endometriosis cases and n = 13 infertile women without endometriosis as controls, while Hicks *et al.* (2025) evaluated a larger cohort comprising n = 21 women with endometriosis, n = 24 women with other gynecological conditions, and n = 19 healthy controls. As observed in other microbiome studies included in this review, critical variables that could influence microbial composition, such as hormonal use and dietary patterns, were not considered exclusion criteria in either study. Moreover, BMI was not reported, and Hicks *et al.* (2025) did not provide information on the timing of sample collection within the menstrual cycle or on the distribution of recent antibiotic use across groups.

Microbiome sequencing methods are detailed in Table 8, while the approaches to bioinformatic analyses, including pipelines, filtering, chimeric sequence removal, identification of OTUs, etc, varied widely and are presented in Supplementary Table S2. Of the two studies, only Hicks *et al.* (2025) evaluated alpha and beta diversity. While alpha diversity did not differ significantly between groups, beta diversity was found to be statistically different between them (Fig. 2).

Regarding microbial composition, certain taxa appeared to differ between women with and without endometriosis. *Haemophilus* sp. (Marcos *et al.*, 2024) and *Veillonella* sp. (Hicks *et al.*, 2025) were reported in greater abundance in the oropharyngeal fluid of women with endometriosis. Conversely, several genera, including *Actinobacillus* sp.*, Bifidobacterium* sp.*, Butyrivibrio* sp.*, Lactobacillus* sp.*, Lactococcus* sp.*, Lautropia* sp.*, Megasphaera* sp.*, Neisseria* sp.*, Prevotella* sp. (Hicks *et al.*, 2025), as well as *Streptococcus* sp. (Marcos *et al.*, 2024) were found to be more abundant in controls (Fig. 5C). At the family level (Supplementary Table S3), endometriosis cases were associated with increased abundance of *Actinomycetaceae* (Hicks *et al.*, 2025) and *Enterobacteriaceae* (Marcos *et al.*, 2024), along with a reduction in *Peptostreptococcaceae* and *Neisseriaceae* (Hicks *et al.*, 2025). Despite these findings, inconsistencies across studies highlight the need for further research to confirm whether these microbial alterations are reproducible and relevant to the pathophysiology of endometriosis (Supplementary Table S3).

### Microbiome detection in tissues

#### Microbiome detection in eutopic endometrium

The microbiome of eutopic endometrial tissue in women with endometriosis has been investigated in six studies (Hernandes *et al.*, 2020; Khan *et al.*, 2021; Wessels *et al.*, 2021; Muraoka *et al.*, 2023; Marcos *et al.*, 2024; Guo *et al.*, 2025), with technical characteristics summarized in Table 9.

**Table 9.** Technical characteristics of microbiome analyses across eutopic endometrium samples in the included studies.

Authors (year)	**Study groups** (cases vs controls)	**Number of samples included in the analysis** (cases vs controls)	**Age (years)** (cases vs controls)	**BMI (kg/m^2^)** (cases vs controls)	Sampling technique	**Extraction kit—brand** *(its indication)*	Method	Sequencing platform	Sequencing instrument	Region amplified	Reads length
Guo *et al.* (2025)	Symptomatic women with endometriosis vs symptomatic women without endometriosis	30 vs 13	37.10 ± 7.30 vs 40.92 ± 7.41 ^§^ (*P*=NS)	21.27 ± 1.94 vs 23.63 ± 3.37 ^§^ (*P* < 0.01)	‘*Prior to surgery, all patients had their vaginas swabbed with chlorhexidine to prepare for gynecological laparoscopy. A sterile vaginal speculum was inserted, followed by passing a double-sheathed, sterile Pipelle endometrial suction curette (Cooper Surgical, Trumbull, CT, USA) through the cervix to collect endometrial biopsy samples. Great care was taken to avoid contact with the vaginal wall and cervix. The biopsy samples were placed in sterile 15 ml Falcon conical tubes (polystyrene) (Fisher Scientific, Ottawa, ON, Canada) and transported to the laboratory on ice for processing within 30 min.*’	QIAamp DNA Kit—Qiagen *(For isolation of genomic, mitochondrial, bacterial, parasite or viral DNA from tissues, swabs, CSF, blood, body fluids or washed cells from urine)*	16S	Illumina	NovaSeq	V3-V4	NR
Marcos *et al.* (2024)	Infertile women with endometriosis vs women with other infertility-related conditions	8 vs 13	42.7 ± 5.5 vs 39.4 ± 3.7 ^§^ (*P*=NS)	NR	‘*[…] a flexible sterile cannula or catheter was inserted through the cervix under ultrasound guidance into the uterus. Once in contact with the uterine wall, a suction was performed to biopsy the endometrial tissue. It is important not to perform any suction until you are sure that you have reached the endometrial wall and not to suction immediately after obtaining the tissue to avoid contamination of the sample with other fluids.*’	QIAamp Fast DNA Tissue Kit—Qiagen *(For rapid isolation of genomic DNA from solid tissue samples)*	16S	Ion Torrent	PGM	NR	Single-end
Muraoka *et al.* (2023)	Women with endometriosis vs women with other gynecological conditions	42 vs 42	34.5 [31.0–39.0] vs 34.5 [32.0–37.0] ^†^ (*P*=NR)	NR	‘*Primary endometrial samples were taken from […] surgical removal of the uterus together with the endometriotic lesions.*’	NR	qRT-PCR	NR	*Primers for F. nucleatum.*		
Wessels *et al.* (2021)	Women with pelvic pain with endometriosis vs women with pelvic pain without endometriosis	12 vs 9	33.8 ± 5.8 vs 35.1 ± 3.3 ^§^ (*P*=NS)	NR	‘*Immediately before surgery the vagina was swabbed with chlorhexidine… a double sheathed, sterile pipelle endometrial suction curette (Cooper Surgical, Trumbull, CT, USA) was passed through the cervix to collect an endometrial biopsy, taking care to avoid contact with the vaginal wall and cervix.*’	RNeasy Kit—Qiagen *(For purification of total RNA from cells, tissues, and yeast)*	16S	Illumina	MiSeq	V3	300 bp
Khan *et al.* (2021)	(Women with endometriosis receiving different treatments: untreated vs GnRHa vs LVFX vs GnRHa+ LVFX) vs (fertile women with uterine fibroids receiving different treatments: untreated vs GnRHa vs LVFX vs GnRHa+ LVFX)	(21 vs 11 vs 15 vs 6) vs (11 vs 12 vs 10 vs 14) (*P*=NR)	(36.3 ± 7.7 vs 38.7 ± 5.2 vs 38.2 ± 8.2 vs 35.5 ± 5.6) vs (41.2 ± 8.1 vs 37.5 ± 5.3 vs 43.0 ± 4.5 vs 36.7 ± 4.5) ^§^ (*P*=NR)	NR	‘*Endometrial samples were collected […] during laparoscopy.*’	UltraClean^®^ Soil DNA Isolation Kit—MoBio *(For isolate cellular, PCR quality DNA from soil)*	16S	Illumina	Miseq	V5–V6	NR
Hernandes *et al.* (2020)	Women with endometriosis vs women without endometriosis with other benign gynecological conditions	10 vs 11	NR	NR	‘*Eutopic endometrium […] samples were collected by curettage […]….every care was taken to prevent the swab from touching other tissues adjacent to the target site, not having contact with blood, or any other instrument.*’	DNeasy PowerSoil Pro Kit—Qiagen *(For the isolation of microbial genomic DNA from all soil types)*	16S	Illumina	MiSeq	V3–V4	300 nt single-end

Data reported as reported by the original papers, unless otherwise stated.

Bp, base pairs; NR, not reported; NS, non-significant; nt, nucleotides; LVFX, levofloxacin; qRT-PCR, quantitative real time-PCR.

§ Data are expressed as mean±SD.

† Data are expressed as median [25th–75th percentile].

Sample sizes varied considerably, ranging from as few as n=8 to a maximum of n=53 endometriosis cases, with one study further subdividing its few cases (n=53) based on treatment exposure, including GnRH analogues or levofloxacin. Control groups, generally composed of symptomatic or infertile women, or those with other gynecological conditions, ranged from n=9 to n=51 participants, with similar subgrouping applied in the latter.

Only half of the studies reported comparisons of age between cases and controls. One study (Hernandes *et al.*, 2020) did not provide age-related data, while two (Khan *et al.*, 2021; Muraoka *et al.*, 2023) reported ages without clarifying whether groups were statistically comparable. BMI was reported in only one study, which found a statistically significant difference between groups.

The diagnostic criteria for endometriosis were relatively consistent across studies, with most confirming diagnosis through both surgery and histological examination. While two studies (Khan *et al.*, 2021; Muraoka *et al.*, 2023) focused exclusively on ovarian endometriosis and one (Hernandes *et al.*, 2020) on deep disease, the remaining did not specify disease phenotype. Only one (Wessels *et al.*, 2021) reported that all the stages of endometriosis were included.

The use of hormonal treatment varied. In three studies (Wessels *et al.*, 2021; Muraoka *et al.*, 2023; Guo *et al.*, 2025), its use was an exclusion criterion. In contrast, in Khan *et al.* (2021), hormonal treatment with GnRH analogue was integrated into the study design, with participants evenly distributed across treatment groups. Marcos *et al.* (2024) did not clarify the compatibility of treatment usage between groups, and Hernandes *et al.* (2020) omitted this information entirely. Regarding use of antibiotics, it was excluded in nearly all studies, except one (Wessels *et al.*, 2021), who did not report it as an exclusion criterion, and another (Khan *et al.*, 2021), who incorporated levofloxacin use as part of the intervention. As with other sample types, none of the studies considered dietary habits as an exclusion criterion.

Despite the known influence of hormonal cycles on endometrial physiology and microbial communities, the menstrual cycle phase at the time of sample collection was inconsistently addressed. Marcos *et al.* (2024) were the only authors to collect all the samples during a uniform menstrual phase, the time of ovulation. Muraoka *et al.* (2023) and Wessels *et al.* (2021) collected samples at different phases but reported that time did not differ significantly between groups. Khan *et al.* (2021) documented cycle phases but did not report on their statistical comparability. Guo *et al.* (2025), and Hernandes *et al.* (2020) did not mention the menstrual phase at all.

The sampling methods also varied across studies. Curettage was used in three studies (Hernandes *et al.*, 2020; Wessels *et al.*, 2021; Guo *et al.*, 2025), a seed swab in Khan *et al.* (2021), and an endometrial sampler in Marcos *et al.* (2024). In the study by Muraoka *et al.* (2023), samples were obtained during surgical removal of the uterus, although the methodology was not described in detail.

Methodological heterogeneity was also reflected in DNA extraction protocols. Some studies employed kits originally intended for soil samples (such as UltraClean^®^ Soil DNA Isolation Kit—MoBio (Khan *et al.*, 2021) and DNeasy PowerSoil Pro Kit—Qiagen (Hernandes *et al.*, 2020)), raising concerns about methodological appropriateness for tissue microbiome profiling. Sequencing platforms were consistent, with nearly all studies employing 16S rRNA sequencing on Illumina or Ion Torrent systems; however, the regions of the 16S gene analyzed varied widely, ranging from the V3 region alone (Wessels *et al.*, 2021) to V3–V4 (Hernandes *et al.*, 2020; Guo *et al.*, 2025) and V5–V6 (Khan *et al.*, 2021), while Marcos *et al.* (2024) did not specify the region amplified. Muraoka *et al.* (2023) took a distinct approach, conducting a bioinformatic reanalysis of publicly available datasets (European Nucleotide Archive studies PRJEB16013 and PRJEB21098), followed by targeted qRT-PCR validation. Bioinformatic analyses, including sequence filtering, chimera removal, and OTU assignment, varied across studies, with further details presented in Supplementary Table S2.

Alpha diversity findings were inconsistent (Fig. 2). While Hernandes *et al.* (2020) reported no differences in microbial richness or evenness between women with and without endometriosis, Guo *et al.* (2025), Wessels *et al.* (2021), and Khan *et al.* (2021) observed significant differences. Khan *et al.* (2021) additionally found that alpha diversity varied among treated and untreated endometriosis patients.

Beta diversity analyses yielded similarly inconsistent results. Three studies (Hernandes *et al.*, 2020; Khan *et al.*, 2021; Wessels *et al.*, 2021) found no significant differences between cases and controls. In contrast, Guo *et al.* (2025) observed a significant separation in microbial community structure between the groups. Notably, Marcos *et al.* (2024) and Muraoka *et al.* (2023) did not report any diversity metrics for eutopic endometrium tissue in their analyses.

**Figure 6.**
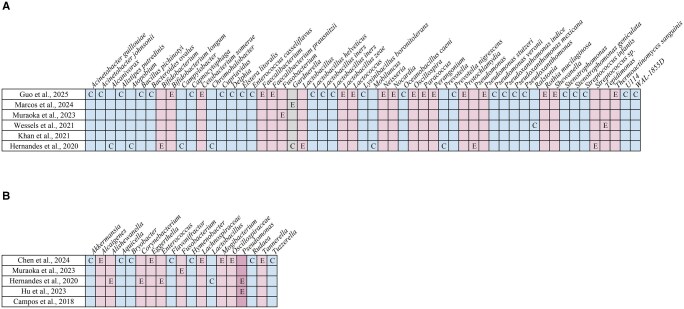
**Bacterial genera identified across tissue sample types in the included studies.** E, genus’s abundance increased in endometriosis; C, genus’s abundance increased in controls; mid-pink, increased in endometriosis in two studies; light pink, increased in endometriosis in one study; grey, inconsistent findings across studies; light blue, decreased in endometriosis in one study. (**A**) Eutopic endometrium. (**B**) Endometriotic tissue.

In terms of taxonomic composition, the genera identified as differing significantly between women with and without endometriosis were not consistent across studies (Fig. 6A). No bacterial genus was consistently found to be significantly altered across studies, highlighting the lack of reproducibility and the overall weakness of evidence for an endometriosis-associated microbial signature in eutopic endometrial tissue. These inconsistencies underscore the challenges in identifying a coherent dysbiosis profile for endometriosis. Three studies (Wessels *et al.*, 2021; Marcos *et al.*, 2024; Guo *et al.*, 2025) extended their analyses to higher taxonomic levels, including phylum, class, order, and family (Supplementary Table S3).

#### Microbiome detection in endometriotic tissues

Technical characteristics of the five studies (Campos *et al.*, 2018; Hernandes *et al.*, 2020; Hu *et al.*, 2023; Muraoka *et al.*, 2023; Chen *et al.*, 2024) that analyzed the microbiome composition of ectopic endometrial tissue compared to control tissues are detailed in Table 10.

**Table 10.** Technical characteristics of microbiome analyses across endometriotic tissue samples in the included studies.

Authors (year)	**Study groups** (cases vs controls)	**Number of samples included in the analysis** (cases vs controls)	**Age (years)** (cases vs controls)	**BMI (kg/m2)** (cases vs controls)	Sampling technique	**Extraction kit—brand** *(its indication)*	Method	Sequencing platform	Sequencing instrument	Region amplified	Reads length
Chen *et al.* (2024)	Ovarian endometriotic tissue from women with endometriosis vs eutopic endometrium from non-endometriosis women with uterine fibroids	23 vs 22	34.8 ± 6.8 vs 37.2 ± 8.2 ^§^ (*P*=NS)	NR	‘*Samples were collected in sterile centrifuge tube on ice*’ during laparoscopic surgery.’	MagPure Soil DNA Kit—Magen *(For isolation of high-quality genomic DNA from various soil, stool, and other environmental samples)*	16S	Illumina	NovaSeq 6000	V3–V4	250 bp paired-end
Hu *et al.* (2023)	Endometriotic tissue vs eutopic endometrium from the same women with endometriosis	14 vs 14	–	–	‘*Ectopic and eutopic endometrial tissues were collected intraoperatively. After disinfecting the cervical canal and vagina with iodine, a vacuum suction tube was inserted into the uterine cavity to collect ectopic endometrial tissues, during which time the tube was not in contact with the vaginal wall or cervix.*’	NR	16S	Illumina	NovaSeq	V3–V4	250 bp paired-end
Muraoka *et al.* (2023)	Ovarian endometriotic tissue from women with endometriosis vs eutopic endometrium from the same women	42 vs 42	–	–	‘*Paired primary endometrial and endometriosis samples were taken […] during surgical removal of the uterus together with the endometriotic lesions.*’	NR	qRT-PCR	NR	*Primers for F. nucleatum.*		
Hernandes *et al.* (2020)	Endometriotic tissue vs eutopic endometrium from the same women with endometriosis	10 vs 11	–	–	‘*Eutopic endometrium and endometriotic lesion tissue samples were collected by curettage and laparoscopic surgery, respectively. All samples were collected at the operative room to minimize contamination and every care was taken to prevent the swab from touching other tissues adjacent to the target site, not having contact with blood, or any other instrument.*’	DNeasy PowerSoil Pro Kit—Qiagen *(For the isolation of microbial genomic DNA from all soil types)*	16S	Illumina	MiSeq	V3–V4	300 nt single-end
Campos *et al.* (2018)	Endometriotic tissue vs healthy peritoneum from women without endometriosis	68 vs 30	NR	NR	‘*Biopsy tissue samples were collected during laparoscopy*’	PureLink Genomic DNA Mini Kit—Invitrogen *(For genomic DNA purification from blood, tissues, cells, bacteria, swabs, and blood spots)*	qRT-PCR	PCR Array kit (Qiagen-SABioscience)	‘*Nucleic acid extracts were tested against M. hominis, M. genitalium, Ureaplasma urealyticum, and U. parvum.*’		

Data reported as reported by the original papers, unless otherwise stated.

Bp, base pairs; NA, not applicable; NR, not reported; NS, non-significant; nt, nucleotides; qRT-PCR, quantitative real time-PCR.

§ Data are expressed as mean±SD.

Sample sizes ranged from n=10 to n=68 for endometriosis cases and from n=11 to n=42 controls. When evaluating the microbiome of endometriotic tissues, the choice of control tissue is critical. Microbial profiles are expected to differ depending on whether the comparison is made to the eutopic endometrium from the same affected women, from a woman without endometriosis, or from a different tissue altogether. In three studies (Hernandes *et al.*, 2020; Hu *et al.*, 2023; Muraoka *et al.*, 2023), ectopic tissue microbiomes were compared to eutopic endometrial tissue from the same woman. In contrast, Chen *et al.* (2024) used eutopic endometrium from women without endometriosis but with uterine fibroids as the control tissue. These control subjects were age-matched to the cases, although BMI data were not reported. Similarly, Campos *et al.* (2018) used peritoneal tissue from symptomatic women without endometriosis as controls, but data on age and BMI were not reported. In all studies, both eutopic and ectopic tissue samples were obtained intraoperatively (Table 1).

Histological confirmation of endometriosis was conducted in all studies except one (Muraoka *et al.*, 2023), which relied on surgical identification alone (Table 10). Three studies (Hu *et al.*, 2023; Muraoka *et al.*, 2023; Chen *et al.*, 2024) focused exclusively on ovarian endometriosis, while Hernandes *et al.* (2020) analyzed deep endometriosis. Campos *et al.* (2018) included multiple phenotypes and stages of the disease.

Hormonal treatment varied across the studies. In three studies (Hu *et al.*, 2023; Muraoka *et al.*, 2023; Chen *et al.*, 2024), participants were not on hormonal therapies. Hernandes *et al.* (2020) did not report hormonal use and did not list it as an exclusion criterion. Campos *et al.* (2018) included participants with varying hormonal use across groups but did not indicate whether differences were statistically significant.

Menstrual cycle phase at the time of sample collection was not consistently controlled. Hernandes *et al.* (2020) did not report this information. Campos *et al.* (2018) noted similar phases between groups, while Chen *et al.* (2024) stated that over 90% of samples were in the follicular phase. Regarding antibiotic use, most studies considered it an exclusion criterion, except for Chen *et al.* (2024), who did not specify its distribution between groups. None of the studies reported any exclusion criteria regarding dietary habits.

Technical aspects and microbiome analysis methods are summarized in Table 10. Only Campos *et al.* (2018) reported using a tissue-specific DNA extraction kit (Purelink Genomic DNA Mini Kit, Invitrogen). Other studies used adapted soil DNA extraction kits (Hernandes *et al.*, 2020; Chen *et al.*, 2024), and two (Hu *et al.*, 2023; Muraoka *et al.*, 2023) did not report their extraction methods. Three studies used 16S rRNA sequencing on Illumina platforms, targeting the V3-V4 regions. Muraoka *et al.* (2023) and Campos *et al.* (2018) did not conduct 16S sequencing but instead used qRT-PCR to detect specific taxa. As previously mentioned, Muraoka *et al.* (2023) conducted a bioinformatic reanalysis of publicly available datasets followed by targeted qRT-PCR validation. Bioinformatic analyses, including sequence filtering, chimera removal, and OTU assignment, varied across studies, with further details presented in Supplementary Table S2.

Alpha and beta diversity were evaluated by three studies (Hernandes *et al.*, 2020; Hu *et al.*, 2023; Chen *et al.*, 2024) (Fig. 2). All studies reported no significant differences in alpha diversity between ectopic and control tissues. Similarly, beta diversity did not differ between groups, except in the study by Hernandes *et al.* (2020), which reported significant differences when using weighted UniFrac distances, but not when using Bray–Curtis dissimilarity.

Regarding the taxonomic composition, the results were heterogeneous. Among the genera identified as significantly differing between ectopic and control tissues (Fig. 6B), only *Pseudomonas* was reported by more than one study (Hernandes *et al.*, 2020; Hu *et al.*, 2023) as being significantly increased in ectopic tissue compared to eutopic tissue. This limited concordance reflects the weak evidence base, lack of reproducibility, and the absence of evidence of a distinct microbial signature in endometriotic lesions.

Furthermore, the two studies (Hernandes *et al.*, 2020; Hu *et al.*, 2023) that reported significant differences at the genus level between women with and without endometriosis were assessed as being of moderate quality according to the NOS, indicating a moderate risk of bias (Supplementary Table S4).

## Discussion

The association between endometriosis and microbiota is emerging as a promising area of research for understanding pathophysiological mechanisms underlying the disease and for identifying potential biomarkers for diagnosis and management. A healthy microbial balance across different anatomical sites is essential for maintaining mucosal integrity, protecting against pathogens, and regulating physiological processes. Dysbiosis, or an imbalance in the microbiota, can compromise the intestinal barrier, allowing bacteria or endotoxins to pass through, triggering inflammation responses that may contribute to the onset or progression of various diseases. Additionally, some gut bacteria produce enzymes involved in estrogen metabolism, potentially influencing the estrobolome, the collection of microbial genes involved in estrogen metabolism and estrogen-related disorders such as endometriosis (Pai *et al.*, 2023). In line with these concepts, a quite large number of narrative reviews have been published over the past few years, speculating on the causal relationship between microbiota and endometriosis (Leonardi *et al.*, 2020; D’Alterio *et al.*, 2021; Colonetti *et al.*, 2023; Weber *et al.*, 2024). Yet, despite these efforts, there remains a critical gap in the literature including a thorough review of study designs and methodologies employed to investigate microbiota alterations in endometriosis.

Scoping reviews are particularly valuable in such contexts, enabling researchers to map existing evidence systematically and to discern patterns across studies. Indeed, several studies have utilized scoping reviews to synthesize knowledge in various areas of microbiome research (Gáspár *et al.*, 2024; Lianos *et al.*, 2024; Nagamine, 2024; Sessa *et al.*, 2025). A scoping review is often more suitable for a microbiome research compared to a systematic review due to several reasons: (i) it allows a broad overview of available evidence, including different approaches, trends, and knowledge gaps, rather than answering a specific, narrow question like a systematic review; (ii) contrary to a systematic review which requires strict inclusion/exclusion criteria, it embraces a wider diversity among studies; (iii) it is more appropriate if there is not enough homogeneous data to conduct a quantitative synthesis; microbiome studies vary widely in sample types, sequencing platform, analytical pipelines, and study designs (cross-sectional, longitudinal, interventional). Importantly in our context, scoping reviews also illuminate methodological shortcomings and highlight critical areas where further investigation is needed. Such reviews are particularly well-suited for identifying research gaps and shaping future research agendas, offering a roadmap for addressing limitations and improving the rigor of studies in our field.

### Main findings

Recently, multiple studies have focused on characterizing a distinct endometriosis microbiome signature in both humans and animal models. As shown in the results section of this review, several bacterial species have been identified as being associated with endometriosis pathogenesis. However, consistency in the type of bacterial species present and their relative abundances compared to control subjects continues to be elusive for all the anatomical districts investigated. In eutopic endometrial tissue investigated by six studies, no bacterial genus was consistently found to be significantly different between groups across the studies. Some consistency was found for the reduced presence of *Lachnospira* sp. in stool/anal fluid of endometriosis patients according to 4 of the 15 studies investigating this district microbiome and for the higher presence of *Streptococcus* sp. in four of the seven studies on cervical samples. Similarly, a higher abundance in peritoneal fluid of patients with endometriosis was found for *Pseudomonas* sp. in four of the eight studies. Among the genera reported as significantly differing between women with and without endometriosis in vaginal microbiome, three genera (*Alloscardovia* sp.*, Escherichia* sp., and *Veillonella* sp.) were identified as more abundant in endometriosis in 3 of the 15 studies. Several studies even reported contradicting results between women affected and controls, specifically with regard to abundance of *Blautia* sp. and *Ruminococcus* sp. in the stool microbiome and *Gardnerella* sp., *Prevotella* sp., and *Sneathia* sp. in the vaginal district. As shown in Fig. 2, inconsistencies across studies were also found for alpha and beta diversity for all the districts investigated. Overall, these data hint at weak evidence supporting an endometriosis-associated microbial signature.

### Shortcomings and critical areas

#### The challenge of sample size

One recurring issue in microbiome research is the challenge of ensuring adequate sample sizes. Studies have suggested that statistical analyses of microbiome datasets require specialized considerations that differ from traditional sample size calculation methods (Ferdous *et al.*, 2022), since these studies are exposed to high risks of type I errors. Thus, in the context of case-control studies investigating differences in microbial composition, robust study design requires an *a priori* assessment of sample size to ensure sufficient statistical power to detect hypothesized effects.

Recent guidelines aiming to improve the quality and consistency of microbiome research underscore the importance of power calculations and adequate sample sizes as essential criteria for rigorous study design (Kelly *et al.*, 2015). Despite this, none of the studies reviewed in this analysis performed an *a priori* sample size calculation, which represents a significant methodological limitation that compromises the reliability of the findings.

#### The menstrual cycle as a confounding factor

A significant limitation in comparing microbiome composition between women with and without endometriosis across available studies is the insufficient attention given to the phase of the menstrual cycle during sample collection. While shifts in microbiome composition throughout the menstrual cycle are described as subtle in fecal samples, they are pronounced in vaginal samples (Krog *et al.*, 2022). For instance, studies have reported an increase in microbial diversity in the vaginal microbiome during menstruation, followed by a marked dominance of *Lactobacillus* spp. during the follicular and luteal phases, which strongly correlates with serum estradiol levels. Menstrual-phase samples tend to show a relatively higher abundance of taxa from the phyla *Fusobacteria*, *Proteobacteria*, *Bacteroidetes*, and *Actinobacteria*. In contrast, follicular-phase samples exhibit a notable reduction or absence of these phyla, with an increased prevalence of *Firmicutes* taxa (Kaur *et al.*, 2020).

Similarly, hormone-driven variations also affect the gut microbiome. Differences in microbial composition have been linked to testosterone levels in men and estradiol levels in women. Elevated estradiol levels, in particular, have been associated with altered gut microbial communities, characterized by increased abundance of *Bacteroidetes* and decreased *Firmicutes* (Shin *et al.*, 2019). These findings underscore the significant influence of hormonal fluctuations on microbiome composition.

Therefore, the lack of menstrual phase standardization across the included studies poses a major methodological concern. Notably, 10 out of 15 studies comparing vaginal fluid microbiome in women with and without endometriosis did not report the menstrual cycle phase during sampling or failed to collect samples consistently within the same phase. This issue severely compromises the validity and comparability of the results obtained. The same problem applies to gut microbiomes, as hormone-related variations in microbial composition prevent reliable aggregation of findings without rigorous control for menstrual cycle phases across cases and controls. Addressing this critical confounding factor in future research is essential to enhance the interpretability and robustness of microbiome studies in endometriosis.

#### The hormonal treatment issue

Differences in vaginal microbiome composition based on contraception methods have been well-documented (Shin *et al.*, 2019; Balle *et al.*, 2023). Women who do not use hormonal contraceptives exhibit significantly greater shifts in vaginal microbiome composition across the menstrual cycle compared to those using oral contraceptives. Studies investigating the influence of oral contraceptives on the vaginal microbiome have primarily reported increased levels of *Lactobacillus* species (Balle *et al.*, 2023). On the other hand, long-acting progestin-only contraceptives, such as levonorgestrel-implants, have not been shown to significantly alter the vaginal mucosal microbial environment (Balle *et al.*, 2023).

The impact of exogenous sex hormones extends beyond the vaginal microbiome, influencing the gut microbiome as well. Hormonal treatments, including oral contraceptives, have been associated with reduced bacterial richness and diversity in the gut (Kheloui *et al.*, 2023). Specifically, users of oral contraceptives demonstrate decreased alpha diversity but no significant changes in beta diversity compared to non-users.

Given these findings, the inclusion of participants undergoing hormonal treatments alongside those who are not, presents a critical confounding factor in studies comparing the vaginal fluid and gut microbiota between women with and without endometriosis. Standardized protocols controlling for hormonal treatment status are therefore essential to improve the reliability and validity of microbiome research.

#### The contamination issue for endometrial microbiome

Studying low-biomass microbial niches such as the endometrium requires meticulously designed experiments to minimize contamination, which can lead to data misinterpretation (Molina *et al.*, 2021; Odendaal *et al.*, 2024). Contaminant DNA can originate from various sources, including endometrial sampling techniques, the laboratory environment, plastic consumables, researchers, and reagents (Molina *et al.*, 2021). Furthermore, cross-contamination during microbiome sample processing, such as from other samples or sequencing runs, is a significant concern (Molina *et al.*, 2021). Recently, methodological considerations and good practice recommendations for studying the endometrial microbiome have been proposed (Molina *et al.*, 2021). Molina *et al.* (2021) emphasized that the primary challenge in sampling for endometrial microbiota analysis lies in the high risk of contamination from the lower genital tract. Most studies reviewed utilized conventional endometrial sampling devices. While some authors reported measures to avoid contact with the cervical and vaginal walls, the insertion of sampling devices through the high-microbial biomass cervicovaginal canal without a sheathed tool inevitably increases the likelihood of contamination. None of the studies employed double-sheathed catheters, which are widely used in embryo transfers and are expected to significantly reduce the risk of contamination from cervical or vaginal microbiota (Reschini *et al.*, 2022).

One study (Muraoka *et al.*, 2023) collected samples during hysterectomy, effectively avoiding contamination from the vaginal or cervical microbiota. However, details of the sampling procedure were not provided, and the study involved peri- and postmenopausal women, limiting the applicability of the findings to women of reproductive age. Additionally, there was a borderline statistical difference in age between women with and without endometriosis in the study, potentially introducing an additional confounding factor.

#### Dietary influences on the gut microbiome

Women with endometriosis often adopt lifestyle changes, including dietary modifications to help manage symptoms. In an online survey of 4087 Italian women with endometriosis, Mazza *et al.* (2023) reported that 66% of respondents altered their eating habits following diagnosis. Common dietary choices included gluten-free, anti-inflammatory, Mediterranean, and ketogenic diets, indicating that women explore diverse dietary strategies to alleviate symptoms and improve quality of life. Since dietary patterns can significantly influence gut microbiota composition (Mirhosseini *et al.*, 2024) changes in gut microbial diversity in women with endometriosis may reflect these self-management practices rather than being a direct cause of the disease itself. Studies investigating the gut microbiome should account for dietary factors as potential confounders. However, few studies reviewed in this context controlled these dietary influences, highlighting a critical gap in the existing research.

#### The issue of standardization of microbiome data collection and analysis

In recent years, substantial efforts have been devoted to developing standardized methods for microbiome research. Initiatives such as the Human Microbiome Project and various methodological guidelines have emphasized the importance of ensuring reliable, reproducible, and robust microbiome data (Bruggeling *et al.*, 2021; Tourlousse *et al.*, 2021; Szóstak *et al.*, 2022).

Large-scale studies of human gut microbiomes have demonstrated significant compositional differences across geographically distinct populations. However, most studies on the relationship between microbiome composition and health outcomes focus on single populations. This raises questions about whether geographical variations in microbiome composition translate to differences in disease susceptibility. Factors such as lifestyle, dietary habits, and antibiotic usage profoundly influence microbiome composition, complicating cross-country comparisons. In this review, most of the included studies were conducted in China (Wang *et al.*, 2018; Wei *et al.*, 2020, 2023; Chao *et al.*, 2021; Huang *et al.*, 2021; Shan *et al.*, 2021; Lu *et al.*, 2022; Yuan *et al.*, 2022; Hu *et al.*, 2023; Yang *et al.*, 2023; Chen *et al.*, 2024; Zhu *et al.*, 2024).

The reviewed studies employed diverse sampling, DNA extraction, and sequencing techniques. DNA extraction was predominantly performed using commercial extraction kits while a smaller subset of studies used alternative methods (Chao *et al.*, 2021; Yuan *et al.*, 2022; Hu *et al.*, 2023). Even among studies using commercial kits, subtle differences in protocols across laboratories can introduce variability, particularly in low-abundance samples such as mucus, tissues, or fluids (Sinclair *et al.*, 2023). Commercial kits, not optimized for a specific tissue or fluid, may lead to bias in the metagenomic analysis due to inefficient extraction, alteration in taxa abundance, and alpha and beta diversities (Galla *et al.*, 2024; Seethalakshmi *et al.*, 2024).

For taxonomic identification, most studies employed 16s rRNA gene sequencing for bacterial detection, while others PCR targeting specific genes of interest (Campos *et al.*, 2018; Muraoka *et al.*, 2023). Only the two more recent studies used shotgun metagenomics (MacSharry *et al.*, 2024; Pérez-Prieto *et al.*, 2024) that provides a broader compositional overview of multiple components of human microbiomes (Joos *et al.*, 2025). To analyze microbial functionality, shotgun sequencing has been reported as ‘*a preferable analytic approach, as it allows for direct functional annotation and analysis of metagenomes compared with amplicon sequencing*’ (Joos *et al.*, 2025).

The choice of sequencing platform often reflects the availability of instruments at the time of the study, which adds another dimension of variability.

#### Inconsistencies in bioinformatic analyses

In downstream statistical analyses, the reviewed studies varied widely in their choice of software tools and, frequently, the specific versions of those tools. Multiple approaches were exploited for filtering of the data, OTU identification and taxonomy and functional annotation, as well as for the computation of alpha and beta diversity scores. However, in many cases, the studies did not specify the versions of the tools used, adding another layer of inconsistency. These methodological discrepancies make reproducibility challenging, and complicate efforts to compare findings across studies. Standardized bioinformatic pipelines and transparent reporting of methods, including software versions, are essential for advancing microbiome research and ensuring comparability of results across different populations and research contexts. The best way to compare the results would be to reanalyze all the data using the same bioinformatic procedure together with a batch effect correction step. However, this is not possible presently since not all the studies uploaded their data and metadata on a repository to make them publicly available.

### Interpretation of the findings

A recent perspective paper published in *Nature Reviews Microbiology* highlights critical limitations in microbiome research, emphasizing challenges such as defining a ‘healthy’ microbiome and the need to account for confounding factors, niche-specific variables, and population-specific geographical findings (Joos *et al.*, 2025). These limitations directly impact the reliability and relevance of microbiome studies and may explain the inconsistencies observed in the studies reviewed here. Specifically, the changes in gut, vaginal, or endometrial microbiomes among patients with endometriosis compared to controls were found to be highly variable.

Microbiome research in endometriosis often mirrors approaches used in other fields without adequately addressing previously identified limitations. A notable example is the investigation of *Fusobacterium nucleatum* (*F. nucleatum*) which has been found to have a role in various diseases, particularly those linked to active inflammation and malignancies. Whether *F. nucleatum* acts as a driver (causative agent) or a passenger (opportunistic colonizer) remains a subject of debate. For instance, *F. nucleatum* is implicated in placental infections and preterm births, with evidence from human umbilical endothelial cell studies and mouse models suggesting its pathogenic potential. However, its presence in healthy placental microbiota raises concerns about sampling bias, as diseased tissues are more frequently studied. This suggests that *F. nucleatum* may only become pathogenic under specific conditions or involve particular strains, paralleling its role in oral health.

The association between *F. nucleatum* and colorectal cancer further illustrates the challenges of translating microbiome findings into clinical practice. While multiple studies report its presence across various stages of colorectal cancer, reproducibility issues persist due to differences in detection methods. Prevalence rates range from 3% to 56%, depending on the methodology employed (Brennan and Garrett, 2019). Diagnostic applications are further hindered by the lack of standardization cutoff values and biomarker analysis methods (Lișcu *et al.*, 2024). In preclinical models, antibiotics targeting *F. nucleatum* reduced tumor growth and bacterial burden in xenografts (Brennan and Garrett, 2019). However, these findings have seen limited clinical applications, as antibiotics may negatively impact immunotherapy efficacy or exacerbate microbial imbalances, complicating their therapeutic use (González *et al.*, 2024).

Overall, lessons from these studies underline the importance of addressing heterogeneity in: (i) specimen collection, (ii) sequencing methods, (iii) population demographics, and (iv) data analysis tools. Only a single study adjusted for multiple comparisons (Pérez-Prieto *et al.*, 2024). When the first paper on endometriosis and the gut microbiome was published, experts in other fields underscored that ‘*robust experimental approaches, be it within human cohorts or preclinical models, and reproducible results across microbiota studies are pivotal to bridge the translational gap and ensure that data are neither lost in translation nor mistranslated clinically*’ (Brennan and Garrett, 2019). It seems that we missed the opportunities to follow this suggestion and in the absence of robust data, we are risking being lost in translation.

## Conclusion

After analyzing the studies assessing microbiome composition in various districts of women with endometriosis, and according to the information provided by the literature that addresses technical confounders of microbiome analysis, the main conclusion of this review was the mandatory need to standardize study designs. The calculation of power and sample size, the recruiting of an adequate number of participants and samples, the reduction of interpersonal confounders and the use of adequate computational methodological approaches are critical for the advancement of knowledge in this field. Alternatively, all attempts to interpret the tremendously huge and different dimensions of a microbiome will fail to approach its full potential. This review aimed to highlight the existing research on this subject and provide insights that may help guide future directions to enhance the applicability of obtained results in the future.

## Supplementary Material

hoaf061_Supplementary_Data

## Data Availability

All data collected during the development of this work have been published alongside the manuscript, either within the main tables or as supplementary material.
