# Supplementary material for: Microbiome dysbiosis and endometriosis: a systematic scoping review of current literature and knowledge gaps
Source: Hum Reprod Open. 2025 Oct 1;2025(4):hoaf061. doi: 10.1093/hropen/hoaf061 (PMC12596503; doi:10.1093/hropen/hoaf061)
Supplement: hoaf061_Supplementary_Data [file hoaf061_supplementary_data.zip › Supplementary Table S1.docx]

**Supplementary Table S1**: Glossary of key microbiome terms

| **Term** | **Definition** |
| --- | --- |
| 16S rRNA Gene Sequencing | A targeted sequencing method that amplifies and sequences the 16S ribosomal RNA gene, conserved among bacteria but variable enough to distinguish taxa. |
| Alpha Diversity | A measure of microbial diversity within a single sample, reflecting species richness (number of species) and evenness (relative abundance). Common indices: Shannon, Simpson, Chao1. |
| Amplicon Sequence Variants (ASVs) | High-resolution sequence groupings differing by as little as one nucleotide, used instead of OTUs in modern bioinformatics (e.g., DADA2, Deblur). |
| Beta Diversity | A measure of microbial diversity between samples, reflecting differences in community composition. Metrics include Bray–Curtis dissimilarity, UniFrac (weighted/unweighted). |
| Bioinformatics Pipeline | The computational workflow used to process raw sequencing data. Includes quality filtering, taxonomic assignment, diversity calculation, and visualization. Common tools: QIIME2, DADA2, Mothur. |
| Contamination Control | Measures taken to avoid exogenous DNA contamination during sampling, extraction, and sequencing (e.g., negative controls, sterile swabs). |
| Dysbiosis | An imbalance or maladaptation in the microbial community, often associated with disease. Can manifest as reduced diversity or overrepresentation of specific taxa. |
| LEfSe (Linear Discriminant Analysis Effect Size) | A tool that identifies statistically significant features (e.g., taxa) that differ between classes (e.g., disease vs. healthy) while estimating effect size. |
| Microbiome | The total community of microorganisms (bacteria, fungi, viruses, etc.) in a given environment, including their genes and functions. |
| Operational Taxonomic Units (OTUs) | Clusters of sequences grouped based on similarity (often 97%), traditionally used to approximate microbial species. |
| PERMANOVA (Permutational Multivariate Analysis of Variance) | A statistical test used to evaluate beta diversity differences across groups. Operates on distance matrices (e.g., UniFrac). |
| QIIME2 | A widely used, modular bioinformatics platform for microbiome analysis. Supports ASV workflows, phylogenetics, diversity metrics, taxonomic classification, and visualization. |
| Rarefaction | A normalization technique that randomly subsamples reads to equalize sequencing depth across samples. Helps avoid biased diversity metrics. |
| Sequencing Depth | The number of sequencing reads obtained for a sample. Higher depth increases the likelihood of detecting rare species but may introduce cost and complexity. |
| Shotgun Metagenomic Sequencing | Sequencing of total DNA in a sample, enabling detection of all organisms and functional profiling (e.g., metabolic pathways). |
| Taxonomic Profiling | Identification of bacteria at various taxonomic levels (phylum, class, order, family, genus, species) using sequencing data. |
