# Supplementary material for: Microbiome dysbiosis and endometriosis: a systematic scoping review of current literature and knowledge gaps
Source: Hum Reprod Open. 2025 Oct 1;2025(4):hoaf061. doi: 10.1093/hropen/hoaf061 (PMC12596503; doi:10.1093/hropen/hoaf061)
Supplement: hoaf061_Supplementary_Data [file hoaf061_supplementary_data.zip › Supplementary Table S2.docx]

**Supplementary Table S2:** Overview of bioinformatic analyses across the various sample types in the included studies

|  | **SAMPLE TYPE** | **AUTHOR, YEAR** | **FILTERING** | **CHIMERIC SEQUENCES REMOVAL** | **OTUS IDENTIFICATION** | **TAXONOMY ANNOTATION** | **ALPHA AND BETA DIVERSITY COMPUTATION** | **ALPHA DIVERSITY** | **BETA DIVERSITY** | **METABOLIC AND FUNCTIONAL ANNOTATION** | **ADDITIONAL FILTERS** |  |
| --- | --- | --- | --- | --- | --- | --- | --- | --- | --- | --- | --- | --- |
|  | **STOOL / ANAL FLUID** | Marcos et al., 2024 | DADA2. Trimming of the first 15 bases at the 5′end of the sequences. Samples with a mean Phred quality score below 20 were excluded | NR | QIIME2 v2022.8 | VSEARCH with Greengenes 13.8 database | QIIME2 2022.8 | Richness and Shannon index | UniFrac distance, Jaccard distance, and Bray–Curtis dissimilarity | NR | Rarefaction to a sample depth of 3400 sequences per sample |  |
|  |  | Jimenez et al., 2024 | QIIME 2 v2022.2. Bowtie2 against hg19 reference human genome. DADA2. Trimming to forward 240 and reverse 220 nt | NR | QIIME 2 v2022.2 | q2-feature-classifier against the Genome Taxonomy Database (GTDB), version r202. q2-clawback. Qiita | MicrobiomeAnalyst 2.0 | Shannon diversity index and Chao1 | Bray–Curtis dissimilarity index | NR | Sequences that failed to be classified at the phylum level, ASVs with less than 0.01% relative abundance and 10% prevalence were filtered out. Rarefaction was applied to avoid introducing library size bias. |  |
|  |  | Do et al., 2024 | QIIME2 | QIIME2 | DADA2 | Greengenes-reference database | QIIME2 | Faith’s PD and observed features | Bray-Curtis distance | NR | Any features which contained host DNA such as mitochondria and chloroplasts were excluded |  |
|  |  | Guo et al., 2024 | FLASH | UCHIME | VSEARCH | NR | QIIME | Shannon diversity index, Simpson index, Pielou’s evenness and Good’s coverage indexes | Bray-Curtis distance | PICRUSt2 in the KEGG databases | NR |  |
|  |  | Hicks et al., 2024 | NR | DADA2 | NR | Greengenes 13.5 database | QIIME2 | Shannon diversity index | Bray–Curtis dissimilarity | NR | Rarefaction to a sample depth of 49 000 reads/sample Differentially abundance taxa identified with LEfSe. |  |
|  |  | Perez-Prieto et al., 2024 | SOAP2.21 | NR | CD-HIT | DIAMOND (v0.9.9.110) with NCBI-nr database (201,810) | NR | Shannon diversity index and observed richness | Bray Curtis dissimilarity | Benjamini–Hochberg false discovery rate for multiple correction. KEGG and eggNOG databases | Metagenomic assembly SOAPdenovo (v. 2.04, parameters: -d 1 -M 3 -R -u –F) |  |
|  |  | Pai et al., 2023 | FLASH 1.2.11 | UCHIME | UPARSE 7.0.1090 | NR | QIIME 1.9.1 | Shannon–Wiener diversity index, Simpson diversity index, Chao1 richness estimator, Good’s coverage index | Weighted and unweighted UniFrac | PICRUSt 1.1.1 | NR |  |
|  |  | Hu et al., 2023 | NR | NR | UPARSE 7.0.1001 | NR | QIIME 1.7.0 | Shannon–Wiener diversity index, Simpson diversity index, Chao1 richness estimator, and abundance-based coverage estimators index | Weighted and unweighted UniFrac | 16S Silva database | NR |  |
|  |  | Wei et al,. 2023 | FLASH | UCHIME | UPARSE 7.1 | SILVA (SSU138) Database | NR | Shannon–Wiener diversity index, Simpson diversity index | NR | PICRUSt with COG, KEGG and eggNOG databases | NR |  |
|  |  | Huang et al., 2021 | VSEARCH | NR | “Open-Reference” clustering approach | Greengenes 13.5 database | QIIME 1.9.1 | Shannon-Wiener diversity index, Simpson diversity index, number of taxa after decontamination | Bray–Curtis distance | NR | Taxa with mean relative abundance < 0.1% and prevalence < 10% within each group were discarded |  |
|  |  | Le et al., 2021 | QIIME2 | NR | DADA2 | Greengenes 13.8 database | QIIME2 | Simpson’s evenness measure E, Simpson's diversity index, and Faith’s PD metric | Weighted and unweighted UniFrac | NR | Artifact sequences and host contamination (mitochondria, chloroplast or eukaryote) were filtered out |  |
|  |  | Shan et al., 2021 | FLASH | NR | UPARSE 7.0.1090 | RDP algorithm against the Silva (SSU128) database | Mothur pipeline.1.30.1 | Shannon-Wiener diversity index, Simpson diversity index, Sobs, and abundance-based coverage estimators index | NR | KEGG database | NR |  |
|  |  | Svensson et al., 2021 | FLASH | NR | QIIME | Greengenes database | NR | Shannon-Wiener diversity index | Bray-Curtis distance | NR | NR |  |
|  |  | Perrotta et al., 2020 | USEARCH.8 | NR | In-house pipeline | SILVA Database | NR | NR | NR | NR | NR |  |
|  |  | Ata et al., 2019 | FLASH and Bowtie2 with GRCh38.p11, Dec 2013) | NR | QIIME 1.9.0 | Naïve Bayesian rdp_classifier 2.12 RDP database | QIIME 1.9.0 | Shannon-Wiener diversity index | Bray-Curtis distance | NR | NR |  |
|  | **VAGINAL FLUID** | Marcos et al., 2024 | DADA2 | NR | QIIME2 v2022.8 | VSEARCH with Greengenes 13.8 database | QIIME2 v2022.8, Fasttree, Mafft | Richness and Shannon indexes | UniFrac distance, Jaccard distance, and Bray–Curtis dissimilarity index | NR | Rarefaction to a sample depth of 3400 sequences per sample |  |
|  |  | Jimenez et al., 2024 | QIIME 2 v2022.2. DADA2 | NR | QIIME 2 v2022.2 | q2-feature-classifier against the Genome Taxonomy Database (GTDB), version r202. q2-clawback | MicrobiomeAnalyst 2.0 | Shannon diversity index and Chao1 | Bray–Curtis dissimilarity index | NR | Sequences that failed to be classified at the phylum level, ASVs with less than 0.01% relative abundance and 10% prevalence were filtered out. Rarefaction was applied to avoid introducing library size bias. |  |
|  |  | Do et al., 2024 | QIIME2 | QIIME2 | DADA2 | Greengenes database | QIIME2 | Faith’s PD and observed features | Bray-Curtis distance | NR | Any features which contained host DNA such as mitochondria and chloroplasts were excluded |  |
|  |  | MacSharry et al., 2024 | BBduk | NR | UniRef 90 database | NR | NR | Simpson index, Chao1 index and Shannon index | Bray-Curtis dissimilarity index | MetaCyc | Unassembled sequencing reads were directly analysed CosmosID-HUB Microbiome Platform |  |
|  |  | Hicks et al., 2024 | QIIME2 | DADA2 | NR | Greengenes 13.5 database | QIIME2 | Shannon diversity index | Bray–Curtis dissimilarity index | NR | Rarefaction to a sample depth 46 000 reads/sample Differentially abundance taxa identified with LEfSe. |  |
|  |  | Sessa et al., 2024 | QIIME 2.0 | NR | QIIME 2.0 | NR | QIIME 2.0 | Faith’s PD and Shannon’s diversity index | Unweighted and weighted UniFrac | NR | NR |  |
|  |  | Yang et al., 2023 | Mothur V1.33.3 | NR | QIIME’s UCLUST program | Greengenes 13.8 database | QIIME | Chao index | Bray-Curtis distance | PICRUSt2 |  |  |
|  |  | Muraoka et al., 2023 | - | - | - | - | - | - | - | - | - |  |
|  |  | Lu et al., 2022 | NR | NR | NR | NR | QIIME 2.0 | Shannon-Wiener diversity index and Chao1 index | Unweighted UniFrac distance | NR | NR |  |
|  |  | Le et al., 2021 | QIIME2 | NR | DADA2 | Greengenes 13.8 database | QIIME2 | Simpson’s evenness measure E, Simpson’s index diversity, and Faith’s PD metrix | Weighted and unweighted UniFrac | NR | Artifact sequences and host contamination (mitochondria, chloroplast or eukaryote) were filtered out |  |
|  |  | Chao et al., 2021 | NR | NR | NR | Greengenes database | QIIME 1.7.0 | Observed-species | Weighted UniFrac distance | PICRUSt | Differentially abundance taxa identified with LEfSe. |  |
|  |  | Wei et al., 2020 | Mothur V1.33.3 | NR | NR | NR | NR | NR | NR | NR | NR |  |
|  |  | Hernandes et al., 2020 | EncodeTools Metabarcode Pipeline | Deblur and VSEARCH | BLAST tool and in-house sequences | EncodeTools Metabarcode Taxonomy Assignment | Phyloseq R package | Shannon-Wiener diversity index and Simpson | Bray–Curtis dissimilarity index and weighted UniFrac | NR | Oligotypes below a frequency of 0.2% in the samples were removed. If any oligotype was observed in the negative controls, it was checked against the samples and removed from the results. |  |
|  |  | Perrotta et al., 2020 | USEARCH.8 | NR | In-house pipeline | SILVA Database | NR | NR | NR | NR | NR |  |
|  |  | Ata et al., 2019 | prinseq-lite program. FLASH Bowtie2 with GRCh38.p11, Dec 2013 | NR | QIIME 1.9.0 | Naïve Bayesian rdp_classifier 2.12 | QIIME 1.9.0 | Shannon-Wiener diversity index | Bray-Curtis dissimilarity index | NR | NR |  |
|  | **CERVICAL MUCUS/FLUID** | Yang et al., 2023 | Mothur V1.33.3 | NR | QIIME | Greengenes 13.8 database | QIIME | Chao index | Bray-Curtis distance | PICRUSt2 | NR |  |
|  |  | Yin-Yi Chang et al., 2022 | FLASH and QIIME | UCHIME | Greengenes database | RDP classifier based on SILVA Database | NR | Shannon-Wiener diversity index and Chao1 | Weighted and unweighted UniFrac | KEGG ORTHOLOGY database | NR |  |
|  |  | Huang et al., 2021 | VSEARCH | NR | “Open-Reference” clustering approach | Greengenes 13.5 database | QIIME 1.91 | Shannon-Wiener diversity index Simpson index, number of taxa after decontamination | Bray–Curtis distance | NR | Taxa with mean relative abundance < 0.1% and prevalence < 10% within each group were discarded |  |
|  |  | Wei et al., 2020 | Mothur 1.33.3 | NR | NR | NR | NR | NR | NP | NR | NR |  |
|  |  | Akiyama et al., 2019 | QIIME 1.8.0 | NR | Greengenes database | NR | QIIME | Shannon Index | Weighted UniFrac | NR | NR |  |
|  |  | Ata et al., 2019 | prinseq-lite program. FLASH Bowtie2 with GRCh38.p11, Dec 2013 | NR | QIIME 1.9.0 | Naïve Bayesian rdp_classifier 2.12 | QIIME 1.9.0 | Shannon-Wiener diversity index | Bray-Curtis distance | NR | NR |  |
|  |  | Campos et al., 2018 | - | - | - | - | - | - | - | - | - |  |
|  | **PERITONEAL FLUID** | Malvezzi et al., 2025 | bcl2fastq 2.2.0 and BiomeHub pipeline (Encodetools) | Deblur 1.1.0 and VSEARCH v2.13.6 | NR | Encoderef16s_rev6_190325; BiomeHub database (manually constructed strarting from NCBI database) | Not computed | Non computed | Non computed | NR | NR |  |
|  |  | Zhu et al., 2024 | NR | NR | NR | NR | QIIME2 | Chao1 index and Shannon-Wiener diversity index | Unweighted Unifrac distance | KEGG database | NR |  |
|  |  | Yuan et al., 2022 | FLASH 1.2.11, QIIME | UCHIME 4.2.40 | USEARCH 7.0.1090 | Greengene 13.5 database | MOTHUR 1.31.2 and QIIME 1.8.0 | Shannon-Wiener diversity and Simpson indixes | Unweighted UniFrac ditstance | NR | NR |  |
|  |  | Huang et al., 2021 | VSEARCH | NR | “Open-Reference” clustering approach | Greengenes 13.5 database | QIIME 1.91 | Shannon-Wiener diversity index, Simpson index, number of taxa after decontamination | Bray–Curtis dissimilarity index | NR | Taxa with mean relative abundance < 0.1% and prevalence < 10% within each group were discarded |  |
|  |  | Lee et al., 2021 | NR | NR | CD–HIT algorithm | UCLUST and QIIME against the Greengenes 8.15.13 database | QIIME | Shannon-Wiener diversity index, Simpson diversity, and the Chao1 indexes | Bray–Curtis dissimilarity index | NR | NR |  |
|  |  | Wei et al., 2020 | Mothur V1.33.3 | NR | NR | NR | NR | NR | NR | NR | NR |  |
|  |  | Wang et al., 2018 | NR | NR | NR | NR | NR | NR | NR | NR | NR |  |
|  |  | Campos et al., 2018 | - | - | - | - | - | - | - | - | - |  |
|  | **UTERINE FLUID** | Marcos et al., 2024 | DADA2. Trimming of the first 15 bases at the 5′end of the sequences. Samples with a mean Phred quality score below 20 were excluded | NR | QIIME2 2022.8 | VSEARCH Greengenes 13.8 database | QIIME2 2022.8, Fasttree and Mafft | Richness and Shannon indexes | UniFrac distance, Jaccard distance, and Bray–Curtis dissimilarity index | NR | Rarefaction to a sample depth of 3400 sequences per sample |  |
|  |  | Zhu et al., 2024 | NR | NR | NR | NR | QIIME2 | Chao1 index and Shannon-Wiener diversity index | Unweighted Unifrac distance | KEGG database | NR |  |
|  |  | Wei et al., 2020 | Mothur V1.33.3 | NR | NR | NR | NR | NR | NR | NR | NR |  |
|  |  | Khan et al., 2016 | NR | NR | NR | NR | NR | NR | NR | NR | NR |  |
|  | **OVARIAN CYST FLUID** | Khan et al., 2016 | NR | NR | NR | NR | NR | NR | NR | NR | NR |  |
|  | **OROPHARYNGEAL FLUID** | Marcos et al., 2024 | DADA2. Trimming of the first 15 bases at the 5′end of the sequences. Samples with a mean Phred quality score below 20 were excluded | NR | QIIME2 2022.8 | VSEARCH Greengenes 13.8 database | QIIME2 2022.8, Fasttree and Mafft | Richness and Shannon indexes | UniFrac distance, Jaccard distance, and Bray–Curtis dissimilarity | NR | Rarefaction to a sample depth of 3400 sequences per sample |  |
|  |  | Hicks et al., 2024 | QIIME2 | DADA2 | NR | Greengenes 13.5 database | QIIME2 | Shannon diversity index | Bray–Curtis dissimilarity index | NR | Rarefaction to a sample depth of 86000 reads/sample. Differentially abundance taxa identified with LEfSe. |  |
|  | **EUTOPIC ENDOMETRIUM** | Guo et al., 2025 | NR | NR | QIIME2 2019.4 and DADA2 | QIIME2 2019.4 | QIIME2 2019.4 | Chao1, Shannon, Simpson and Faith_pd diversity index | Not specified | PICRUSt2 | NR |  |
|  |  | Marcos et al., 2024 | DADA2. Trimming of the first 15 bases at the 5′end of the sequences. Samples with a mean Phred quality score below 20 were excluded | NR | QIIME2 2022.8 | VSEARCH Greengenes 13.8 database | QIIME2 2022.8, Fasttree and Mafft | Richness and Shannon indexes | UniFrac distance, Jaccard distance, and Bray–Curtis dissimilarity index | NR | Rarefaction to a sample depth of 3400 sequences per sample |  |
|  |  | Muraoka et al., 2023 | - | - | - | - | - | - | - | - | - |  |
|  |  | Wessels et al., 2021 | sl1p pipeline | NR | NR | NR | sl1p pipeline and QIIME 1.9.1-dev | Observed species, Chao1, and Shannon-Wiener Diversity index | Bray–Curtis dissimilarity index | NR | NR |  |
|  |  | Khan et al., 2021 | NR | NR | NR | Naphele 2.0 and QIIME1 | QIIME1 | Shannon-Wiener diversity index | Weighted Unifrac | NR | NR |  |
|  |  | Hernandes et al., 2020 | EncodeTools Metabarcode Pipeline | Deblur and VSEARCH | BLAST tool and in-house sequences | EncodeTools Metabarcode Taxonomy Assignment | Phyloseq R package | Shannon-Wiener diversity index and Simpson | Bray–Curtis dissimilarity index and weighted UniFrac | NR | Oligotypes below a frequency of 0.2% in the samples were removed. If any oligotype was observed in the negative controls, it was checked against the samples and removed from the results. |  |
|  | **ENDOMETRIOTIC TISSUE** | Chen et al., 2024 | NR | NR | QIIME2 | NR | QIIME2 | Chao1 and Shannon indexes | Unweighted Unifrac distance | LC–MS assay | NR |  |
|  |  | Hu et al., 2023 | NR | NR | Uparse 7.0.1001 | NR | QIIME 1.7.0 | Observe-species, Chao1, Shannon-Wiener diversity index, Simpson, abundance-based coverage estimators, and good coverage | Weighted and unweighted UniFrac | 16S Silva database | NR |  |
|  |  | Muraoka et al., 2023 | - | - | - | - | - | - | - | - | - |  |
|  |  | Hernandes et al., 2020 | EncodeTools Metabarcode Pipeline | Deblur and VSEARCH | BLAST tool and in-house sequences | EncodeTools Metabarcode Taxonomy Assignment | Phyloseq R package | Shannon-Wiener diversity index and Simpson | Bray–Curtis dissimilarity index and weighted UniFrac | NR | Oligotypes below a frequency of 0.2% in the samples were removed. If any oligotype was observed in the negative controls, it was checked against the samples and removed from the results. |  |
|  |  | Campos et al., 2018 | - | - | - | - | - | - | - | - | - |  |
|  |  |  |  |  |  |  |  |  |  |  |  |  |
|  | **Legend:** CST: Capsular Sequence Typing; LCA: lowest common ancestor; PCoA: Principal Coordinates Analysis; OTU: Operational taxonomic units; NP: Not performed; NR: Not reported | | | | | |  |  |  |  |  |  |
