# Supplementary material for: Microbiome dysbiosis and endometriosis: a systematic scoping review of current literature and knowledge gaps
Source: Hum Reprod Open. 2025 Oct 1;2025(4):hoaf061. doi: 10.1093/hropen/hoaf061 (PMC12596503; doi:10.1093/hropen/hoaf061)
Supplement: hoaf061_Supplementary_Data [file hoaf061_supplementary_data.zip › Supplementary Table S3.docx]

**Supplementary Table S3:** Taxonomy (phylum, class, order, family) across the various sample types in the included studies

| **SAM**  **PLES** | **AUTHOR, YEAR** | **HIGHER ABUNDANCE IN ENDOMETRIOSIS** | | | | **LOWER ABUNDANCE IN ENDOMETRIOSIS** | | | |
| --- | --- | --- | --- | --- | --- | --- | --- | --- | --- |
|  |  | PHYLUM | CLASS | ORDER | FAMILY | PHYLUM | CLASS | ORDER | FAMILY |
| **STOOL / ANAL FLUID** | Guo et al., 2024 | Proteobacteria | Gammaproteobacteria | Sphingomonadales | Sphingomonadaceae | Bacteroidota | Negativicutes | Veillonellales | Dialisteraceae |
|  |  |  | Alphaproteobacteria | Burkholderiales_592524 | Burkholderiaceae_A_580492 | Firmicutes_C | Bacteroidia | Bacteroidales | Bacteroidaceae |
|  |  |  |  |  |  | Bacteroidota |  |  | Acutalibacteraceae |
|  |  |  |  |  |  | Firmicutes_A |  |  |  |
|  | Do et al., 2024 |  |  |  |  |  |  |  |  |
|  | Hicks et al., 2024 |  |  |  |  |  |  | RF32 | Victivallaceae |
|  |  |  |  |  |  |  |  | RF39 | Bacteroidales |
|  |  |  |  |  |  |  |  |  | Peptococcaceae |
|  |  |  |  |  |  |  |  |  | Clostridiales |
|  | Perez-Prieto et al., 2024 |  |  |  |  |  |  |  |  |
|  | Jimenez et al., 2024 |  |  |  | Eggerthellacea |  |  |  |  |
|  | Marcos et al., 2024 |  |  |  | Enterobacteriaceae |  |  |  |  |
|  | Pai et al., 2023 |  | Erysipelotrichia | Erysipelotrichales | Micrococcaceae |  |  |  | Mariniflaceae |
|  |  |  |  | Micrococcales | Erysipelotrichaceae |  |  |  |  |
|  | Hu et al., 2023 | Proteobacteria |  |  |  | Firmicutes |  |  |  |
|  |  | Pseudomonas |  |  |  |  |  |  |  |
|  | Wei et al,. 2023 | Desulfobacterota | Desulfovibrionia | Eubacteriales | Eggerthellaceae |  | KD4-96 |  | Peptostreptococcaceae |
|  |  |  |  | Desulfovibrionales | Desulfovibrionaceae |  |  |  | Burkholderiaceae |
|  |  |  |  |  | Anaerovoracaceae |  |  |  | Pseudomonadaceae |
|  |  |  |  |  | Eubacteriaceae |  |  |  |  |
|  |  |  |  |  | Oxalobacteraceae |  |  |  |  |
|  |  |  |  |  | Ethanoligenenaceae |  |  |  |  |
|  | Huang et al., 2021 |  |  |  |  |  | Clostridia | Clostridiales | Lachnospiraceae |
|  |  |  |  |  |  |  |  |  | Ruminococcaceae |
|  |  |  |  |  |  |  |  |  |  |
|  | Le et al., 2021 | Firmicutes |  |  |  |  |  |  |  |
|  |  |  |  |  |  |  |  |  |  |
|  | Shan et al., 2021 | Actinobacteria |  |  | Tenericutes |  |  |  |  |
|  |  | Cyanobacteria |  |  |  |  |  |  |  |
|  |  | Saccharibacteria |  |  |  |  |  |  |  |
|  |  | Fusobacteria |  |  |  |  |  |  |  |
|  |  | Acidobacteria |  |  |  |  |  |  |  |
|  | Svensson et al., 2021 |  |  |  |  |  | Coriobacteria |  |  |
|  | Perrotta et al., 2020 |  |  |  |  |  |  |  |  |
|  | Ata et al., 2019 |  |  |  |  |  |  |  |  |
| **VAGINAL FLUID** | Marcos et al., 2024 |  |  |  |  |  |  |  | Enterobacteriaceae |
|  | Jimenez et al., 2024 |  |  |  |  |  |  |  |  |
|  | Do et al., 2024 |  |  |  |  |  |  |  |  |
|  | MacSharry et al., 2024 |  |  |  |  |  |  |  |  |
|  | Hicks et al., 2024 |  |  |  |  |  |  |  | Weeksellaceae |
|  | Sessa et al., 2024 | Firmicutes | Clostridia | Fusobacteriales | Leptotrichiaceae | Psuedomonadota | Alphaproteobacteria | Rhodospirillales | Rhodospirillaceae |
|  |  | Fusobacteriota | Fusobacteriia | Mycobacteriales | Mycobacteriaceae |  |  |  | Pseudomonadaceae |
|  |  |  |  | Xanthomonadales | Rhodanobacteraceae |  |  |  |  |
|  |  |  |  | Enterobacterales | Enterobacteriaceae |  |  |  |  |
|  |  |  |  |  | Megasphaeraceae |  |  |  |  |
|  | Yang et al., 2023 |  |  |  |  |  |  |  |  |
|  | Muraoka et al., 2023 |  |  |  |  |  |  |  |  |
|  | Lu et al., 2022 | Actinobacteria |  |  | Lachnospiraceae | Firmicutes |  |  |  |
|  |  | Bacteroidetes |  |  |  |  |  |  |  |
|  | Le et al., 2021 | Firmicutes |  |  |  |  |  |  |  |
|  | Chao et al., 2021 | Bacteroidetes | Clostridia | Clostridiales |  | Firmicutes | Bacilli | Lactobacillales | Lactobacillaceae |
|  |  | Proteobacteria | Gammaproteobacteria | Unidentified Clostridiales |  |  |  |  |  |
|  |  |  | Bacteroidia |  |  |  |  |  |  |
|  | Wei et al., 2020 |  |  |  | Veillonellaceae |  |  |  |  |
|  |  |  |  |  | Comamonodaceae |  |  |  |  |
|  |  |  |  |  | Pseudonomonadaceae |  |  |  |  |
|  |  |  |  |  | Erisypelotricaceae, |  |  |  |  |
|  |  |  |  |  | Micrococcaceae |  |  |  |  |
|  | Hernandes et al., 2020 |  |  |  |  |  |  |  |  |
|  | Perrotta et al., 2020 |  |  |  |  |  |  |  |  |
|  | Ata et al., 2019 |  |  |  |  |  |  |  |  |
| **CERVICAL MUCUS/FLUID** | Yang et al., 2023 |  |  |  |  |  |  |  |  |
|  | Chang et al., 2022 |  |  |  |  |  |  |  |  |
|  | Huang et al., 2021 |  |  |  |  |  |  |  |  |
|  | Wei et al., 2020 |  |  |  | Veillonellaceae |  |  |  | Leptotrichiaceae |
|  |  |  |  |  | Micrococcaceae |  |  |  |  |
|  |  |  |  |  | Comamonodaceae |  |  |  |  |
|  |  |  |  |  | Pseudonomonadaceae |  |  |  |  |
|  |  |  |  |  | Erisypelotricaceae |  |  |  |  |
|  |  |  |  |  | Caulobacteriaceae |  |  |  |  |
|  | Akiyama et al., 2019 |  |  |  | Enterobacteriaceae |  |  |  |  |
|  | Ata et al., 2019 |  |  |  |  |  |  |  |  |
|  | Campos et al., 2018 |  |  |  |  |  |  |  |  |
| **PERITONEAL FLUID** | Malvezzi et al., 2025 |  |  |  |  |  |  |  |  |
|  | Zhu et al., 2024 |  |  |  |  |  |  |  |  |
|  | Yuan et al., 2022 |  |  |  |  |  |  |  |  |
|  | Huang et al., 2021 |  |  |  |  |  |  | Actinomycetales | Microbacteriaceae |
|  |  |  |  |  |  |  |  | Caldilineaceae |  |
|  | Lee et al., 2021 |  |  | Pseudomonadales | Moraxellaceae |  |  |  | Veillonellaceae |
|  |  |  |  |  |  |  |  |  | Propionibacteriaceae |
|  |  |  |  |  |  |  |  |  | Actinomycetaceae |
|  | Wei et al., 2020 |  |  | Clostridiales | Micrococcaceae |  |  |  | Enterobacteriaceae |
|  |  |  |  |  | Pseudomonadaceae |  |  |  |  |
|  | Wang et al., 2018 |  |  |  |  |  |  |  |  |
|  | Campos et al., 2018 |  |  |  |  |  |  |  |  |
| **UTERINE FLUID** | Marcos et al., 2024 |  |  |  |  |  |  |  |  |
|  | Zhu et al., 2024 |  |  |  | Rikenellaceae |  |  |  |  |
|  | Wei et al., 2020 |  |  |  | Pseudomonadaceae |  |  | Clostridiales | Coriobacteriaceae |
|  |  |  |  |  | Erysipelotricaceae |  |  | Rhizobiales | Rhodobacteriaceae |
|  |  |  |  |  |  |  |  |  | Leptostrichiaceae |
|  | Khan et al., 2016 |  |  |  | Streptococcaceae |  |  |  | Lactobacillaceae |
|  |  |  |  |  | Moraxellaceae |  |  |  | Staphylococcaceae |
|  |  |  |  |  |  |  |  |  | Enterobacteriaceae |
| **OVARIAN CYST FLUID** | Khan et al., 2016 |  |  |  | Streptococcaceae |  |  |  | Lactobacillaceae  (only with GnRH) |
|  |  |  |  |  | Moraxellaceae |  |  |  |  |
|  |  |  |  |  | Enterobacteriaceae (only with GnRH) |  |  |  |  |
|  |  |  |  |  | Staphylococcaceae (only with GnRH) |  |  |  |  |
| **OROPHARYNGEAL FLUID** | Marcos et al., 2024 |  |  |  | Enterobacteriaceae |  |  |  |  |
|  | Hicks et al., 2024 |  |  |  | Actinomycetaceae |  |  |  | Peptostreptococcaceae |
|  |  |  |  |  | F16 |  |  |  | Neisseriaceae |
| **EUTOPIC ENDOMETRIUM** | Marcos et al., 2024 |  |  |  |  |  |  |  | Enterobacteriaceae |
|  | Guo et al., 2025 | Actinobacteria | Thermoleophilia | Neisseriales | Shewanellaceae | Proteobacteria |  |  |  |
|  |  | Chloroflexi | Chloroplast | Chlamydiales | Neisseriaceae | Bacteroidetes |  |  |  |
|  |  | Fusobacteria | Planctomycetia | Rickettsiales | Methylocystaceae | Firmicutes |  |  |  |
|  |  | Cyanobacteria |  | Alteromonadales | Ruminococcaceae |  |  |  |  |
|  |  | Planctomycetes |  | Gemmatales | Mitochondria |  |  |  |  |
|  |  | Tenericutes |  | Aeromonadales | Paraprevotellaceae |  |  |  |  |
|  |  |  |  |  | Lachnospiraceae |  |  |  |  |
|  |  |  |  |  | Isosphaeraceae |  |  |  |  |
|  |  |  |  |  | Aeromonadaceae |  |  |  |  |
|  |  |  |  |  | Staphylococcaceae |  |  |  |  |
|  |  |  |  |  | Thermoactinomycetaceae |  |  |  |  |
|  | Muraoka et al., 2023 |  |  |  |  |  |  |  |  |
|  | Wessels et al., 2021 | Actinobacteria |  |  | Oxalobacteraceae |  |  |  | Burkholderiaceae |
|  |  |  |  |  | Streptococcaceae |  |  |  |  |
|  | Khan et al., 2021 |  |  |  |  |  |  |  |  |
|  | Hernandes et al., 2020 |  |  |  |  |  |  |  |  |
| **ENDOMETRIOTIC TISSUE** | Chen et al., 2024 |  |  | Rhodobacterales |  | Patescibacteria | Saccharimonadia | Saccharimonadales | Akkermansiaceae |
|  |  |  |  |  |  | Verrucomicrobiota |  | Rickettsiales |  |
|  |  |  |  |  |  |  |  | Verrucomicrobiales |  |
|  | Hu et al., 2023 |  |  | Caulobacteriales | Pseudomonadaceae |  |  |  |  |
|  |  |  |  | Pseudomonadales |  |  |  |  |  |
|  | Muraoka et al., 2023 |  |  |  |  |  |  |  |  |
|  | Hernandes et al., 2020 |  |  |  |  |  |  |  |  |
|  | Campos et al., 2018 |  |  |  |  |  |  |  |  |
