# Supplementary material for: Microbiome dysbiosis and endometriosis: a systematic scoping review of current literature and knowledge gaps
Source: Hum Reprod Open. 2025 Oct 1;2025(4):hoaf061. doi: 10.1093/hropen/hoaf061 (PMC12596503; doi:10.1093/hropen/hoaf061)
Supplement: hoaf061_Supplementary_Data [file hoaf061_supplementary_data.zip › Supplementary Table S4.docx]

**Supplementary Table S4**. Risk of bias assessment (Newcastle-Ottawa Assessment Scale criteria)

| **STUDY** | **SELECTION** | | | | **COMPARABILITY** | **EXPOSURE** | | | **TOTAL SCORE** |
| --- | --- | --- | --- | --- | --- | --- | --- | --- | --- |
|  | **Q1** | **Q2** | **Q3** | **Q4** | **Q5** | **Q6** | **Q7** | **Q8** |  |
| Guo et al., 2025 | 1 | 0 | 0 | 0 | 0 | 1 | 0 | N/A | 4 |
| Zhu et al., 2024 | 1 | 0 | 0 | 1 | 0 | 1 | 1 | N/A | 4 |
| Do et al., 2024 | 1 | 0 | 0 | 1 | 0 | 0 | 1 | N/A | 3 |
| MacSharry et al., 2024 | 1 | 1 | 0 | 1 | 0 | 0 | 1 | N/A | 6 |
| Hicks et al., 2024 | 1 | 0 | 0 | 0 | 0 | 1 | 0 | N/A | 2 |
| Sessa et al., 2024 | 1 | 1 | 0 | 1 | 0 | 0 | 1 | N/A | 6 |
| Jimenez et al., 2024 | 1 | 0 | 0 | 1 | 0 | 1 | 0 | N/A | 3 |
| Perez-Prieto et al., 2024 | 1 | 0 | 0 | 0 | 0 | 0 | 0 | N/A | 1 |
| Guo et al., 2024 | 1 | 0 | 1 | 1 | 0 | 0 | 0 | N/A | 3 |
| Marcos et al., 2024 | 1 | 0 | 0 | 1 | 0 | 0 | 0 | N/A | 2 |
| Wei et al., 2020 | 1 | 0 | 0 | 1 | 0 | 1 | 1 | N/A | 4 |
| Chen et al,. 2024 | 1 | 1 | 0 | 1 | 0 | 1 | 1 | N/A | 5 |
| Pai et al., 2023 | 1 | 0 | 0 | 0 | 0 | 1 | 1 | N/A | 3 |
| Svensson et al., 2021 | 1 | 0 | 1 | 1 | 0 | 0 | 0 | N/A | 3 |
| Yuan et al., 2022 | 1 | 1 | 0 | 1 | 0 | 1 | 1 | N/A | 5 |
| Muraoka et al., 2023 | 1 | 1 | 0 | 1 | 0 | 0 | 1 | N/A | 4 |
| Wei et al., 2023 | 1 | 1 | 0 | 0 | 1 | 1 | 0 | N/A | 4 |
| Lu et al., 2022 | 1 | 0 | 1 | 1 | 0 | 1 | 1 | N/A | 5 |
| Akiyama et al., 2019 | 1 | 0 | 0 | 1 | 1 | 1 | 1 | N/A | 5 |
| Huang et al., 2021 | 1 | 0 | 1 | 1 | 0 | 1 | 1 | N/A | 5 |
| Shan et al., 2021 | 1 | 0 | 1 | 1 | 0 | 1 | 0 | N/A | 4 |
| Malvezzi et al., 2025 | 1 | 1 | 0 | 1 | 0 | 1 | 1 | N/A | 6 |
| Wessels et al., 2021 | 1 | 0 | 0 | 0 | 0 | 1 | 1 | N/A | 3 |
| Khan et al., 2021 | 1 | 1 | 0 | 0 | 0 | 1 | 1 | N/A | 4 |
| Le et al., 2021 | 1 | 0 | 0 | 1 | 0 | 0 | 1 | N/A | 3 |
| Chao et al., 2021 | 1 | 0 | 1 | 1 | 1 | 1 | 1 | N/A | 6 |
| Hu et al., 2023 | 1 | 1 | 1 | 0 | 0 | 1 | 1 | N/A | 5 |
| Lee et al., 2021 | 1 | 1 | 0 | 1 | 1 | 1 | 1 | N/A | 6 |
| Yang et al., 2023 | 1 | 1 | 1 | 1 | 0 | 1 | 1 | N/A | 6 |
| Hernandes et al., 2020 | 1 | 1 | 0 | 1 | 0 | 1 | 1 | N/A | 5 |
| Perrotta et al., 2020 | 1 | 1 | 0 | 1 | 1 | 0 | 1 | N/A | 5 |
| Chang et al., 2022 | 1 | 0 | 1 | 1 | 1 | 1 | 1 | N/A | 6 |
| Ata et al., 2019 | 1 | 1 | 1 | 1 | 1 | 1 | 0 | N/A | 6 |
| Wang et al., 2018 | 1 | 0 | 0 | 1 | 0 | 0 | 0 | N/A | 2 |
| Campos et al., 2018 | 1 | 1 | 0 | 1 | 0 | 1 | 1 | N/A | 5 |
| Khan et al., 2016 | 1 | 0 | 0 | 1 | 0 | 1 | 1 | N/A | 4 |
|  |  |  |  |  |  |  |  |  |  |
| **Questions:**  **Q1: Is the case definition adequate?** a) Yes, with independent validation*; b) Yes, e.g., record linkage or based on self-report; c) No description;  **Q2: Representativeness of the cases:** a) Consecutive or obviously representative series of cases*; b) Potential for selection bias or not stated;  **Q3: Selection of controls:** a) Community controls*; b) Hospital controls; c) No description;  **Q4: Definition of controls:** a) No history of disease (endpoint)*, b) No description of source;  **Q5: Comparability of cases and controls on the basis of the design or analysis:** a) Study controls for confounders, b) Study controls for any additional factor*;  **Q6: Ascertainment of endometriosis:** a) Secure record (e.g. surgical or research record)*, b) Structured interview where blind to case/control status*, c) Interview not blinded to case/control status, d) Written self-report or medical record only, e) No description;  **Q7: Same method of ascertainment for cases and controls:** a) Yes*, b) No;  **Q8: Non-response rate:** a) Same rate for both groups*, b) Non-respondent rate described, c) Rate different between cases and controls with no description. | | | | | | | | | |
