# Supplementary material for: Microbiome dysbiosis and endometriosis: a systematic scoping review of current literature and knowledge gaps
Source: Hum Reprod Open. 2025 Oct 1;2025(4):hoaf061. doi: 10.1093/hropen/hoaf061 (PMC12596503; doi:10.1093/hropen/hoaf061)
Supplement: hoaf061_Supplementary_Data [file hoaf061_supplementary_data.zip › Supplementary_File_S1-post adjudication clean.docx]

Supplementary file S1 - Search String used in this study

PUBMED

("endometriosis"[MeSH Terms] OR "endometriosis"[All Fields]) AND ("microbiome s"[All Fields] OR "microbiomic"[All Fields] OR "microbiomics"[All Fields] OR "microbiota"[MeSH Terms] OR "microbiome"[All Fields] OR "microbiomes"[All Fields] OR "microbiota"[All Fields] OR "microbiotas"[All Fields] OR "microbiota s"[All Fields] OR "microbiotae"[All Fields] OR "bacteria s"[All Fields] OR "bacteriae"[All Fields] OR "bacterias"[All Fields] OR "microbiology"[MeSH Subheading] OR "microbiology"[All Fields] OR "bacteria"[All Fields] OR "bacteria"[MeSH Terms] OR "bacterium"[All Fields] OR "bacterium s"[All Fields]).

EMBASE

('endometriosis'/exp OR 'endometriosis') AND ('microbiome s' OR 'microbiomic' OR 'microbiomics' OR 'microflora'/exp OR 'microbiome' OR 'microbiomes' OR 'microbiota' OR 'microbiotas' OR 'microbiota s' OR 'microbiotae' OR 'bacteria s' OR 'bacteriae' OR 'bacterias' OR 'microbiology' OR 'microbiology' OR 'bacteria' OR 'bacterium'/exp OR 'bacterium' OR 'bacterium s')

WEBofSCIENCES

('endometriosis' OR 'endometriosis') AND ('microbiome s' OR 'microbiomic' OR 'microbiomics' OR 'microflora' OR 'microbiome' OR 'microbiomes' OR 'microbiota' OR 'microbiotas' OR 'microbiota s' OR 'microbiotae' OR 'bacteria s' OR 'bacteriae' OR 'bacterias' OR 'microbiology' OR 'microbiology' OR 'bacteria' OR 'bacterium' OR 'bacterium' OR 'bacterium s') (All Fields)
